# A sequence-based 163plex microhaplotype assay for forensic DNA analysis

**DOI:** 10.3389/fgene.2022.988223

**Published:** 2022-10-05

**Authors:** Ruiyang Tao, Qi Yang, Ruocheng Xia, Xiaochun Zhang, Anqi Chen, Chengtao Li, Suhua Zhang

**Affiliations:** Shanghai Key Laboratory of Forensic Medicine, Shanghai Forensic Service Platform, Academy of Forensic Sciences, Ministry of Justice, Shanghai, China

**Keywords:** forensic genetics, microhaplotype (MH), next-generation sequencing (NGS), mixture deconvolution, ancestry inference

## Abstract

Novel genetic marker microhaplotype has led to an upsurge in forensic genetic research. This study established a 163 microhaplotype (MH) multiplex assay based on next-generation sequencing (NGS) and evaluated the assay’s performance and applicability. Our results showed that the 163 MH assay was accurate, repeatable and reliable, and could distinguish between African, European-American, Southern Asia and Eastern Asia populations. Among the 163 MH makers, 48 MHs with Ae > 3.0 in China Eastern Han were selected and confirmed to be highly polymorphic, with a combined power of discrimination of 1–8.26 × 10–44 and the combined power of exclusion in duos and trios of 1–1.26 × 10–8 and 1–8.27 × 10–16, respectively. Moreover, the mixture study demonstrated the realizability of the MHs in deconvoluting mixtures with different proportions of two to five-person. In conclusion, our findings support the use of this MH assay for ancestry inference, human identification, paternity testing and mixture deconvolution in forensic research.

## 1 Introduction

The theory and technique of forensic human identification and paternity testing are well established based on conventional markers such as short tandem repeats (STRs) (Butler et al., 2004). At present, complex kinship identification (i.e., forensic genealogy), mixture deconvolution and individual ancestry inference are the main difficulties the forensic community is striving to overcome (Kidd et al., 2018). The concept of compound biomarkers was introduced to weasel out of predicaments, such as STR-SNP, STR-INDEL and multi-SNP/InDel (i.e., microhaplotype, MH) (Oldoni and Podini, 2019). The concept of MH can be considered as short segments (<300 bp) of the genome with two or more variants (i.e., SNP) (Kidd et al., 2013). Due to the absence of repeat regions, the stutter peak no longer present among MHs. Therefore, MHs are multi-allelic loci that possess the advantages of high level of polymorphism of STRs and low mutation rate of SNPs, providing promising application value in forensic research (Kidd et al., 2018).

The application of next-generation sequencing (NGS), also known as massively parallel sequencing (MPS), in forensic DNA analysis is being productively used in sequence-based STR detecting and SNP phasing. NGS allows the determination of the cis/trans relationship among SNP alleles when heterozygous (Zhang et al., 2016). NGS supports the simultaneous typing of various genetic markers, i.e., STR and SNP, in a single reaction, allowing maximum information to be recovered from limited forensic samples (Zhang et al., 2016; Oldoni and Podini, 2019). NGS technology also provides a perfect research platform for MHs in forensic genetics, facilitating the testing and development of this new genetic marker. The high throughput of NGS sequenced data is undoubtedly another major impetus to the research of MHs, but also a challenge due to its relatively complicated data forms (Kidd et al., 2018; Oldoni and Podini, 2019).

With the popularity of the NGS platform in forensics laboratories, impressive progress has been made in MH exploration in recent years. Kidd et al. established a set of MH loci and uploaded the allele frequencies of 83 different populations to the ALFRED database (Kidd et al., 2017). Subsequent experiments by the researchers confirmed the superiority of this multiallelic marker over single SNPs, which achieved a discrimination power equivalent to CODIS STRs. Usually, the selection of studied MHs is based on two main statistics: an effective number of alleles (Ae) and informativeness (In) (Kidd et al., 2017). Ae can reveal the polymorphism level of MHs in a population and is positively correlated with the combined power of discrimination (CPD). Simultaneously, MHs with high Ae can deconvolute DNA mixtures (Kidd et al., 2013). Initial studies confirmed the practicality of MH assay as a supplemental tool to deconvolute two- and more than two-person DNA mixtures (Zhu et al., 2017; Chen et al., 2018). In is used to evaluate ancestral informativeness of ancestry informative markers (AIMs) (Kidd et al., 2017). It reflects the degree of differentiation between populations and varies with groups included in the statistical calculation. Studies have shown that MH loci with In > 0.185 are significant for biogeographic ancestry inference (Kidd et al., 2017). In addition, MHs have shown promising prospects for human identification, complex relationship testing and degraded samples testing (Zhu et al., 2019; Oldoni et al., 2020).

The purpose of this study was to establish a reproducible method for MH sequencing to provide a reference for forensic assay analysis. We explored the superiority of these new AIMs in discriminating region-specific populations and validated it over STR markers in multi-person mixtures deconvolution. Accordingly, MHs were screened in the related database to establish the MH sequencing assay, and 163 MHs were assembled into a single multiplex assay to analyze DNA with the NGS platform. The accuracy, sensitivity, and repeatability of the panel were verified. The results showed that, except for the promising potential of the mixture deconvolution capacity, the MH assay could be used as a powerful tool in human identification and paternity testing, as well as ancestry interference.

## 2 Materials and methods

### 2.1 Marker selection and primer design

The ALFRED website (http://alfred.med.yale.edu/) is a database developed by Kidd Lab and maintained by Yale Center for medical information. It was designed to make allele frequency data on human population samples readily available for use by scientific and educational communities. Based on the ALFRED website and published literature, the MH loci were screened from human autosomes. These loci are all distributed in the intron region and are within 300 bp. The standardized nomenclature of MH was also proposed by Kidd, which involves a simple root consisting of “MH” followed by the two-digit chromosome number and unique laboratory abbreviation and serial number (Kidd, 2016).

According to the physical position of the selected MH loci, the online primer design tool Ion AmpliSeq™ Designer (http://www.ampliseq.com) was used to design primers. The basic principle of multiplex amplification primers design included: (i) optimal melting temperatures; (ii) manufacturability, primers did not need to have long homopolymer stretches; and (iii) a GC nucleotide composition between 20% and 80%.

### 2.2 Sample collection and genomic DNA extraction

A total of 92 unrelated healthy China Eastern Han (CHE) individuals (52 males and 40 females) from Shanghai were enrolled. Human blood samples were obtained upon approval by the Ethics Committee at the Academy of Forensic Science, Ministry of Justice, China. Each participant provided written informed consent to participate in this study. Genomic DNA samples were extracted from peripheral blood using a QIAamp DNA Blood Mini Kit (Qiagen, Hilden, Germany). DNA was quantified using a Qubit dsDNA HS Assay kit (Thermo Fisher Scientific, Waltham, MA, United States ) and analyzed by Qubit Fluorometer (Thermo Fisher Scientific). The samples’ quality was assessed using the Agilent 2,100 bioanalyzer (Agilent Technologies, Palo Alto, CA, United States ) following the manufacturer’s instructions.

To explore the capacity of unbalanced mixture deconvolution for the MH panel, artificial mixtures composed of two–five individuals’ DNA with different ratios were prepared. The total DNA input was fixed at 1 ng. We sequenced the single-source four female samples (F1, F2, F3 and F4) and two male samples (M1 and M2) in advance to obtain the genotypes, after which the prepared mixtures were sequenced. The mixing ratio is shown in Supplementary Table S1.

### 2.3 Massively parallel DNA sequencing workflow

#### 2.3.1 Sequencing library construction

A Multiplex PCR Kit (Diying, Shanghai, China) combined with the customized MH primer mix was used to construct the sequencing library following the manufacturer’s instructions: 1) 8 µL of PCR master mix, 2 µL of PCR reaction mix, 8 µL of MH primer mix and 2 µL of genomic DNA (5 ng/μL) were used to form the “PCR-1” procedure. Thermal cycling was performed using the following conditions: denaturation for 15 min at 95°C; 24 cycles of 30 s at 95°C, 90 s at 60°C and 90 s at 72°C; extension was performed for 10 min at 72°C. 2) 1 µL of purified reaction mix was added to the amplification product of “PCR-1”, and then, 3) the “PCR-2” procedure was performed under the following conditions: 10 min at 37°C, 10 min at 50°C and 10 min at 65°C. 4) Using the AMPure XP Beads (Beckman Coulter, CA, United States ), two rounds of purification of the DNA libraries were performed. After quantification using the Qubit dsDNA HS Assay Kit and normalization and denaturation, the libraries were ready for sequencing.

To compare the mixture deconvolution ability of the MH panel with sequence-based STRs, artificial mixtures were also sequenced using a ForenSeq™ DNA Signature Prep Kit (Verogen, Inc., San Diego, United States ). The sequencing library was constructed following the manufacturer’s instructions (https://verogen.com/products/forenseq-dna-signature-prep-kit/).

#### 2.3.2 Sequencing and data processing/analysis

Sequencing was performed using the MiSeq Reagent Kit v3 (Verogen) on the MiSeq FGx™ system (Verogen) and the 2 × 150 bp paired-end setting according to the manufacturer’s protocol. After sequencing, the original FASTQ data was obtained. The data were processed as follows: (i) Illumina bcl2fastq v.2.17 (Illumina, San Diego, United States ) was applied to trim universal adaptors, calculate coverages and Q30 ratio; (ii) the Trimmomatic software v.0.4 (http://www.usadellab.org/cms/index.php?page=trimmomatic/) was used to remove low-quality reads (e.g., average Q-score < 20), yielding clean reads; (iii) the reads were aligned to the reference genome (GRCh37) using BWA-MEM (http://bio-bwa.sourceforge.net/) and SAMtools v0.1.19 (http://samtools.sourceforge.net/), and; (iv) targeted SNPs (or InDels) in 300 bp were called and combined to generate MH genotypes using the Python algorithm (https://www.anaconda.com/). The analysis thresholds were established based on a read depth ≥30× and heterozygote allele balance above 0.15 according to the MiSeq FGx standard.

Sequencing by the ForenSeq™ DNA Signature Prep Kit was conducted according to the manufacturer’s instructions. The sequencing results were analyzed automatically using the universal analysis software (UAS) of Verogen at default analysis thresholds (the minimum analytical threshold is at least 10 reads).

As a reference, STRait Razor 3.0 (https://github.com/Ahhgust/STRaitRazor/) was used to analyze the original FASTQ files and different analysis thresholds were adopted for single samples and mixtures.

### 2.4 Validation studies and quality control

To test the accuracy of the MH genotyping, gDNA 9,948 (positive control) was also sequenced by Sanger sequencing with designated constituent SNP primers. To validate the repeatability of the experiment, we constructed the DNA library of gDNA 9,948 thrice and compared the sequencing results. In addition, we also calculated the sensitivity of the assay by constructing gradient DNA input libraries (31.25pg–20 ng) for sequencing.

Sequencing quality was assessed by the depth of coverage, sequence composition ratio (allele ratio and noise ratio) and the successful ratio of sequencing (measured by the sequencing results of 1 ng input DNA).

### 2.5 Statistical analysis

#### 2.5.1 Populations structures

A total of 2,504 individuals in 26 population groups (Supplementary Table S2) were used as the reference population to evaluate the genetic distributions among the populations. SNP genotype data of individuals were downloaded from the 1,000 Genome Project Phase 3 and transformed into MH genotypes according to the sequential structure of MHs.

In this study, we used the Infocalc v1.1 program to calculate Informativeness (In) (Rosenberg et al., 2003; Rosenberg, 2005). Cluster analysis was performed using the STRUCTURE v2.3.4 program based on the MH genotype data with 10,000 steps of Length of Burnin Period and 10,000 steps of MCMC (Markov Chain Monte Carlo) (Rosenberg et al., 2002). The K subgroups were set to 2–6, and the operation was repeated 10 times. Structure Harvester (http://taylor0.biology.ucla.edu.tructharvest/) was used to infer the best K value, and repeated sampling analysis was performed on the q-matrix data with different K values using the CLUMPP v1.1.2 program (Jakobsson and Rosenberg, 2007). Lastly, the DISTRUCT v1.1 software was used to draw the bar graph of population genetic structure with different K values (Rosenberg, 2004).

The POPTREE2 software was used to calculate the F-statistics (Fst) and GraphPad Prism v9.0.1 software (https://www.graphpad.com/) to visualize the Fst distribution (Takezaki et al., 2010). The POPTREE2 software and MEGA X software were used to draw the phylogenetic tree of 27 populations based on the neighbor-joining method (NJ method) (Hall, 2013).

### 2.5.2 General parameters

The allele frequencies of each MH locus were calculated for all 92 CHE individuals. Exact tests for Hardy-Weinberg Equilibrium (HWE) as well as the forensic parameters including the matching probability (MP), power of discrimination (PD), power of paternity exclusion (PE), typical paternity index (TPI) were calculated according to previously described methods (Tao et al., 2019). The power of exclusion in duo (PED), power of exclusion in trios (PET), together with the CPD, cumulative probability of exclusion in duo (CPED) and cumulative probability of exclusion in trios (CPET) were also calculated to assess the capacity in forensic individual identification and paternity testing. Linkage disequilibrium (LD) between pairs of MH markers was performed using the Haploview software (Barrett et al., 2005).

The Familias three software was used to construct simulated father-child pairs based on the allele frequencies of CHE (Kling et al., 2012; Kling et al., 2014). The number of simulations was set to 10,000, with H1 assuming that AF (alleged father) and CH (child) were paternal and H2 assuming that AF was not the father of the CH. The likelihood ratio (LR) of H1 and H2 was calculated, and the seaborn module of Python was used to draw the kernel density map of Log10 (LR).

## 3 Results

### 3.1 Construction and optimization of the 163 plex MH system

In this study, a total of 208 MH loci distributed on 22 human autosomes were screened based on the ALFRED database and related literature. Based on the principle of multiplex amplification primer design, 26 MH loci were removed due to incomplete amplicon coverage of the primers. The remaining 182 pairs of forward and reverse primers of 182 MHs were initially designed according to the chromosomal physical position (hg19/GRCh37).

After the specificity verification of the designed primers, the concentration of the extension primers was also extensively adjusted. We first stochastically sequenced 20 DNA samples to test the overall multiplexing. However, 19 of 182 MHs failed the multiplex amplification and were removed from the panel. Thus, the final optimized panel contained 163 MH loci. The basic information of 163 MH loci is listed in Supplementary Table S3, and the sequence information of the primers is shown in Supplementary Table S4. Each MH locus consists of two to six SNPs, with an amplicon size of about 300 bp.

### 3.2 Validation studies and quality control

The accuracy of the panel was verified by Sanger sequencing using gDNA 9,948 (positive control). The results showed that the genotypes of all SNP loci in the MH system were consistent with the results of NGS. For repeatability assessment, three same samples (gDNA 9,948) were used to construct three libraries for sequencing on the MiSeq FGx platform. The results revealed consistent allele genotypes and haplotype for triple repeatability testing.

The results of DNA genotyping at all concentrations were complete and accurate. Sequencing quality control statistics with different concentration gradients of DNA input are shown in Supplementary Figure S1. The average depth of coverage (DoC) of samples with different concentration gradients was above 12,000×, and the uniformity of each DNA concentration gradient was over 57%.

The average DoC of 92 CHE samples for the studied sample sequencing was 2,301.44× (ranging from 1,000× to 4,000×). The detailed sequencing quality are shown in Supplementary Table S5.

### 3.3 Data analysis

Allele genotyping and allele frequencies of the 163 MHs in 92 CHE samples are listed in Supplementary Table S6. A total of 610 alleles were detected. It was observed that three alleles, AACC, AATG and GCCC, detected in MH11KK180 were not recorded in the ALFRED database, with the frequencies in CHE samples being 0.016, 0.033 and 0.005, respectively.

The CHE-specific Ae values for each MH and the forensic parameters are shown in Supplementary Table S7. The average Ae for the 163 MH loci was 2.5276, ranging from 1.0000 (MH02KK102, MH06KK080 and MH10KK084) to 7.0814 (MH13KK218). Among the 163 MH loci, 48 loci had Ae values greater than 3.0, and nine loci had Ae values greater than 4.0. In comparison, no heterozygotes were detected at three loci (MH02KK102, MH06KK080 and MH10KK084) of the sequenced CHE population.

### 3.4 Forensic application of the MH assay

#### 3.4.1 Ancestry inference

To evaluate the performance of the 163 MHs in ancestry inference, we calculated relevant informativeness parameters In based on 1,000 Genomes Phase 3 data (Supplementary Table S8). The informativeness In value for the 163 MHs in the 27 populations ranged from 0.0220 to 0.6504 (mean of 0.1596), 52 of 163 MH loci had relatively good ability for ancestry inference (In > 0.185) (Kidd et al., 2017).

**FIGURE 1:**
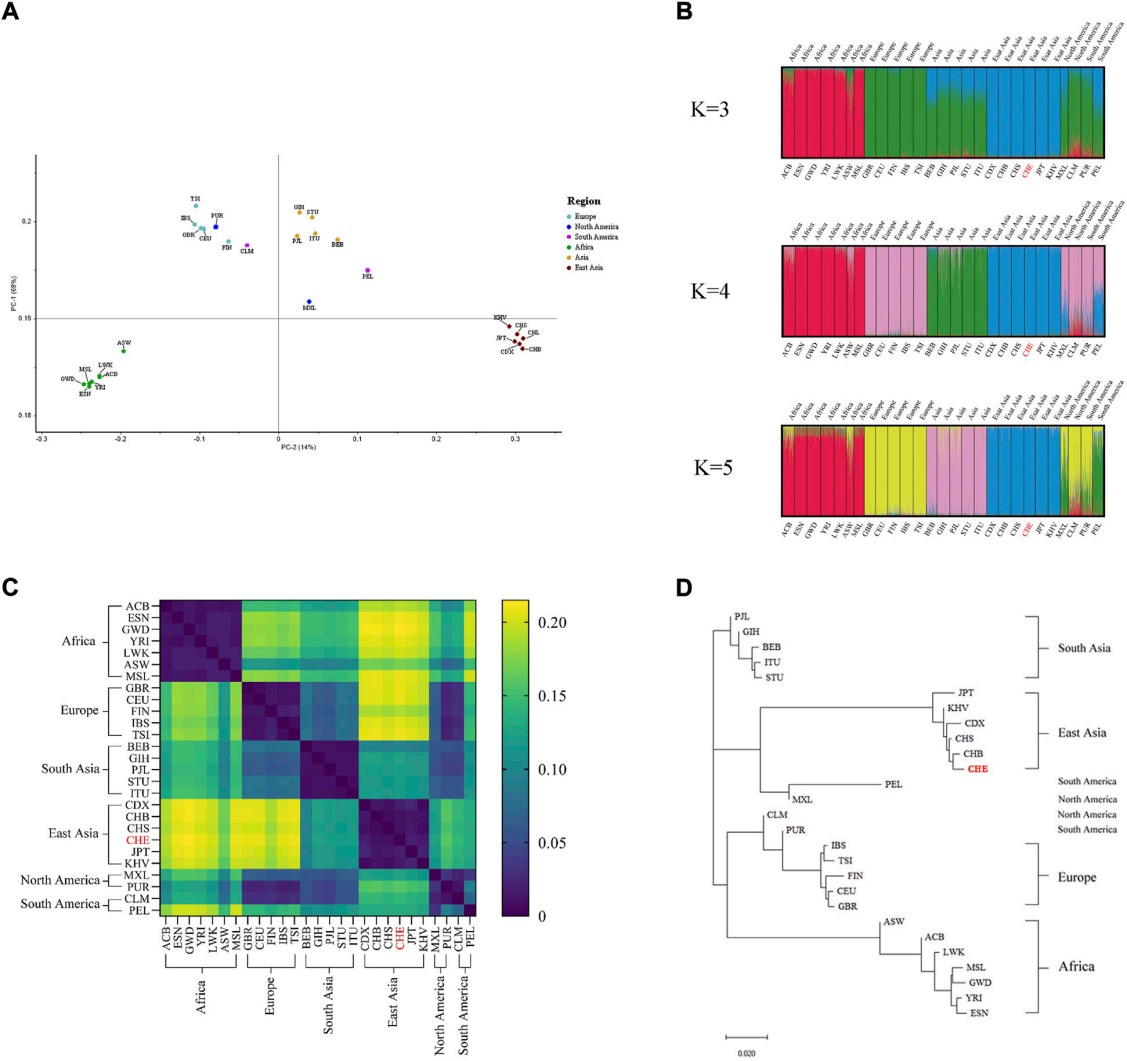
Ancestry inference analysis **(A)** PCA of 27 populations, **(B)** STRUCTURE bar plot of K values ranging from 3 to 5, **(C)** distribution of paired Fst values in 27 populations, and **(D)** NJ trees of 27 populations.

Subsequently, we used three different bioinformatics methods to evaluate the ancestry inference ability of the system. As shown in Figure 1A, principal component analyses of the 27 populations showed that PC1 accounted for 68% of the variance dividing the populations and PC2 accounted for 14%, thereby dividing the studied groups into four main clusters (African, European-American clusters, Southern Asia and Eastern Asia). The results of the STRUCTURE analysis are presented in Figure 1B. At K = 3, the African population was separated from the European and Asian clusters. At K = 4, four main clusters were observed: African component, European component, and South and East Asian components. The American population was not separated from the European population. At K = 5, no significant population stratification was observed within the four population clusters, while a unique genetic structure (green in the K = 5 STRUCTURE bar plot) was observed in the admixed American (AMR) population. Fst values between studied populations are presented in Supplementary Table S9, and the corresponding heatmap of paired Fst values in 27 populations is illustrated in Figure 1C. Of note, the GBR and CEU populations displayed strong genetic similarities with the lowest Fst value of 0.006 compared to all other population pairs. Conversely, the highest Fst value of 0.215 was found in CHE and GWD population pairs. The East Asian-African-pair and East Asian-European-pair showed the largest average Fst values across all 163 loci. The five intercontinental population groups had no clear genetic affinities, except for the American and European populations which showed a relatively low Fst value (<0.05). To intuitively determine the genetic structure relationships between the studied populations, a phylogenetic tree was constructed based on the Fst value using the NJ method (Figure 1D). The 27 studied populations were clustered in five main branches, and the population groupings corresponding to the geographical distribution generated by the NJ method were concordant with those seen in the PCA scatterplot (Figure 1A) and STRUCTURE diagram (Figure 1B).

#### 3.4.2 Individual identification and paternity testing

Here, 92 samples from the CHE population were genotyped using the 163plex MH system, and 48 MHs with an Ae ≥ 3.0 in the sequenced CHE population were selected to constitute the detection system for individual identification. According to the LD analysis result, the pair-loci r2 values of the MHs on the same chromosome were all less than 0.8, which could rule out the LD among these loci (Supplementary Figure S2).

All 48 MHs showed no significant deviation from HWE after Bonferroni correction. In the samples of CHE, the Ae values of the 48 MHs ranged from 3.0067 (MH01KK211) to 7.0814 (MH13KK218), with an average value of 3.7273. Other forensic parameters of the 48 MHs, including HE, PD, PED, PET and TPI, are listed in Table 1. The CPD of the 48 MH loci in the CHE population was 1–8.26 × 10–44. The CPED was 1–1.26 × 10–8, and the CPET was 1–8.27 × 10–16.

**TABLE 1:** Forensic parameters of 48 MH loci in the individual identification system.

No.	MH	Ae	He	PD	PED	PET	TPI
1	MH01CP008	3.2825	0.6957	0.8511	0.2746	0.4749	1.6429
2	MH01CP012	3.0628	0.6413	0.8282	0.2520	0.4435	1.3939
3	MH01CP016	3.0778	0.7174	0.8072	0.2430	0.4336	1.7692
4	MH01KK117	4.2575	0.7174	0.9126	0.3237	0.5304	1.7692
5	MH01KK205	4.1684	0.6739	0.9064	0.3595	0.5624	1.5333
6	MH01KK211	3.0067	0.6087	0.8339	0.2465	0.4414	1.2778
7	MH02KK134	3.4696	0.6739	0.8826	0.3137	0.5162	1.5333
8	MH02KK136	3.6357	0.6739	0.8726	0.3135	0.5126	1.5333
9	MH04CP002	3.6601	0.7253	0.8702	0.3029	0.5048	1.8200
10	MH04CP003	3.5069	0.7065	0.8705	0.2916	0.4930	1.7037
11	MH04CP007	3.2992	0.7500	0.8360	0.2731	0.4705	2.0000
12	MH04KK030	3.9991	0.7500	0.8757	0.3432	0.5452	2.0000
13	MH05CP004	3.8808	0.7717	0.8863	0.3314	0.5339	2.1905
14	MH05CP006	3.1653	0.6413	0.8433	0.2586	0.4535	1.3939
15	MH05KK020	3.2623	0.7065	0.8353	0.2680	0.4624	1.7037
16	MH05KK170	6.0544	0.8696	0.9402	0.4999	0.6851	3.8333
17	MH06CP003	3.5333	0.6739	0.8748	0.2919	0.4918	1.5333
18	MH06CP007	3.4660	0.7500	0.8507	0.2908	0.4926	2.0000
19	MH09KK153	4.4786	0.7363	0.9071	0.3924	0.5919	1.8958
20	MH10CP003	3.2953	0.7500	0.8440	0.2698	0.4663	2.0000
21	MH10KK163	4.2341	0.7391	0.9050	0.3760	0.5757	1.9167
22	MH11CP003	3.5766	0.7283	0.8556	0.2959	0.4968	1.8400
23	MH11CP005	3.4205	0.6739	0.8585	0.2821	0.4801	1.5333
24	MH11KK180	3.6186	0.7500	0.8828	0.3253	0.5251	2.0000
25	MH12KK046	3.9226	0.7609	0.8781	0.3184	0.5231	2.0909
26	MH12KK202	3.5811	0.7826	0.8549	0.2979	0.5001	2.3000
27	MH13CP008	3.0987	0.6630	0.8384	0.2476	0.4398	1.4839
28	MH13KK213	3.8781	0.7935	0.8698	0.3262	0.5290	2.4211
29	MH13KK217	4.5628	0.7609	0.9168	0.4011	0.6015	2.0909
30	MH13KK218	7.0814	0.7935	0.9565	0.5481	0.7237	2.4211
31	MH13KK225	3.1897	0.7283	0.8336	0.2634	0.4599	1.8400
32	MH14CP003	3.1021	0.6522	0.8476	0.2643	0.4554	1.4375
33	MH14CP004	3.7493	0.7935	0.8741	0.3140	0.5168	2.4211
34	MH15CP001	3.5257	0.6515	0.8701	0.2915	0.4915	1.4348
35	MH15KK066	3.2940	0.6630	0.8606	0.2748	0.4748	1.4839
36	MH16KK255	4.3350	0.7935	0.9057	0.3819	0.5818	2.4211
37	MH16KK302	3.8238	0.7717	0.8778	0.3239	0.5256	2.1905
38	MH17CP001	3.2883	0.6957	0.8490	0.2717	0.4684	1.6429
39	MH17CP006	3.1227	0.6630	0.8367	0.2539	0.4481	1.4839
40	MH17KK272	3.2615	0.7473	0.8468	0.2802	0.4793	1.9783
41	MH18CP003	3.4939	0.7283	0.8615	0.2864	0.4858	1.8400
42	MH18CP005	3.6953	0.7174	0.8719	0.3036	0.5055	1.7692
43	MH19CP007	3.5690	0.6848	0.8693	0.2939	0.4941	1.5862
44	MH19KK299	3.4582	0.7717	0.8594	0.3067	0.5066	2.1905
45	MH20KK058	3.6287	0.7065	0.8648	0.2982	0.4994	1.7037
46	MH20KK307	3.7352	0.7174	0.8830	0.3217	0.5232	1.7692
47	MH21KK315	4.5804	0.7439	0.9164	0.4033	0.6024	1.9524
48	MH21KK324	3.5208	0.7826	0.8478	0.3042	0.5019	2.3000

**FIGURE 2:**
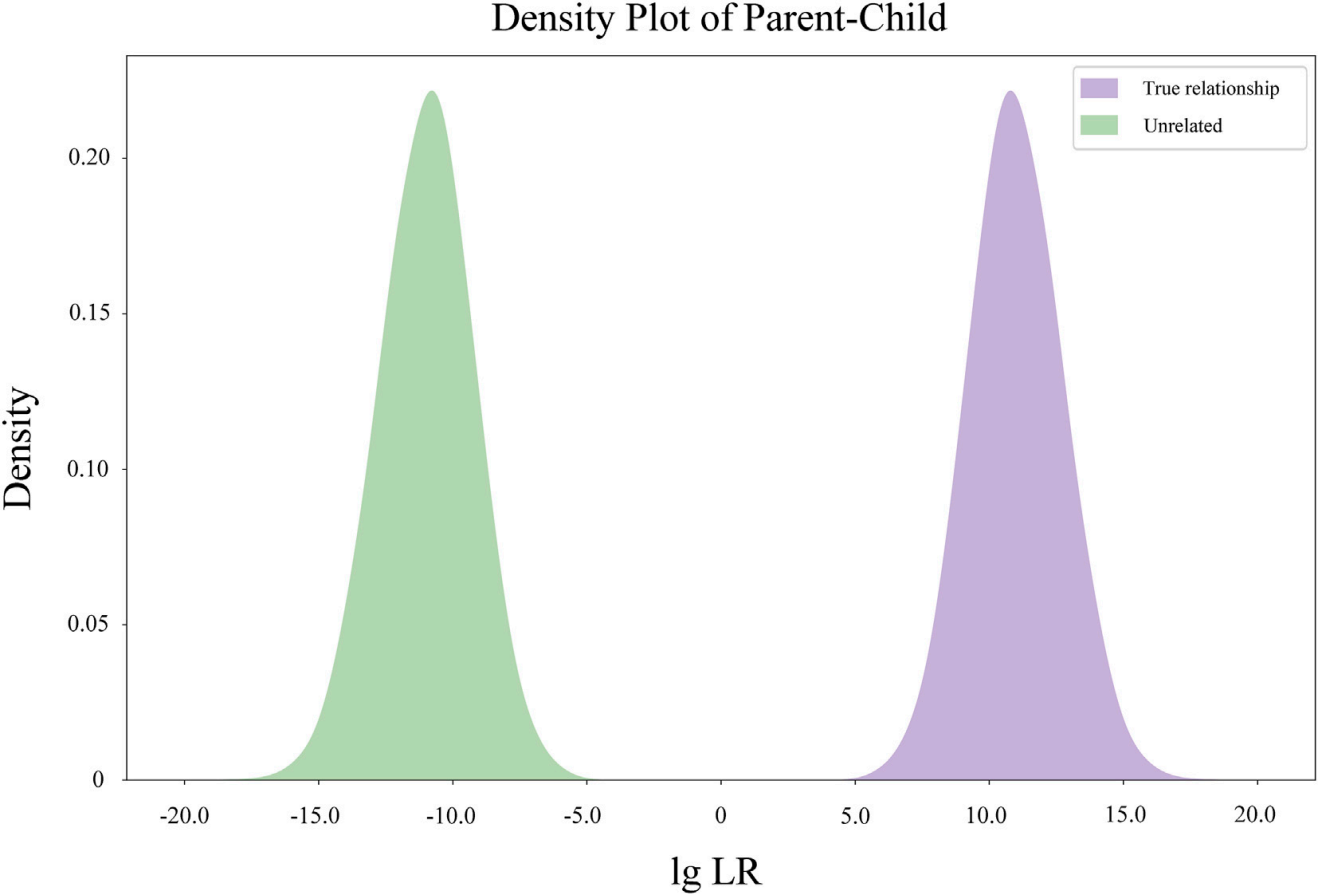
Kernel density diagram of Log10 (LR) value distribution of simulated parent-child pairs.

The Kernel density diagram of LRs of simulated parent-child pairs based on the allele frequencies of 48 MH loci is illustrated in Figure 2. No overlap was observed between the LR distribution of the two hypotheses (related-as-claimed and unrelated individuals).

#### 3.4.3 Mixture deconvolution

##### 3.4.3.1 NGS-STR analysis

In this study, we used the sequenced result statistics of artificial DNA mixtures to compare the mixture deconvolution capacity of the STR panel (ForenSeq™ DNA Signature Prep Kit) and the MH panel. Then, 27 autosomal STRs and the Amelogenin locus from ForenSeq™ DNA Signature Prep Kit were selected as the NGS-STR system for the mixture analysis. The genotypes of mixture contributors were known in advance, and the maximum number of individual STR alleles for two-, three-, four- and five-person DNA mixtures were 90, 112, 131 and 148, respectively. The maximum number of alleles detected at a single STR locus was four for the two-person mixture (CSF1PO, D16S539, D17S1301, D21S11, D2S1338, D2S441, D3S1358, D9S1122, FGA and PentaD), six for the three-person mixture (D2S1338 and FGA), seven for the four-person mixture (D12S391, D19S433 and PentaE) and eight alleles for the five-person DNA mixtures (D19S433 and FGA). The detailed NGS-STR results are shown in Table 2.

**TABLE 2:** NGS-STR analysis results of DNA mixtures.

Number of mix person	Mix ratio	Maximum alleles number	Detected alleles	Detection rate of alleles (%)	Allele number of major	Detected allele number of major	Detection rate of major contributor (%)	Allele number of minor	Detected allele number of minor	Detection rate of minor contributor (%)
2	9:1	90	81	90.00	52	52	100.00	38	29	76.32
	19:1	90	70	77.78	52	52	100.00	38	18	47.37
	49:1	90	54	60.00	52	52	100.00	38	2	5.26
	99:1	90	53	58.89	52	52	100.00	38	1	2.63
	199:1	90	52	57.78	52	52	100.00	38	0	0.00
3	7:2:1	112	84	75.00	48	48	100.00	64	36	56.25
	17:2:1	112	84	75.00	48	48	100.00	64	36	56.25
	47:2:1	112	53	47.32	48	48	100.00	64	5	7.81
	97:2:1	112	50	44.64	48	48	100.00	64	2	3.13
	197:2:1	112	48	42.86	48	48	100.00	64	0	0.00
4	5:2:2:1	131	120	91.60	46	46	100.00	85	74	87.06
	13:4:2:1	131	107	81.68	46	46	100.00	85	61	71.76
	43:4:2:1	131	67	51.15	46	46	100.00	85	21	24.71
	93:4:2:1	131	53	40.46	46	46	100.00	85	7	8.24
	193:4:2:1	131	47	35.88	46	46	100.00	85	1	1.18
	3:2:2:2:1	148	139	93.92	51	51	100.00	97	88	90.72
5	13:2:2:2:1	148	125	84.46	51	51	100.00	97	74	76.29
	35:8:4:2:1	148	111	75.00	51	51	100.00	97	60	61.86
	85:8:4:2:1	148	85	57.43	51	51	100.00	97	34	35.05
	185:8:4:2:1	148	68	45.95	51	51	100.00	97	17	17.53

Complete sequencing data from major contributors could be obtained in different mixed numbers and ratios of mixture samples (allele detection rate: 100%). However, when distinguishing minor contributors from the main contributor, the allele detection rate decreased significantly with the increase of the mixing ratio. The unique alleles of minor contributors were observed in mixing ratios of 199:1 and 197:2:1. Statistical results showed that the detection rate of minor alleles exceeded 50% only at moderately mixing ratios (two-person mixture at 9:1, three-person mixture at 7:2:1 and 17:2:1, four-person mixture at 5:2:1 and 13:4:2:1, five-person mixture at 3:2:2:1, 13:2:2:1 and 35:8:4:2:1) (Table 2). The representative STR results of mixed samples at different mix ratios are illustrated in Supplementary Figure S3–6

##### 3.4.3.2 NGS-MH analysis

Among the 48 MHs with an Ae ≥ 3.0, the overall sequencing quality of MH05KK020, MH10KK163 and MH15CP001 did not reach the quality control standard (DoC <30×). Therefore, these three loci were not included in the following mixture analysis.

For the 45 MHs, the maximum number of alleles detected by two-, three-, four- and five-person mixtures were 117, 135, 163 and 173, respectively. No more than four different alleles could be found in the mixture of two donors at the MH01KK205, MH06CP003 and MH13KK217. As for three-, four- and five-person mixtures, 6 (MH13kk217), 6 (MH13MH218), and 6 (MH13KK217, MH13KK218) different alleles were found, respectively. The statistical results of the mixture analysis of the 45 MHs are shown in Table 3.

**TABLE 3:** NGS-MH analysis results of the DNA mixtures.

Number of mix person	Mix ratio	Maximum alleles number	Detected alleles	Detection rate of alleles (%)	Allele number of major	Detected allele number of major	Detection rate of major contributor (%)	Allele number of minor	Detected allele number of minor	Detection rate of minor contributor (%)
2	9:1	117	117	100.00	78	78	100.00	39	39	100.00
	19:1	117	115	98.29	78	78	100.00	39	37	94.87
	49:1	117	109	93.16	78	78	100.00	39	31	79.49
	99:1	117	105	89.74	78	78	100.00	39	27	69.23
	199:1	117	101	86.32	78	78	100.00	39	23	58.97
3	7:2:1	135	135	100.00	73	73	100.00	62	62	100.00
	17:2:1	135	132	97.78	73	73	100.00	62	59	95.16
	47:2:1	135	125	92.59	73	73	100.00	62	52	83.87
	97:2:1	135	120	88.89	73	73	100.00	62	47	75.81
	197:2:1	135	111	82.22	73	73	100.00	62	38	61.29
4	5:2:2:1	163	163	100.00	79	79	100.00	84	84	100.00
	13:4:2:1	163	162	99.39	79	79	100.00	84	83	98.81
	43:4:2:1	163	160	98.16	79	79	100.00	84	81	96.43
	93:4:2:1	163	159	97.55	79	79	100.00	84	80	95.24
	193:4:2:1	163	142	87.12	79	79	100.00	84	63	75.00
5	3:2:2:2:1	173	173	100.00	82	82	100.00	91	91	100.00
	13:2:2:2:1	173	173	100.00	82	82	100.00	91	91	100.00
	35:8:4:2:1	173	170	98.27	82	82	100.00	91	88	96.70
	85:8:4:2:1	173	165	95.38	82	82	100.00	91	83	91.21
	185:8:4:2:1	173	159	91.91	82	82	100.00	91	77	84.62

Among the mixtures of different mixing ratios, the allele detection rate of major contributors was 100%, and the allele detection rate of minor contributors decreased with an increase in the mixing ratio. All unique alleles could be detected only amongst the 9:1 mixing ratio of two-person mixture, 5:2:2:1 mixing ratio of four-person mixture, and 3:2:2:2:1 and 13:2:2:2:1 mixing ratio of five-person mixture.

**FIGURE 3:**
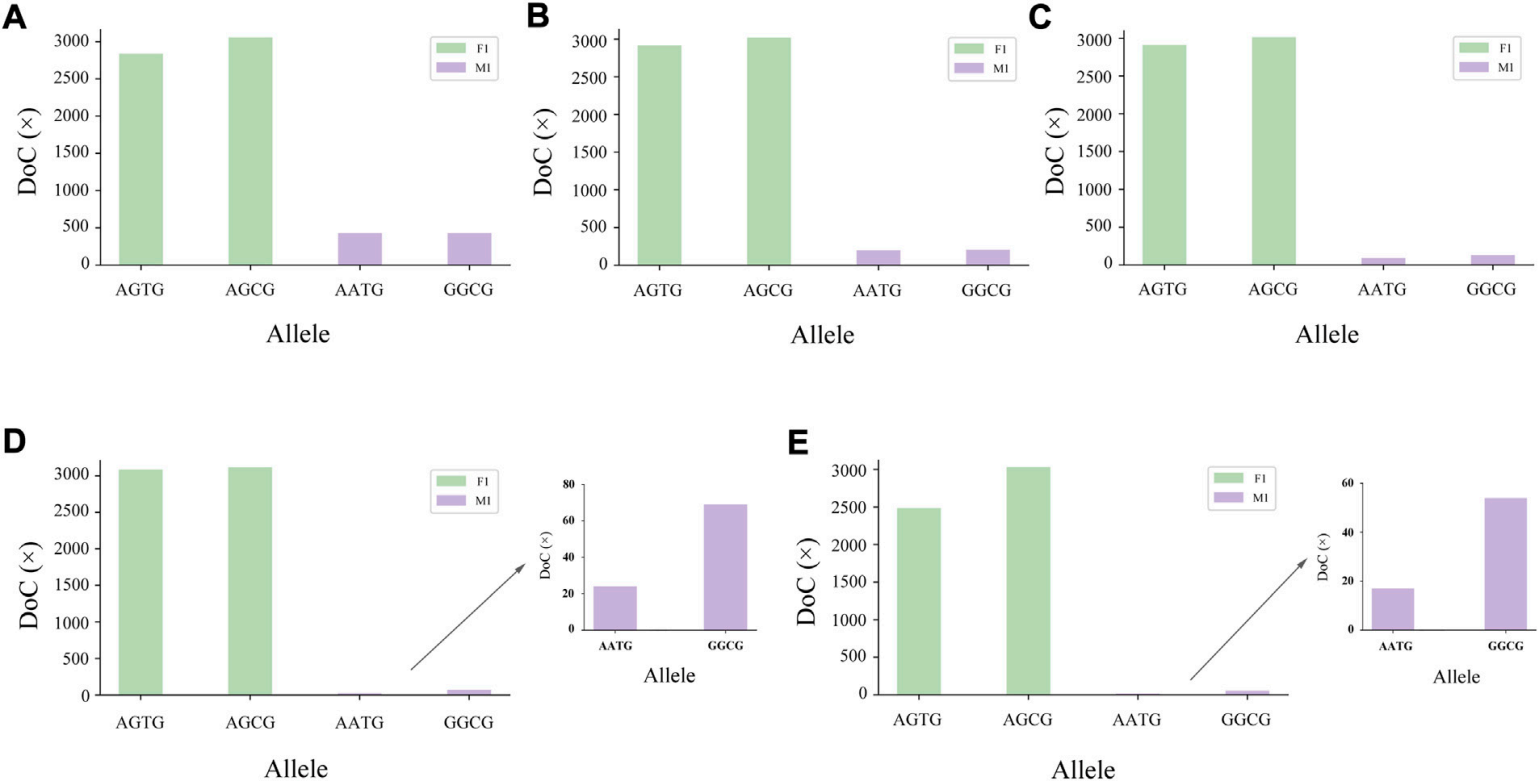
Schematic diagram of two-person mixture at MH13KK217 locus. The alleles of F1 were AGTG/AGCG, the alleles of M1 were AATG/GGCG. **(A)** Mix ratio = 9:1, **(B)** Mix ratio = 19:1, **(C)** Mix ratio = 49:1, **(D)** Mix ratio = 99:1, and **(E)** Mix ratio = 199:1.

**FIGURE 4:**
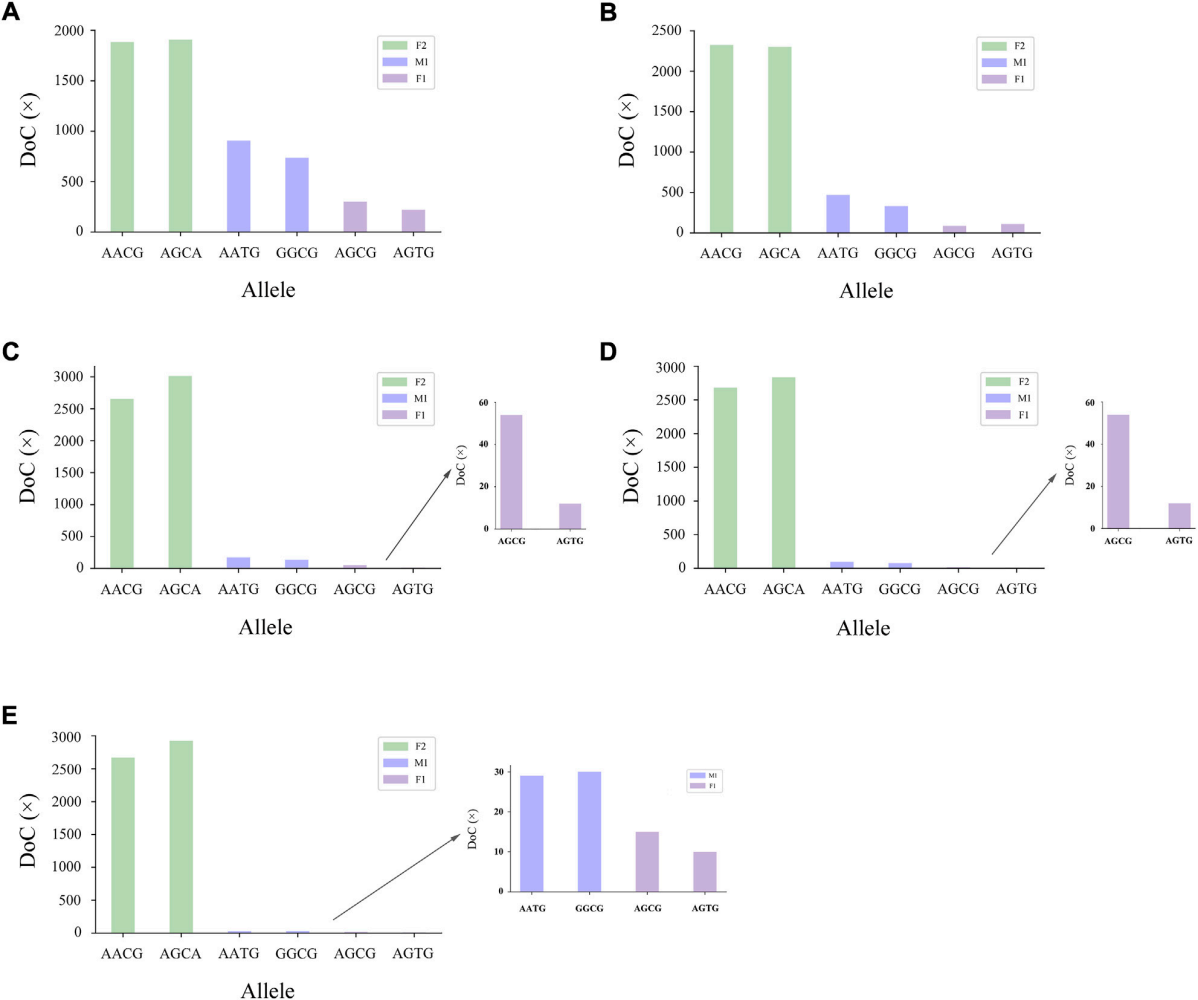
Schematic diagram of three-person mixture at MH13KK217 locus. The alleles of F2 were AACG/AGCA, the alleles of M1 were AATG/GGCG, and the alleles of F1 were AGCG/AGTG. **(A)** Mix ratio = 7:2:1, **(B)** Mix ratio = 17:2:1, **(C)** Mix ratio = 47:2:1, **(D)** Mix ratio = 97:2:1, and **(E)** Mix ratio = 197:2:1.

The genotyping diagram of the representative MH13KK217 locus in the mixture of two contributors is shown in Figure 3. As can be seen, complete genotyping data of secondary contributors were obtained under the two-person mixing ratios of 9:1, 19:1 and 49:1. In contrast, the DoC of the AATG allele of contributor M1 was lower than 30× at the mixing ratios of 99:1 and 199:1. The genotyping diagram of the three-person mixture is shown in Figure 4. Complete genotyping data of all contributors are obtained under the mixing ratios of 7:2:1 and 17:2:1. The DoC of the AGTG allele within the minimal contributor F1 was lower than 30× under the mixing ratios of 47:2:1 and 97:2:1. In the mixed ratio of 197:2:1, the allele DoC of M1 and F1 was lower than 30×.

The genotyping diagrams of four-person and five-person mixtures are shown in Supplementary Figure S7, 8, respectively. Due to the restriction of locus polymorphism, different contributors may share part of the alleles. Different patterns with the same color were used for intuitive display when simulating shared alleles according to the mixing ratio.

## 4 Discussion

NGS or MPS technology is being gradually integrated into modern forensic examinations (Haddrill, 2021). It has changed the research methodology of forensic genetics and the way genetics studies are conducted. NGS has been applied to the mainstay of STR and SNP analysis and achieved remarkable success in the forensic field. In the meantime, MHs were derived on the basis of SNP and InDel based on the characteristic of NGS (Kidd et al., 2013). Endowed with high level of polymorphism and not producing stutter, this newly developed genetic marker has been proved to have great potential in various forensic identification applications. Therefore, we attempted to construct an MH system based on the advantages of NGS and to explore a workflow combining various analysis methods to provide a new train of thought for studying forensic genetics.

In this study, we choose proper MHs for the preliminary system construction. The advanced validation study showed that the 163plex MH system constructed in this study had typing accuracy and experimental repeatability, and complete genotyping could be obtained above 0.03125 ng of DNA input. On the premise of high DNA purity, a relatively low quantity of DNA input also provided high-quality sequencing library construction. Subsequently, we sequenced 92 China Eastern Han samples in one MiSeq FGx run by multiplexing these samples in a single sequencing library. Based on the preset criteria and combined with DoC and sequence composition ratio, we evaluated the sequencing data quality to ensure the reliability of the parametric analysis.

Subsequently, the ability of intercontinental ancestry inference by our 163plex MH assay was confirmed *via* bioinformatics analysis. Studies have shown that the MH genetic markers based on SNPs were ideal markers for individual genealogical inference (Kidd et al., 2018). Informativeness parameters In defined by Rosenberg et al. is the statistic to evaluate the informativeness of AIMs that reflect the degree of differentiation difference between populations (Rosenberg, 2005). AIMs with high In can distinguish between specific populations and predict the relevant biogeographic ancestry of individuals (Kidd et al., 2017), while MHs with In value <0.185 have little value for ancestry inference. Due to the different allele frequencies of AIMs in different populations, In changed accordingly when different groups were included in the calculation. Once the calculation population was limited to the East Asian population, only MH02KK004 in our panel had In values exceeding 0.185, with bioinformatics analysis results showing no distinction within the East Asian group. This suggests that if the MH system is used in studying different populations in nearby areas, it is necessary to include MHs with higher In values in these studied populations.

Besides, the overlapping area between the European and American populations observed in the PCA diagram also confirms the similarity of genetic structures of these two intercontinental populations. In the meantime, various degrees of stratification were observed in AMR populations by STRUCTURE analysis and presented as a mixture of a large amount of European ancestry component and a small amount of African ancestry component. Thus, the results also validated the well-known immigration history of the AMR population. The studied Eastern Chinese Han population was classified as an East Asian group based on their ancestry components and showed a closer genetic distance to Han Chinese in Beijing (CHB) than the Han Chinese in Southern China (CHS). In the drawing of the NJ tree, the placement of the five main populations generally follows as expected, in which the East Asian, South Asian and African populations were separated from other populations and the American population clustered with the European populations (Gandotra et al., 2020). These results indicated that our 163plex MH system could unambiguously differentiate these four major populations.

The polymorphism of utilized genetic markers is the theoretical basis of individual identification and paternity identification. Among the 163 MH markers, we screened 48 MHs with Ae ≥ 3.0 as the identification panel. The applicability of forensic identification was confirmed by statistical assessments. On the premise of HWE and LD compliance, the CPD of 48 MH loci was 1–8.26 × 10–44, higher than the CPD of 29 A-STR loci among the Chinese population (1–4.45 × 10–35) (Qu et al., 2021).

The CPED and CPET of the 48 MHs were 1–1.26 × 10–8 and 1–8.27 × 10–16, respectively. The simulated kinship analysis showed that the 48 MHs in this 163 MH assay could completely distinguish true and false father-child pairs, which could be used for basic paternity identification analysis. More persuasive data from the real father-child pairs and mother-child-father pairs will also be tested in the future.

For the mixture deconvolution study, 1 ng was used as the input quantity of the DNA sample, and simulated mixed sample cases with different mixing numbers and different mixing ratios were designed. The practical value of 45 MHs in mixture deconvolution was tested by comparing the sequence-based STR panel analysis results on the same NGS platform.

The application of NGS in STR detection can be used as a supplement to length-based STR (e.g., on capillary electrophoresis platform) in the analysis of mixture (Oldoni et al., 2020). NGS supports the simultaneous typing of many different STRs in a single reaction, thus enabling the recovery of the maximum information from a forensic sample. Furthermore, it can detect changes between STR sequences, which increases the discriminatory power of STRs (Gettings et al., 2015). For instance, the same alleles with different sequences could be distinguished based on the variation of specific sequences (isometric alleles). When the allele of the minor contributor coincided with the stutter position of the major contributor, it could be distinguished according to the difference in the sequence, facilitating the interpretation of the mixture. However, many minor contributor’s alleles would still be lost at the extreme mixing ratio due to the sensitivity of STR typing and the influence of stutter amplification (Magdalena et al., 2019), which was also confirmed in this study. Considering the rare situation of sequence variation, an exceptional minor contributor would be recognized only when the alleles were not within the stutter range of the primary contributor and had a certain coverage depth, which greatly reduced the availability of STRs in mixture analysis.

The stutter peaks of STR caused by polymerase slippage during the amplification makes the detection and deconvolution of an unbalanced mixture particularly complex, while MH does not produce a stutter peak. In the mixture study, we found that some of the MHs with higher level of polymorphism could improve the ability to analysis mixtures (Figures 3, 4). Our data demonstrated that the 45 MHs in the 163 MH assay we developed were effective in detecting and deconvoluting minor DNA in unbalanced mixtures. The successful interpretation of the simulated two-person and three-person mixtures and the mixed samples with high mixing ratios confirmed that this novel genetic marker had a more comprehensive deconvolution ability. The statistical results in this study also suggested that the detection system should be expanded for mixed samples with multiple participants, and MHs with higher Ae should be developed. Significantly, the practical application of MHs on the NGS platform may require a combination of theoretical construction and graph algorithms for a more efficient and accurate forensic practice solution.

## 5 Conclusion

In this study, an NGS multiplex system was constructed and demonstrated the ability to sequence 163 MH loci simultaneously. The system was verified by forensic validations with good accuracy, repeatability and sensitivity. Our results also showed that the data obtained by NGS were of high quality. The MH multiplex constructed in this study could therefore be suitable for forensic individual identification and paternity testing and could play an important role in the deconvolution of DNA mixture. Besides, this study provides an analytical method for assessing the ancestry inference ability of AIMs and suggests that screening more MHs with high In is necessary for refined population classification. Thus, the MH system seems to be a promising investigative detection method for forensic research and potential practical use.

## Data Availability

The original contributions presented in the study are included in the article/Supplementary Material further inquiries can be directed to the corresponding authors.
