# Supplementary material for: A sequence-based 163plex microhaplotype assay for forensic DNA analysis
Source: Front Genet. 2022 Oct 5;13:988223. doi: 10.3389/fgene.2022.988223 (PMC9579316; doi:10.3389/fgene.2022.988223)
Supplement: Supplementary file 1 [file Table1.DOCX]

Supplementary Figure 1. Uniformity of different concentration gradients. The left Y-axis represents the mean coverage of sample sequencing, and the right Y-axis represents the percentage of uniformity (>0.2*mean coverage ×).


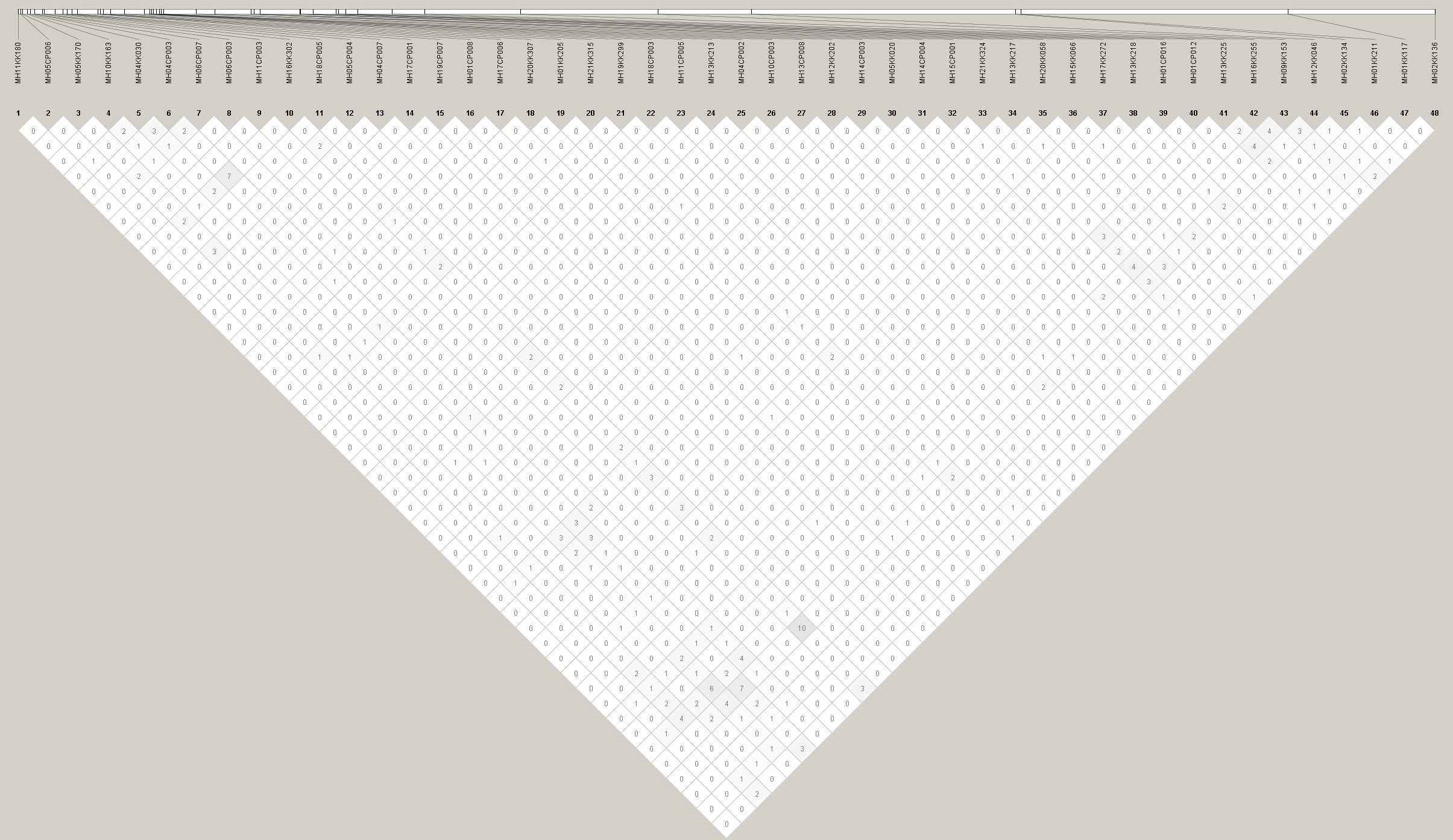


Supplementary Figure 2. Schematic diagram of linkage equilibrium of 48 MH loci.


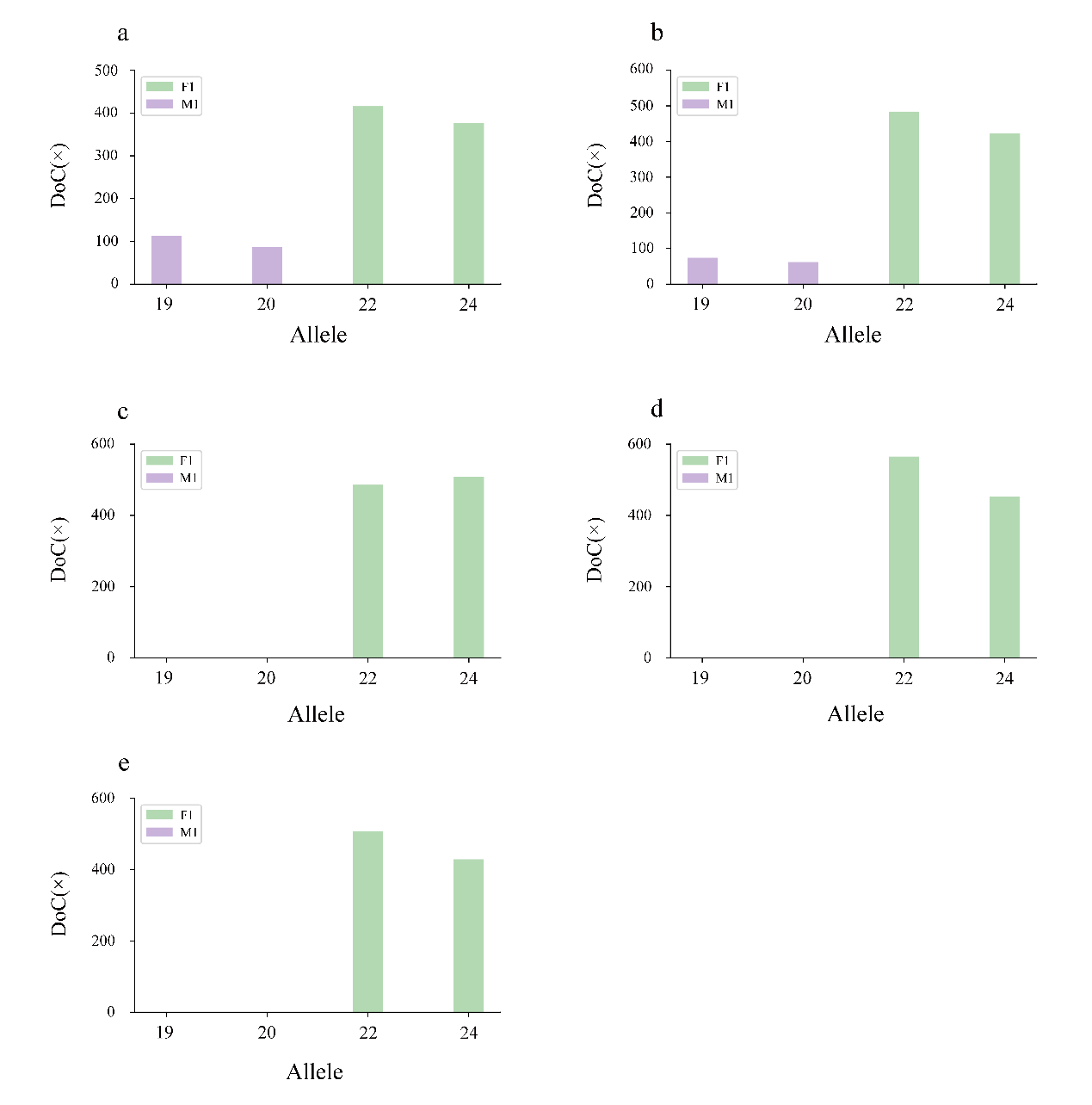


**Supplementary** **Figure 3.** Schematic diagram of two-person mixture at D2S1338 locus. The alleles of F1 were 22/24, the alleles of M1 were 19/20. (a) Mix ratio = 9:1, (b) Mix ratio = 19:1, (c) Mix ratio = 49:1, (d) Mix ratio = 99:1, (e) Mix ratio = 199:1.


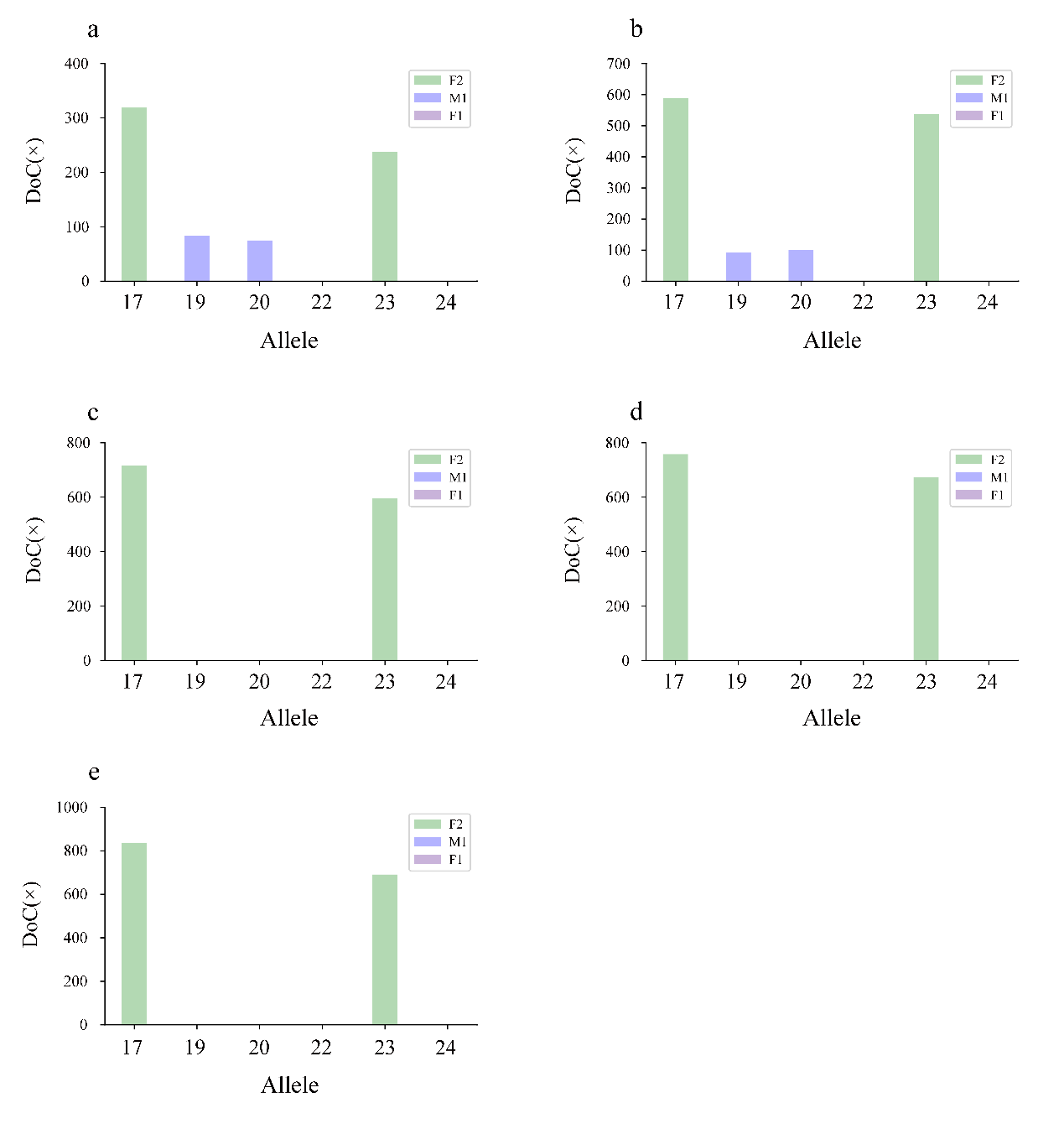


**Supplementary** **Figure 4.** Schematic diagram of three-person mixture at D2S1338 locus. The alleles of F2 were 17/23, the alleles of M1 were 19/20, the alleles of F1 were 22/24. (a) Mix ratio = 7:2:1, (b) Mix ratio = 17:2:1, (c) Mix ratio = 47:2:1, (d) Mix ratio = 97:2:1, (e) Mix ratio = 197:2:1.
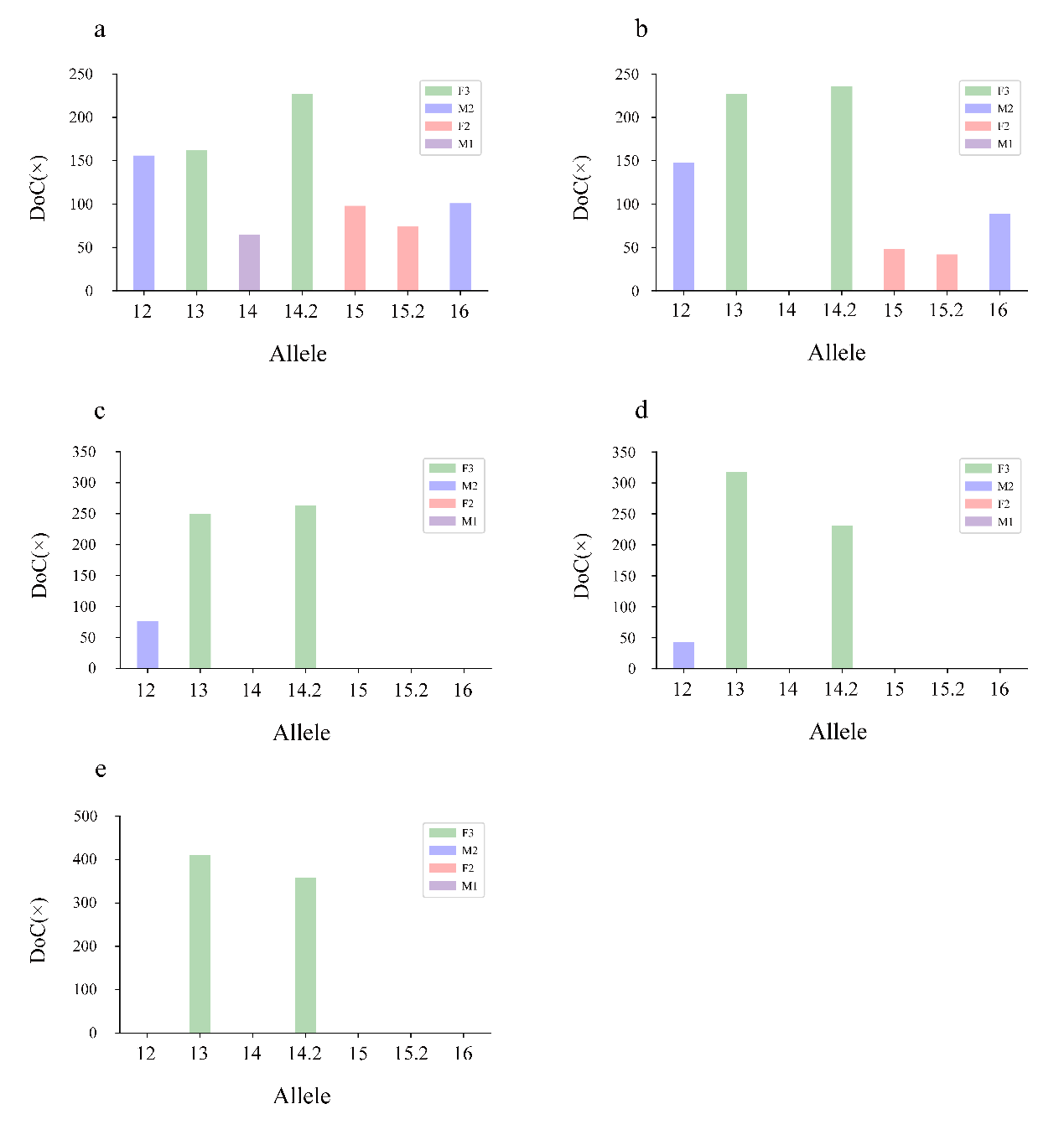


**Supplementary** **Figure 5.** Schematic diagram of four-person mixture at D19S433 locus. The alleles of F3 were 13/14.2, the alleles of M2 were 12/16, the alleles of F2 were 15/15.2, the alleles of M1 were 14/14.2. (a) Mix ratio = 5:2:2:1, (b) Mix ratio = 13:4:2:1, (c) Mix ratio = 43:4:2:1, (d) Mix ratio = 93:4:2:1, (e) Mix ratio = 193:4:2:1.


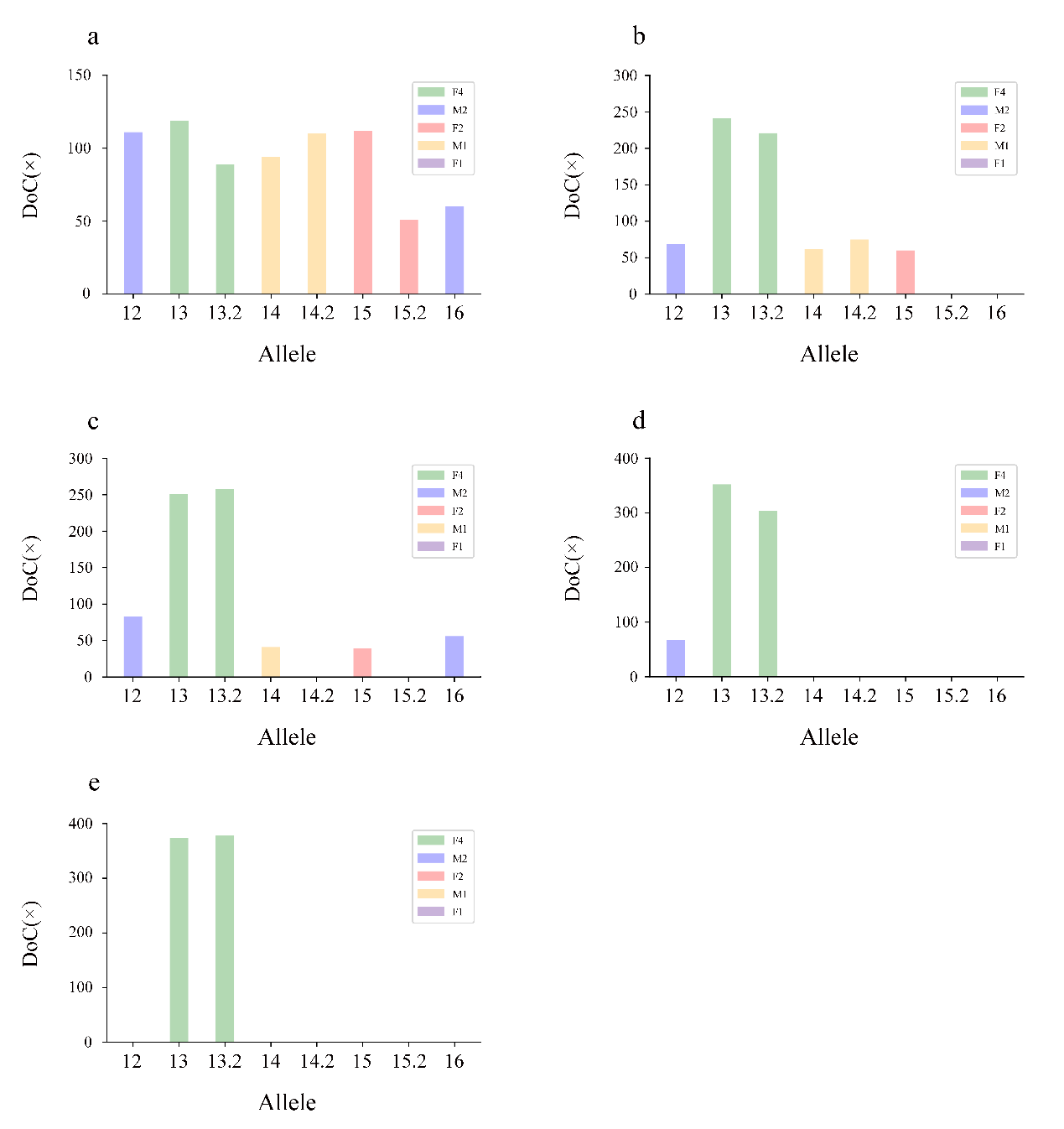


**Supplementary** **Figure 6.** Schematic diagram of five-person mixture at D19S433 locus. The alleles of F4 were 13/13.2, the alleles of M2 were 12/16, the alleles of F2 were 15/15.2, the alleles of M1 were 14/14.2, the alleles of F1 were 14.2/15. (a) Mix ratio = 3:2:2:2:1, (b) Mix ratio = 13:2:2:2:1, (c) Mix ratio =35:8:4:2:1, (d) Mix ratio = 85:8:4:2:1, (e) Mix ratio = 185:8:4:2:1.


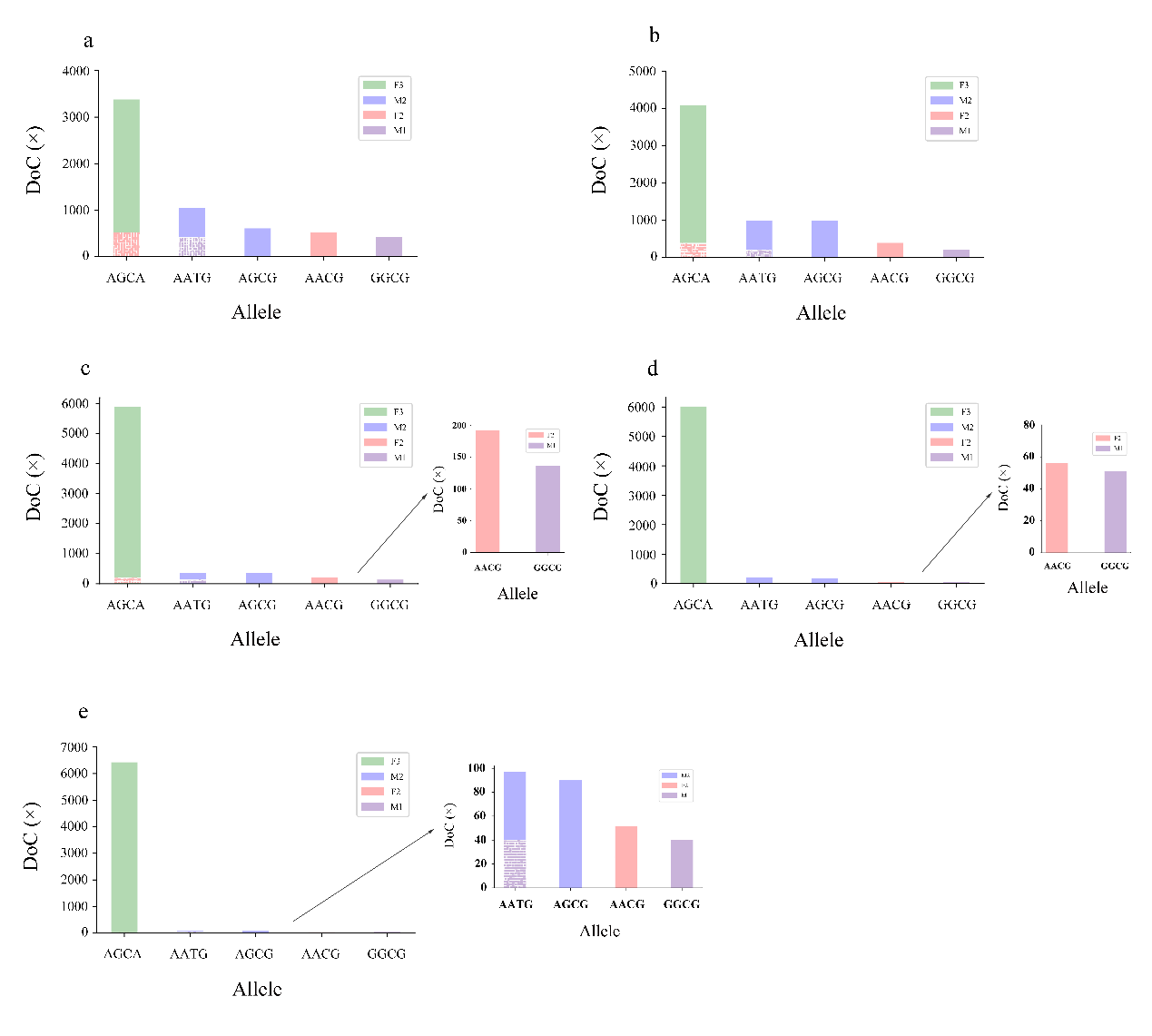
 **Supplementary** **Figure 7.** Schematic diagram of four-person mixture at MH13KK217 locus. The alleles of F3 were AGCA/AGCA, the alleles of M2 were AATG/AGCG, the alleles of F2 were AGCA/AACG, the alleles of M1 were AATG/GGCG. (a) Mix ratio = 5:2:2:1, (b) Mix ratio = 13:4:2:1, (c) Mix ratio = 43:4:2:1, (d) Mix ratio = 93:4:2:1, (e) Mix ratio = 193:4:2:1.


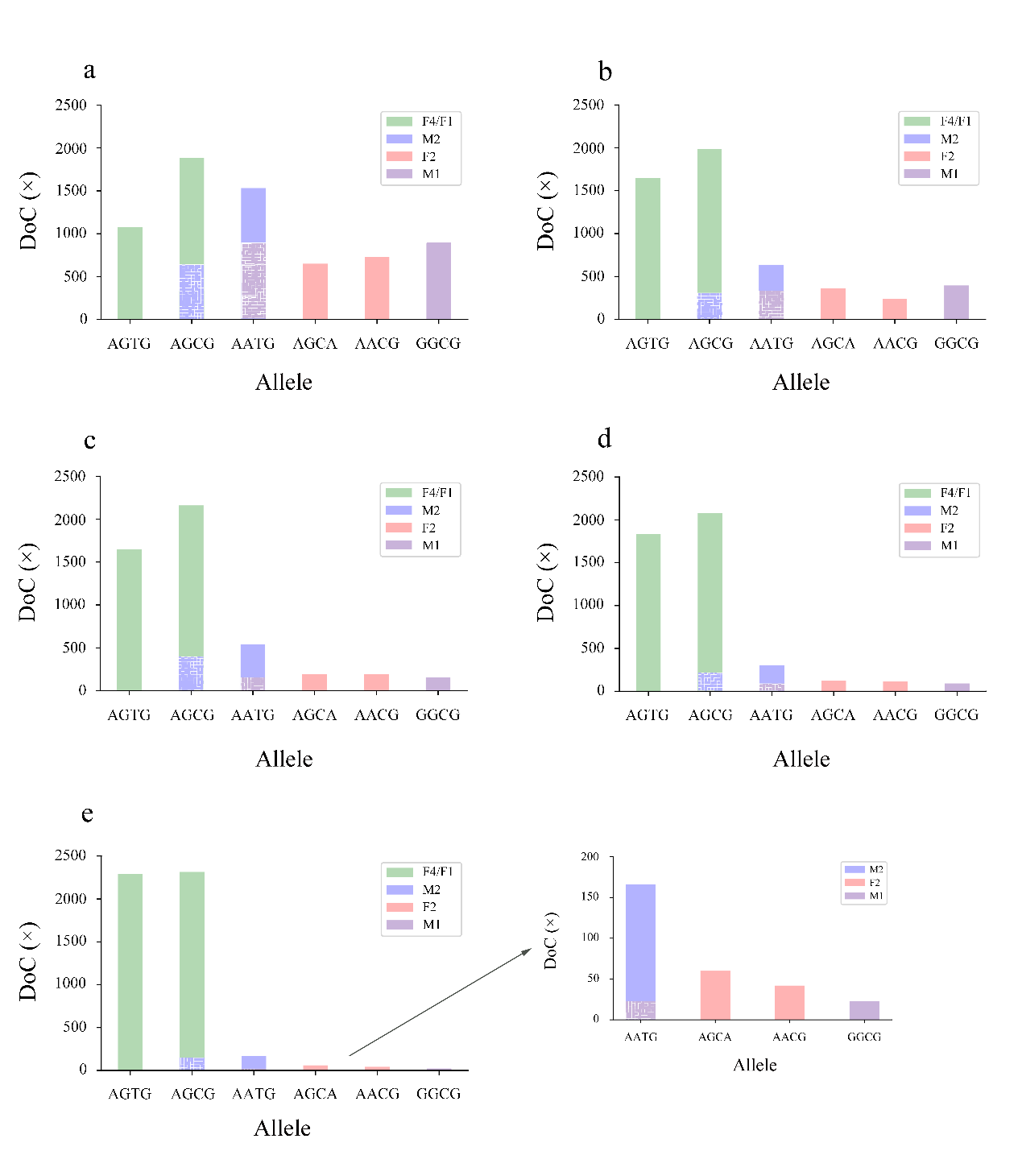


**Supplementary** **Figure 8.** Schematic diagram of five-person mixture at MH13KK217 locus. The alleles of F4 were AGTG/AGCG, the alleles of M2 were AGCG/AATG, the alleles of F2 were AGCA/AACG, the alleles of M1 were AATG/GGCG, the alleles of F1 were AGTG/AGCG. (a) Mix ratio = 3:2:2:2:1, (b) Mix ratio = 13:2:2:2:1, (c) Mix ratio =35:8:4:2:1, (d) Mix ratio = 85:8:4:2:1, (e) Mix ratio = 185:8:4:2:1.
