# Supplementary material for: A sequence-based 163plex microhaplotype assay for forensic DNA analysis
Source: Front Genet. 2022 Oct 5;13:988223. doi: 10.3389/fgene.2022.988223 (PMC9579316; doi:10.3389/fgene.2022.988223)
Supplement: Supplementary file 2 [file Table2.DOCX]

**Supplementary Table 1.** DNA mixture information.

| No. | Mixing ratio | Individual 1 | Individual 2 | Individual 3 | Individual 4 | Individual 5 |
| --- | --- | --- | --- | --- | --- | --- |
| A1 | 9:1 | F1 | M1 |  |  |  |
| A2 | 19:1 |  |  |  |  |  |
| A3 | 49:1 |  |  |  |  |  |
| A4 | 99:1 |  |  |  |  |  |
| A5 | 199:1 |  |  |  |  |  |
|  |  |  |  |  |  |  |
| B1 | 7:2:1 | F2 | M1 | F1 |  |  |
| B2 | 17:2:1 |  |  |  |  |  |
| B3 | 47:2:1 |  |  |  |  |  |
| B4 | 97:2:1 |  |  |  |  |  |
| B5 | 197:2:1 |  |  |  |  |  |
|  |  |  |  |  |  |  |
| C1 | 5:2:2:1 | F3 | M2 | F2 | M1 |  |
| C2 | 13:4:2:1 |  |  |  |  |  |
| C3 | 43:4:2:1 |  |  |  |  |  |
| C4 | 93:4:2:1 |  |  |  |  |  |
| C5 | 193:4:2:1 |  |  |  |  |  |
|  |  |  |  |  |  |  |
| D1 | 3:2:2:2:1 | F3 | M2 | F2 | M1 | F1 |
| D2 | 13:2:2:2:1 |  |  |  |  |  |
| D3 | 35:8:4:2:1 |  |  |  |  |  |
| D4 | 85:8:4:2:1 |  |  |  |  |  |
| D5 | 185:8:4:2:1 |  |  |  |  |  |

**Supplementary Table 2.** Information of 26 population samples from the 1000 Genome Project.

| Region | Abbreviation | Population | N |
| --- | --- | --- | --- |
| Europe | CEU | Residents of Utah, USA with Northern and Western European Ancestry | 99 |
|  | GBR | British in England and Scotland | 91 |
|  | FIN | Finnish in Finland | 99 |
|  | IBS | Iberian population in Spain | 107 |
|  | TSI | Toscani in Italy | 107 |
|  |  |  |  |
| North America | CLM | Colombians from Medellin, Colombia | 94 |
|  | MXL | Mexican ancestry from Los Angeles, USA | 64 |
|  |  |  |  |
| South America | PUR | Puerto ricans from Puerto Rico | 104 |
|  | PEL | Peruvians from Lima, Peru | 85 |
|  |  |  |  |
| Africa | ACB | African Caribbeans in Barbados | 96 |
|  | ASW | Americans of African ancestry in south-western USA | 61 |
|  | ESN | Esan in Nigeria | 99 |
|  | GWD | Gambian in Western Division in Gambia | 113 |
|  | MSL | Mende in Sierra Leone | 85 |
|  | LWK | luhya in webuye, Kenya | 99 |
|  | YRI | Yoruba in ibadan, Nigeria | 108 |
|  |  |  |  |
| South Asia | BEB | Bengali from Bangladesh | 86 |
|  | GIH | Gujarati Indian from Houston, Texas, USA | 103 |
|  | ITU | Indian Telugu from the UK | 102 |
|  | STU | Sri Lankan Tamil from the UK | 102 |
|  | PJL | Punjabi from Lahore, Pakistan | 96 |
|  |  |  |  |
| East Asia | CHB | Han Chinese in Beijing | 103 |
|  | CHS | Han Chinese in Southern China | 105 |
|  | CDX | Chinese Dai in Xishuangbanna | 93 |
|  | KHV | Kinh in Ho Chi Minh City Vietnam | 99 |
|  | JPT | Japanese in Tokyo, Japan | 104 |

**Supplementary Table 3.** Information of 163 MH loci.

| No. | MH | Chr | SNP |
| --- | --- | --- | --- |
| 1 | MH01CP007 | 1 | rs74887893/rs80137938/rs861907 |
| 2 | MH01CP008 | 1 | rs10803282/rs10803283/rs10927447 |
| 3 | MH01CP012 | 1 | rs12026749/rs1283256/rs8179472 |
| 4 | MH01CP016 | 1 | rs11206620/rs4927251/rs6684891 |
| 5 | MH01KK001 | 1 | rs4648344/rs58111155/rs6663840/rs6688969 |
| 6 | MH01KK070 | 1 | rs1801131/rs4846051 |
| 7 | MH01KK072 | 1 | rs1251078/rs1251079 |
| 8 | MH01KK106 | 1 | rs12123330/rs16840876/rs4468133/rs56212601 |
| 9 | MH01KK117 | 1 | rs1610400/rs1610401/rs17413714/rs2772234 |
| 10 | MH01KK172 | 1 | rs1887284/rs3128342/rs3766176 |
| 11 | MH01KK205 | 1 | rs11810587/rs1336130/rs1533622/rs1533623 |
| 12 | MH01KK210 | 1 | rs2165332/rs7536195 |
| 13 | MH01KK211 | 1 | rs16835127/rs2341465/rs2490423 |
| 14 | MH02CP004 | 2 | rs4668522/rs4669133/rs55990245 |
| 15 | MH02KK003 | 2 | rs11123719/rs11691107/rs260694 |
| 16 | MH02KK004 | 2 | rs13424991/rs3731611/rs3731612 |
| 17 | MH02KK073 | 2 | rs1374748/rs7583554 |
| 18 | MH02KK102 | 2 | rs2169812/rs2378217/rs6542783 |
| 19 | MH02KK105 | 2 | rs2280355/rs2280356 |
| 20 | MH02KK131 | 2 | rs1466020/rs17488897 |
| 21 | MH02KK134 | 2 | rs12469721/rs3101043/rs3111398/rs72623112 |
| 22 | MH02KK136 | 2 | rs12617010/rs6714835/rs6756898 |
| 23 | MH02KK138 | 2 | rs2595202/rs2595203/rs4953292/rs59298278/rs6715568/rs6759301 |
| 24 | MH02KK139 | 2 | rs12623957/rs3827760 |
| 25 | MH02KK201 | 2 | rs1371048/rs786247 |
| 26 | MH02KK202 | 2 | rs12464185/rs13422174 |
| 27 | MH02KK213 | 2 | rs1519654/rs7568519/rs7577785 |
| 28 | MH02KK215 | 2 | rs16832624/rs2011946 |
| 29 | MH03KK006 | 3 | rs1919550/rs9873644 |
| 30 | MH03KK007 | 3 | rs4513489/rs6441961 |
| 31 | MH03KK008 | 3 | rs17030627/rs6808142 |
| 32 | MH03KK009 | 3 | rs3732783/rs6280 |
| 33 | MH03KK216 | 3 | rs1046953/rs2072053 |
| 34 | MH04CP002 | 4 | rs34017818/rs35619595/rs6814654 |
| 35 | MH04CP003 | 4 | rs10006433/rs2980189/rs58595616 |
| 36 | MH04CP007 | 4 | rs4697751/rs4698039/rs4698040 |
| 37 | MH04KK010 | 4 | rs3135123/rs495367 |
| 38 | MH04KK011 | 4 | rs6531591/rs6855439 |
| 39 | MH04KK013 | 4 | rs11725922/rs13131164/rs17088476/rs3775866/rs3775867 |
| 40 | MH04KK015 | 4 | rs12648443/rs2584457 |
| 41 | MH04KK016 | 4 | rs2032350/rs2851017 |
| 42 | MH04KK017 | 4 | rs1442492/rs2584461/rs4699748 |
| 43 | MH04KK019 | 4 | rs17731793/rs2122136 |
| 44 | MH04KK028 | 4 | rs283413/rs3762896 |
| 45 | MH04KK029 | 4 | rs59534319/rs971074 |
| 46 | MH04KK030 | 4 | rs16844737/rs1884411/rs1884412/rs4916615 |
| 47 | MH04KK074 | 4 | rs11932595/rs17085763 |
| 48 | MH05CP004 | 5 | rs150628/rs16883189/rs61243436 |
| 49 | MH05CP006 | 5 | rs12653673/rs6555064/rs6555065 |
| 50 | MH05CP010 | 5 | rs62349578/rs62349579/rs62349580/rs62349581 |
| 51 | MH05KK020 | 5 | rs2278324/rs2278325/rs525735/rs617938 |
| 52 | MH05KK022 | 5 | rs41461/rs41462 |
| 53 | MH05KK062 | 5 | rs870347/rs870348 |
| 54 | MH05KK078 | 5 | rs2234233/rs2234234 |
| 55 | MH05KK079 | 5 | rs2234232/rs41469 |
| 56 | MH05KK122 | 5 | rs1010872/rs28777 |
| 57 | MH05KK123 | 5 | rs1423676/rs28117 |
| 58 | MH05KK124 | 5 | rs35414/rs3756464 |
| 59 | MH05KK170 | 5 | rs370672/rs438055/rs6555108/rs74865590 |
| 60 | MH06CP003 | 6 | rs12202010/rs4960100/rs4960101 |
| 61 | MH06CP007 | 6 | rs4142082/rs558006/rs6906397 |
| 62 | MH06KK026 | 6 | rs179939/rs4431439/rs4565296 |
| 63 | MH06KK030 | 6 | rs10949381/rs607341/rs675934 |
| 64 | MH06KK031 | 6 | rs10455681/rs10455682 |
| 65 | MH06KK080 | 6 | rs2056941/rs2056942 |
| 66 | MH06KK101 | 6 | rs2180052/rs9356632 |
| 67 | MH07KK030 | 7 | rs10226425/rs2330425/rs967066 |
| 68 | MH07KK031 | 7 | rs10246622/rs17168174 |
| 69 | MH07KK081 | 7 | rs28365094/rs41303343 |
| 70 | MH07KK082 | 7 | rs150209521/rs713598 |
| 71 | MH08KK032 | 8 | rs1390950/rs2898295 |
| 72 | MH09KK020 | 9 | rs10810635/rs10962598/rs10962599/rs73649032 |
| 73 | MH09KK033 | 9 | rs10815466/rs17431629/rs9408671 |
| 74 | MH09KK034 | 9 | rs1408800/rs1408801 |
| 75 | MH09KK152 | 9 | rs10780576/rs10867949/rs4282648/rs7046769 |
| 76 | MH09KK153 | 9 | rs10125791/rs2987741/rs7047561 |
| 77 | MH09KK157 | 9 | rs2073578/rs56256724/rs606141/rs633153/rs8193001 |
| 78 | MH09KK161 | 9 | rs16932430/rs4741823 |
| 79 | MH10CP003 | 10 | rs10764460/rs220365/rs727269 |
| 80 | MH10KK083 | 10 | rs11568732/rs12248560 |
| 81 | MH10KK084 | 10 | rs1058930/rs11572103 |
| 82 | MH10KK085 | 10 | rs11572076/rs2275622 |
| 83 | MH10KK086 | 10 | rs17110453/rs7909236 |
| 84 | MH10KK087 | 10 | rs10884095/rs1452267 |
| 85 | MH10KK088 | 10 | rs2515641/rs55897648 |
| 86 | MH10KK101 | 10 | rs915907/rs915908 |
| 87 | MH10KK163 | 10 | rs3814588/rs3814589/rs3814590/rs6602026/rs9423466 |
| 88 | MH10KK170 | 10 | rs12359688/rs2250840/rs2250841 |
| 89 | MH11CP003 | 11 | rs12289831/rs2045045/rs2045046 |
| 90 | MH11CP004 | 11 | rs35728001/rs76882177/rs77516091 |
| 91 | MH11CP005 | 11 | rs7118419/rs72865222/rs7926642 |
| 92 | MH11KK036 | 11 | rs10500616/rs2499936 |
| 93 | MH11KK037 | 11 | rs10898849/rs341065/rs395447 |
| 94 | MH11KK038 | 11 | rs2303377/rs2303378 |
| 95 | MH11KK039 | 11 | rs10891537/rs2288159 |
| 96 | MH11KK040 | 11 | rs11214596/rs4938013 |
| 97 | MH11KK041 | 11 | rs6275/rs6277 |
| 98 | MH11KK089 | 11 | rs1124492/rs1124493 |
| 99 | MH11KK090 | 11 | rs1079597/rs1079598 |
| 100 | MH11KK091 | 11 | rs1799732/rs1799978 |
| 101 | MH11KK180 | 11 | rs12802112/rs28631755/rs4752777/rs7112918 |
| 102 | MH11KK187 | 11 | rs17137917/rs17137926/rs493442/rs551850 |
| 103 | MH11KK191 | 11 | rs12289401/rs12420819/rs12421109/rs770566 |
| 104 | MH12KK042 | 12 | rs593226/rs7969300 |
| 105 | MH12KK043 | 12 | rs11062734/rs11613749/rs17780102 |
| 106 | MH12KK045 | 12 | rs2133298/rs3817446 |
| 107 | MH12KK046 | 12 | rs11068953/rs1503767 |
| 108 | MH12KK092 | 12 | rs2707209/rs2857234 |
| 109 | MH12KK093 | 12 | rs11111391/rs7970874 |
| 110 | MH12KK202 | 12 | rs10506052/rs10506053/rs4931233/rs4931234 |
| 111 | MH13CP008 | 13 | rs9507311/rs9553248/rs9553249 |
| 112 | MH13KK047 | 13 | rs2066700/rs806301 |
| 113 | MH13KK213 | 13 | rs679482/rs8181845/rs9510616 |
| 114 | MH13KK217 | 13 | rs2765614/rs7320507/rs9562648/rs9562649 |
| 115 | MH13KK218 | 13 | rs1927847/rs7492234/rs9536429/rs9536430 |
| 116 | MH13KK225 | 13 | rs4884651/rs7329287/rs9529023 |
| 117 | MH13KK226 | 13 | rs2892698/rs721367 |
| 118 | MH14CP003 | 14 | rs12436504/rs66481544/rs7155003 |
| 119 | MH14CP004 | 14 | rs11157032/rs11157033/rs11157034 |
| 120 | MH14KK048 | 14 | rs12717560/rs12878166 |
| 121 | MH14KK101 | 14 | rs10134526/rs28529526 |
| 122 | MH15CP001 | 15 | rs12899727/rs34090207/rs369577479 |
| 123 | MH15CP003 | 15 | rs12440416/rs578662/rs58022506 |
| 124 | MH15CP004 | 15 | rs28628574/rs34306395/rs506120 |
| 125 | MH15KK066 | 15 | rs1063902/rs4219 |
| 126 | MH15KK067 | 15 | rs701463/rs701464 |
| 127 | MH15KK069 | 15 | rs1800410/rs1900758 |
| 128 | MH15KK095 | 15 | rs2433354/rs2459391 |
| 129 | MH16KK053 | 16 | rs11150606/rs201075024 |
| 130 | MH16KK062 | 16 | rs28485311/rs28503604/rs8055777 |
| 131 | MH16KK096 | 16 | rs1805007/rs885479 |
| 132 | MH16KK255 | 16 | rs16956011/rs3934955/rs3934956/rs4073828 |
| 133 | MH16KK302 | 16 | rs1395579/rs1395580/rs1395582/rs9939248 |
| 134 | MH17CP001 | 17 | rs36040276/rs4792125/rs62063465 |
| 135 | MH17CP006 | 17 | rs2215237/rs62069897/rs9897281 |
| 136 | MH17KK014 | 17 | rs11657785/rs333113/rs8074965 |
| 137 | MH17KK052 | 17 | rs1059504/rs8327 |
| 138 | MH17KK053 | 17 | rs3760370/rs3760371 |
| 139 | MH17KK054 | 17 | rs2233362/rs634370 |
| 140 | MH17KK055 | 17 | rs11868709/rs9907137 |
| 141 | MH17KK077 | 17 | rs4074461/rs4074462 |
| 142 | MH17KK105 | 17 | rs1052553/rs11568305/rs17652121 |
| 143 | MH17KK110 | 17 | rs8075367/rs9908046 |
| 144 | MH17KK272 | 17 | rs16955257/rs2934897/rs7207239/rs7212184 |
| 145 | MH18CP003 | 18 | rs12970683/rs58533252/rs78549053 |
| 146 | MH18CP005 | 18 | rs595107/rs62085085/rs690302/rs77849214 |
| 147 | MH18KK285 | 18 | rs16940823/rs17187688/rs17187695/rs1945150 |
| 148 | MH18KK293 | 18 | rs621320/rs621340/rs621766/rs678179 |
| 149 | MH19CP007 | 19 | rs10417429/rs10417450/rs34190726 |
| 150 | MH19KK056 | 19 | rs1055919/rs2271057 |
| 151 | MH19KK057 | 19 | rs12462026/rs17717333/rs7250849 |
| 152 | MH19KK299 | 19 | rs12985452/rs2361019/rs2860462/rs4932769/rs4932999 |
| 153 | MH19KK301 | 19 | rs10408037/rs10408594/rs11084040/rs8104441 |
| 154 | MH20KK058 | 20 | rs6012881/rs6095836/rs6122890 |
| 155 | MH20KK059 | 20 | rs10854214/rs10854215 |
| 156 | MH20KK307 | 20 | rs16997830/rs17674942/rs6044080/rs6044081 |
| 157 | MH21KK313 | 21 | rs6586324/rs6586325/rs6586326 |
| 158 | MH21KK315 | 21 | rs6517971/rs8126597/rs8131148 |
| 159 | MH21KK316 | 21 | rs17002090/rs2830208/rs961301/rs961302 |
| 160 | MH21KK324 | 21 | rs2838868/rs6518223/rs7279250/rs8133697 |
| 161 | MH22KK060 | 22 | rs4680/rs4818 |
| 162 | MH22KK064 | 22 | rs136177/rs60910145/rs71785313/rs73885319 |
| 163 | MH22KK303 | 22 | rs4633/rs6267/rs740602/rs76452330 |

**Supplementary Table 4.** Primers of 163 MH locus.

| No. | MH | Primers |
| --- | --- | --- |
| 1 | MH01CP007 | TTCTCCCCAAATCACAGCACCC |
|  |  | CGTAAGGATGGGCAAAACGTTCA |
| 2 | MH01CP008 | AAGCAGTTTGATGTGAGCTCTAAAACGA |
|  |  | GCCAGTAGAAATTCTAAAACAAAACCCA |
| 3 | MH01CP012 | ATCATTTTCTCAGTGCGCAACAC |
|  |  | CTTTGATGTCAGATTTTCTTAGGACCGA |
| 4 | MH01CP016 | CACTCACTTTGTGACCATTCCGGT |
|  |  | CTGAAGGACTACTACCTCTTCTACCT |
| 5 | MH01KK001 | GATGAGCACCTCGAGAAGACCT |
|  |  | GATGGCTGGTACCGATCATCTC |
| 6 | MH01KK070 | TAGCAACGCCAATCTCAGAGAGGT |
|  |  | TGCTGTAAGCACTCTACACATATCAATT |
| 7 | MH01KK072 | ATAAGCTATGCTGAGGGAAGTCTGG |
|  |  | ATGAAGCTGGCTCAGTCAACTC |
| 8 | MH01KK106 | CATAGTTTCCAGAGTGGTTTGCAGGC |
|  |  | ATGAGATGGGTGGTGGACAGTTA |
| 9 | MH01KK117 | TCCTAGGCGTAAATGGATGAGAG |
|  |  | ATGATAGAATGTAGAACCCAGCCATC |
| 10 | MH01KK172 | CTTAATGATAATGCTGGCAGAGTCTG |
|  |  | TTGATATATTTCCAAACACCTGTGTGCT |
| 11 | MH01KK205 | ATCTTTAAGAGTCCGCTTTGTGTTT |
|  |  | AATGTCTCCCTGAGGAATTCTACCT |
| 12 | MH01KK210 | GCAAGATACCAAGTTCTTGAATAAGGAG |
|  |  | CACCTCCTCCATAATCCACAAGTG |
| 13 | MH01KK211 | CACAAAATGAGAGGAAGGTTACTGAG |
|  |  | CAAAGGAGGTCACATCACCATCTC |
| 14 | MH02CP004 | GAATCTACTTCACTTGAATGCATGTTAA |
|  |  | GGAGAAACTAAGCCATATATCCATGGT |
| 15 | MH02KK003 | TCAATCACCATGTTTTGACTCAGTTTA |
|  |  | AATTCCCTCAGAGAGATTATTCGATGC |
| 16 | MH02KK004 | GATTGTTCTATGATGCTGGGTAGGGGG |
|  |  | TGTGTTCAGGATACCATGCCATTAG |
| 17 | MH02KK073 | AGGAAGGCTAATGACCTCGCAAT |
|  |  | GACACCACCAGAACTTCTTGCTTATTA |
| 18 | MH02KK102 | TCTCACTTATGATGCTGCTAGACTGAC |
|  |  | AAGAGCACATGAGATCCGCAATC |
| 19 | MH02KK105 | GGAGCTTGCTAGAGAAGATCACGG |
|  |  | ATTGCTCAGCCACAAAAGATTCTCA |
| 20 | MH02KK131 | TTTAATAGTGAAAGCAGCAAGGTTCTTCA |
|  |  | TTTTCCCAGATAAATTTCAGTGTCAGCT |
| 21 | MH02KK134 | AAAGAGTTGCATGCCGTCTGT |
|  |  | GTTCTAGGTGTCGTTTGCCTTAAGTTA |
| 22 | MH02KK136 | AGTTCTCAAAGACTTCAAGACAAGTTA |
|  |  | TCTTTTCTCCACTTTTCAGACTTCTTGT |
| 23 | MH02KK138 | ACCATCTCAGTGCTGAAAGAAATATAAA |
|  |  | CCAGACTCATCACGTCATCCAGATA |
| 24 | MH02KK139 | GTGACAGCTAGGTTTCATTACTGCG |
|  |  | AAGCCAGGATTTACCCATTTATGGAG |
| 25 | MH02KK201 | CCAAGCTCCCTGTGATATTTCTAAA |
|  |  | ACTGGAAGAGTCTTTTGTTTCATAGCC |
| 26 | MH02KK202 | GCCTTTTCCCCTTATTCTTTAAACAA |
|  |  | TGTTATCTCACCACTCACACATTAACTT |
| 27 | MH02KK213 | CTCAGTAGTGAACTGCCTCAGGG |
|  |  | CCTTCCCCAACACTCTCTAAATATTTGC |
| 28 | MH02KK215 | ATGCAACACTGCACCTGAGAATATG |
|  |  | TACCCCCTAAAAGGTTTTGAATGCAG |
| 29 | MH03KK006 | AACCAACTAATCTACTGAAGGACTGG |
|  |  | CAAGAGGGACACCATATGTCAAGG |
| 30 | MH03KK007 | CATTTTTGAAGGCTCCCATATTGCAT |
|  |  | AAATGTGCAGAAAGATTCCAAAGGAG |
| 31 | MH03KK008 | AGGTACCCATCAACCTCTTTGTT |
|  |  | ACCTATGTGGCTGTACAATTTGTCC |
| 32 | MH03KK009 | GAAGTCTACTCACCTCCAGGTATACC |
|  |  | CCAAGCCCCAAAGAGTCTGATTTTAT |
| 33 | MH03KK216 | AAGAGCTGAAACAAGAGCATTGTGCA |
|  |  | CCACATTGTAACTCCTAGACCAAGAAG |
| 34 | MH04CP002 | ACACAGAGTTTAAGGTTCCTTCCAGAA |
|  |  | GGGTCACTTCAGGATAATAAGCTCCT |
| 35 | MH04CP003 | GATTTGTGTCTTCTGCATTCACAGCT |
|  |  | GGCTGCTCTTGTACAGCATCTC |
| 36 | MH04CP007 | TAAATACTGTCTGCCCATGACTCCTC |
|  |  | AGAGCTTTGGTTTTAATGCTATTCCCT |
| 37 | MH04KK010 | TCACTATATTTTTGAGGACACCAACCAC |
|  |  | TGTTGGTGCCAAGTACATCTATAAGAA |
| 38 | MH04KK011 | TTTTAAGAAAGAATAAAGAAGGACAGAAAGCCA |
|  |  | GATCATGCTATCACTAAGAAAATTATGGCAAA |
| 39 | MH04KK013 | TGTCTAATGGCCGCTGTAGTAAA |
|  |  | CTTGGCAATTTAAGATGCTCAGGAATT |
| 40 | MH04KK015 | AATTCTATCTCATCCATCTTGAGTGCAT |
|  |  | TATTACAGAGTGCTGCAGGTCATTC |
| 41 | MH04KK016 | CAAAGCTAGTTTCTAAGTAAGCCATTGC |
|  |  | TTTTTGCCAGAGTTTTTAGTGTACTCCT |
| 42 | MH04KK017 | ATAATGGTTGAAGGGTAGAATACACGCA |
|  |  | TCGTTCAGATGAGCATGTGGTTAG |
| 43 | MH04KK019 | TACTTGTAGCAGAGGGCCTTATC |
|  |  | GTTAGACAGAAGTTAGGCATGGAGTT |
| 44 | MH04KK028 | TAATGGAAGTACTGTTTCAGTTCTGCAA |
|  |  | AAAAATGTTTTCCTTTTCTTCCTAGGGC |
| 45 | MH04KK029 | CATTTACCAATGTTGGCTAATACACA |
|  |  | AGAACAGCATAGGAAGGCACTTAG |
| 46 | MH04KK030 | AAATTTTGGGTCTTACCATGGTTTCAA |
|  |  | TTGTGTTTTTAACTGGAGGCCCTT |
| 47 | MH04KK074 | ATATTTAAACAAAGGCTCTGGGTGTAA |
|  |  | CAGGGACTTCTCTAGTTTCATGTGT |
| 48 | MH05CP004 | TGGGAACAAAGTCTCGGATGTACT |
|  |  | CAGCAGGACATTGACAGATACTCATTAT |
| 49 | MH05CP006 | AGAAAAATGGCAGAGACCTTGACAC |
|  |  | TCTACTTTCTGTTCTCTTTGTGTTTCCG |
| 50 | MH05CP010 | CAATCACATTGTTCCCTAGTGTCTC |
|  |  | AGGTGACATTGACAGAGTTGCAAATA |
| 51 | MH05KK020 | AATAAATCGCAATGGAAGCAACAGGAA |
|  |  | CTCCTAGGGCTTGTGAGTCTCATA |
| 52 | MH05KK022 | GTTGCCAATCTTACCACACCTCCA |
|  |  | AGCCTTTTTCTTAGGACCTGACATAG |
| 53 | MH05KK062 | TGAACTGATCCAACTTCTCTCTCACTG |
|  |  | CTCAGTGCCATTGCTTATCTTCCTT |
| 54 | MH05KK078 | CAACAAAAGAGAAAATCTGTATAGCCAG |
|  |  | TTTCTGCAGTTGTTCATCTTCTACGTTA |
| 55 | MH05KK079 | TTATTGGTCTGCTCAGAGTTTACATCAG |
|  |  | ACAGAACATTCTACCCAAGATTCTATGC |
| 56 | MH05KK122 | CAACATTTTTCATGTGGCCCCTACT |
|  |  | GGAACAAAACAAGGTGCGGTTTT |
| 57 | MH05KK123 | AGTGTTCTGCCAGGGTCAAAATAA |
|  |  | ATTGAATGCCAAAACCTCAGGGATA |
| 58 | MH05KK124 | CAGACAAGCTGATCTGATATTTCTTTAG |
|  |  | GCCGCCTAAGGGATTTACCAATATG |
| 59 | MH05KK170 | AAGACCTGAGTAGCTTCTGTTTTCTC |
|  |  | GGTGCTGTAATTCCCCTAAAAGCAA |
| 60 | MH06CP003 | CAAGGAATAAAGCAGTGTGTGCCT |
|  |  | CCTCAAGAATCCTGGAAAATGTCAGC |
| 61 | MH06CP007 | ACACTATTTTAAATTAGTCAACAGTTAAGCATA |
|  |  | CTGAAACATCACTCAAAATAAAAGGCATT |
| 62 | MH06KK026 | TCTACAACTAAGCCTTTTAACCGAGA |
|  |  | ATTTCACAGTTCTCTCTTGATCATGTCA |
| 63 | MH06KK030 | GAATGCACAGAGAAATTCTTAGAGGTCA |
|  |  | CTCCACCTCTTGTCTTCTAGAACCAT |
| 64 | MH06KK031 | TCTTTGTATTCACTATTCTTGTGGCTAA |
|  |  | TTTCAAGATGGGATGGAGAAAGCTA |
| 65 | MH06KK080 | CCCTATTCCAAACCTGTACCTACCT |
|  |  | CCCCAGTCACCCACCTAACATTTAAT |
| 66 | MH06KK101 | GAGCCTGAGACTCTGCTACCA |
|  |  | GGGAGTCCCACGAGCACTG |
| 67 | MH07KK030 | AAGTGTAGTCTGTGCAACAAGTTTCTTA |
|  |  | ATACAAGGATTTAGAGACCACAGCATC |
| 68 | MH07KK031 | CTTTGGAGAAAACTGATGAGTTTAGCTT |
|  |  | CCTCTGTCTTCTTAACTGGCTGTAG |
| 69 | MH07KK081 | TAAGTTGGAATCACCACCATTGACCC |
|  |  | ATTCATAACTCCTCCACACATCTCAGTA |
| 70 | MH07KK082 | TGAGCTTGGAGCAGTAAAGCAGG |
|  |  | AGTGACATCATGTTGACTCTAACTCG |
| 71 | MH08KK032 | AACTTGTTGCAGATTCATGGAATCATTT |
|  |  | AAAGAGAATAACAGTTTGACCTTGGC |
| 72 | MH09KK020 | ATGACAGAAGAGATTTCTCTCCAGTTTG |
|  |  | ACTCGATTCTTTCCATTTCCATGTCG |
| 73 | MH09KK033 | TTAAAGTCTCCTGTGTACACGGTTG |
|  |  | CTGTACCAATCAAGAGAAGTAGGATGGA |
| 74 | MH09KK034 | GATATTTGTAAGGTATTCTGGCCTAAAAAA |
|  |  | AAGTATTGAAGTGATAGTTTTACAGTTTCCT |
| 75 | MH09KK152 | AGACTTGGAATCATTCTTCACAGGGT |
|  |  | GCCAGAATTAGCAGTTAGCAGTCAT |
| 76 | MH09KK153 | TTTCTTCCTCTAAGTGGCCTCATAAATA |
|  |  | AGAATTAGTAAGCTCTTTCACTTGCAGT |
| 77 | MH09KK157 | ACTAGAAGCATTAGACCAGATTACCTGC |
|  |  | ACAGTCCATTAGTGATGGGTTTGTT |
| 78 | MH09KK161 | CAGAAAAACAGACTGGTCCAAAGTC |
|  |  | CACTGGTTTGGGAATAGAGTGCTAAG |
| 79 | MH10CP003 | CCCCCAGAAAAGTATGTTTTAAGACTCT |
|  |  | CCAAGACCAGAGAGATAACAAATGCAA |
| 80 | MH10KK083 | TTTCTGAATGTGGCCTACAGTTTCAC |
|  |  | ATGGAATTCGAAATGATGAAGCAATGA |
| 81 | MH10KK084 | TGTTGCTTATGCTGTTGTTCTTCACCC |
|  |  | GTTTGTACTTCTTTAAAGCAGGGACTG |
| 82 | MH10KK085 | GGAGGTCAAGAAGCCTTAGTTTCTC |
|  |  | ATCGTGGCGCATTATCTCTTACATC |
| 83 | MH10KK086 | GCATTCTAGCCATTGGACAATTTTGTA |
|  |  | TAGGTCTGCAATAATTTCCCTCTACTCA |
| 84 | MH10KK087 | ACTGTTAAGGTCAATGACGCAGAGTA |
|  |  | TTACTAAAGGACTTGGTAGGTGCACATA |
| 85 | MH10KK088 | TTTGGCCCATGGATAGAAATAAAATGTT |
|  |  | TTTGAAAGGCTTTTGTTATCAAGGGCTA |
| 86 | MH10KK101 | CCATTCCCTATTCAGTGGACTCTT |
|  |  | AGACTCAGTGAGGTCATGACTCAA |
| 87 | MH10KK163 | GAGCATCTTCTCCACCAGTTTGGC |
|  |  | TTGTCTCCTTTCAGCACAGAACC |
| 88 | MH10KK170 | AAAGCCCACATTTTGTTAACATGACTC |
|  |  | ATGTAACTTCTCTGAACAGGGAAGAG |
| 89 | MH11CP003 | AAGCAGCGATTTCCATGTTGCCC |
|  |  | GGCTGATTGTGGAGATGTCTCCT |
| 90 | MH11CP004 | AGAAGCCAAAGCTCCCTAATAGCTC |
|  |  | GAGCCAGTTTTGTTAAAGACACAATGT |
| 91 | MH11CP005 | TTGCTCTGAATAGTGCTTTCAGTAGTG |
|  |  | CAGCACTTTCTAAATAGTGATAGGCAAG |
| 92 | MH11KK036 | CAGCTGCTTATAGTTTTGTTAAGAAG |
|  |  | GGACCCCTAGATAATGTCAGGATTG |
| 93 | MH11KK037 | CTTTTGAGATCATGGAAAATTCCAGTTG |
|  |  | CAGAAAGAGGAACTTAAGAAGATGTGGT |
| 94 | MH11KK038 | GGAGTTCTAAGCAATGAGATGCTAATT |
|  |  | TTTCCCATAATTCCCAAAGCATGGTA |
| 95 | MH11KK039 | AGCATCATTTCATGCTTTTGAAGTTT |
|  |  | ACCACCTCCTGTAACAACATCCG |
| 96 | MH11KK040 | AGAACCCATAGGGAAACAAAGGTATGT |
|  |  | TTTCTCTCCTTTCAGGGAACATTACATC |
| 97 | MH11KK041 | CATTCAGTATCTGTGTGCCTCAATGAT |
|  |  | CTGCAGGGTTTTCTATCCAGAACAAT |
| 98 | MH11KK089 | CAGAATGATGAGCTGTGCAGATAGCC |
|  |  | GCTGTCTCTATGAACATCCCTACC |
| 99 | MH11KK090 | TGTGATGGAGTTTATGGCCAACGG |
|  |  | TTATGCCCCAAATTTCACTGCTTAG |
| 100 | MH11KK091 | AACTCCGGTCTATCCAGGTCC |
|  |  | TGATCCCATGGGACTACTCACG |
| 101 | MH11KK180 | GCATCTGAGTGGCTTTCTTCTCC |
|  |  | CTGGGAACTTGTCCGGCTTTA |
| 102 | MH11KK187 | TAGGAGTTTATACATGATCCTAAGGGCA |
|  |  | ATTTTTGGCCAAACAGAATTGTTTGC |
| 103 | MH11KK191 | CACCAAAGGAGCTGTACCTCC |
|  |  | GTCAACTCCAAACAGGCTTTTTCC |
| 104 | MH12KK042 | TTGCAAACTATGTCAAGGACACATTT |
|  |  | GCAAATGATCTCAGAGTTGCACAAATTG |
| 105 | MH12KK043 | GATGAACAGCTTGGATTGGGGC |
|  |  | CAGCTGAGACATAGAGAGAGGACTT |
| 106 | MH12KK045 | AACAGGTCATGGAAGCTTTAGATCTT |
|  |  | AAAATCCTGGTGATAAACGTACAACCT |
| 107 | MH12KK046 | TGTCAGCTTCTTGCGTGATAGTG |
|  |  | TTTTTCCCCAAGAGTCTCATCTATTAGC |
| 108 | MH12KK092 | CATGTCTCCTTCCCTTGGTTATACC |
|  |  | AAAAATTGCAAGAGCAATAAGCATGTG |
| 109 | MH12KK093 | ATCTTTTGCCTTGGCATTTGGTCTG |
|  |  | CTAGTTTGCTTCCTTCTATGACCCCTA |
| 110 | MH12KK202 | GAGAGAGTGAACAGATGAGAATCAGAAA |
|  |  | TTGTAATGGCCTTGGGATCAAATATTCT |
| 111 | MH13CP008 | AGAGCTTTAGTAAGACCTCAGACTG |
|  |  | TAAACCAGACTGAATGTCAAAGACAAAC |
| 112 | MH13KK047 | GAATAACCAGTACCAGGCACGGC |
|  |  | TCCATCCCTTTGAGTCTATGTGTCC |
| 113 | MH13KK213 | CTCTTGCTTCTGTCAGACACTTTTAATT |
|  |  | CGAGTCTCTTTTTGGTGTATTGCCA |
| 114 | MH13KK217 | CTGGGAAACCAGCTAGAAGAAGAGA |
|  |  | CAAACGCACTGAGCTATTTACCTTAG |
| 115 | MH13KK218 | GCCTCCCTTTCAGATCTTACTTAGGT |
|  |  | AAAATGCAACACACCTAATACTTCAGT |
| 116 | MH13KK225 | ATGTCAGGATGCTCCACAACGGT |
|  |  | TCCACAGAGCATCAGCTATGAATC |
| 117 | MH13KK226 | CTGATCTTACAAGTTCACGGCTTGT |
|  |  | TTCTCTATATGACCAGCCTCTTTACATG |
| 118 | MH14CP003 | GCTGGGCATATACTCCAAAGACAG |
|  |  | CCAGTCTCTAGTAACTGTCCTTCTCTG |
| 119 | MH14CP004 | GATATTAGCCCTTTGCCAGATAGATAGGTT |
|  |  | GGGAAAGGATTCCCTATTTAATAAATAGTGTC |
| 120 | MH14KK048 | TGTCTGGAAAACTGTAGCGTGT |
|  |  | CCATGCACAATTAGGAACAACAGTG |
| 121 | MH14KK101 | GATGCGGGATAAGGAATTAATCAAGGAA |
|  |  | CACTATGCCTAGCTTTGTCTTGTCTTA |
| 122 | MH15CP001 | GTACTGCAGTCACACAAAGCAGA |
|  |  | CTAATGAAAGGCTGCCTCTGTTCT |
| 123 | MH15CP003 | CACACGTGCTAGTTAGGCTAAATA |
|  |  | CTTCCTTTGTGACTTCTGTTGCATTTAT |
| 124 | MH15CP004 | CGCTGTGAAGTATTTAACATGCAG |
|  |  | GGAGGCCTTGCACTGTTTTATGA |
| 125 | MH15KK066 | TCTATGGATCGTTCTTGCTTGTTTCT |
|  |  | GGGCTATTTTGTTGACTGAGAGAATG |
| 126 | MH15KK067 | AGGGAAAATTCTTCCTTATGATGGGAAG |
|  |  | TCCAGTTTCAATTTTCTGCACATTGTTAGA |
| 127 | MH15KK069 | TATGTTGCCCAGAATTCTGAGCATAGAC |
|  |  | AGGGAGGAAATAATTCGCTTTGCATT |
| 128 | MH15KK095 | CAGAATAGCACTGGATCCACAGGC |
|  |  | AAGCTTAATTGCCATGCCGTTTATC |
| 129 | MH16KK053 | GTGAAGACATCGTAAAAAGATCTACCTG |
|  |  | AATTTAATTGGGATCAATGCCCAAAAGG |
| 130 | MH16KK062 | TTATTACTCTAGAGGCAGGGACTAGCCT |
|  |  | AGGTATCTGCTGTCAGTGTGACTAA |
| 131 | MH16KK096 | AAGCATCTTTGGAGTTCTCTTCTCCAG |
|  |  | TAGACATATTCCTACATCTGTGGAATGG |
| 132 | MH16KK255 | CTATTTCAAGGTAAGATTCTGTCTCTTA |
|  |  | AAGAACATATTCTAAAACAGCTGAATGAAC |
| 133 | MH16KK302 | AATGTCATTGACGTGATCACCTGCA |
|  |  | GTAGTAGGCGATGAAGAGCGT |
| 134 | MH17CP001 | TGAGTTGAAACCCCAGTGAAACACA |
|  |  | CCCCAGCAATGATCTCGTAAGT |
| 135 | MH17CP006 | AACCCTTCCTCCTAACCTCATATG |
|  |  | CTTACCCAACAGAACTCAAGTATTGGT |
| 136 | MH17KK014 | TTTACTTAAAGCATAGCTTGCCTTGCC |
|  |  | CGGTTGCACCATTTGACATTCTATTAG |
| 137 | MH17KK052 | AACAGGAAAGCAGATGAAACTGGC |
|  |  | CTACTGTGCGTGTGCGATAGC |
| 138 | MH17KK053 | TGGATCACAACCTCACGGAGG |
|  |  | CGTCTTGGAAGTGAAAACACATCATA |
| 139 | MH17KK054 | GATCGCAGCGGCTACAG |
|  |  | TCCATGCACAGTCCCACGA |
| 140 | MH17KK055 | TTCATAAACAAGCAGATATGCAAGAAGA |
|  |  | CATAAGCCAGTTTCCCAGTTTTCAA |
| 141 | MH17KK077 | CTAATGCCTCTGTTCAAGCTTCTTTGC |
|  |  | TCAAATTCTTAGAGCTCCCAGCTGA |
| 142 | MH17KK105 | TTTCCTTGGATTCCACACTTTGCCT |
|  |  | AGTAGATGGGAAATCACACGCAAAT |
| 143 | MH17KK110 | GCCCAGTAAGAGCTTTCTTTTATGG |
|  |  | GATGCACGCTTATGGGTAGTGAA |
| 144 | MH17KK272 | GTCTTCCCCCAAAACTGACAG |
|  |  | GGACTCTGAAGCCTCTGTACACAT |
| 145 | MH18CP003 | CCCAAAATATTACTGCAGATGTCCTTA |
|  |  | AGCAGACTAATATGCCTCTGCTATTT |
| 146 | MH18CP005 | CTCACTTTTCAGTATTCTGTTCTGAG |
|  |  | ATTCTGACACACAAGTTTATCCATGC |
| 147 | MH18KK285 | TTCTCCTTGTTCTTCCCTGTGCATACC |
|  |  | AAGAAGCTTGAAAGTCTACAGTTGTCC |
| 148 | MH18KK293 | CTTTCCTCCCCATCAATCACTTGGG |
|  |  | TCAAGGCTATGGATACCTATCTCTTCT |
| 149 | MH19CP007 | CCCAGTTCGGCATCCGTAAGG |
|  |  | GGTGCCCAGATATGGAGGGAA |
| 150 | MH19KK056 | CAACTAGAGATCACCCCATAACTCAG |
|  |  | TAAAAATGAAGATTCGGCCGGAC |
| 151 | MH19KK057 | AAACAGAAGAGCATATTGGCCACAAT |
|  |  | GCAGTTAGGCACTAAACTATATTGTTTCAAA |
| 152 | MH19KK299 | CACTCCATCGTGAAAGAATAATCCTGT |
|  |  | GGTTAAGCTGCTTTGAGGAACAAGA |
| 153 | MH19KK301 | GAATCCTAAGATTGTGGCTGAGAGAG |
|  |  | GTTCTTTCCTCCTGACATGGGAAC |
| 154 | MH20KK058 | CCAAAAGTAAGAACTGCTTCAGGGA |
|  |  | ATGAGCCACATTACTTTGTTTTCTAGG |
| 155 | MH20KK059 | TGTGGTGATGACTGAGAGATGATGC |
|  |  | CCATAGACCAGTGGATGCCAAC |
| 156 | MH20KK307 | TGTGAGTCCTCTCGGTCATAGCA |
|  |  | CATGGCATTATCAGGGTCTGAAGAAA |
| 157 | MH21KK313 | AAAGCTTATGTGGTAGGAGCCTAA |
|  |  | CAACAAGAGAGGACAAATTCTTTCACA |
| 158 | MH21KK315 | GTACCTAGCTTAGGGTTAGACATCTG |
|  |  | TGTGCAGAAATAACAGAGTGAGAAAGT |
| 159 | MH21KK316 | GAAGTCCAAAGTCAAAGTGTCAGCA |
|  |  | TGTTTTGGATGATATGTTTCCTTTTGTTCATT |
| 160 | MH21KK324 | AGAGGAGCTTCACAAACATCCGCT |
|  |  | CTGCTGGTGAATCAGCAAAACCT |
| 161 | MH22KK060 | TTATCGGCTGGAACGAGTTCA |
|  |  | GGTGATAACAGCTTCTCCTGTAAGG |
| 162 | MH22KK064 | CGTGGACGCCGTGATTCAG |
|  |  | GTGATAGTGGGTTTTCAGTGAACG |
| 163 | MH22KK303 | GAGCCAATCTTCAGTCAGTACCGC |
|  |  | CCTGTGGTCACAGTTCTTGGTC |

**Supplementary Table 5. Sequencing quality statistics of 163 MH loci**

| No. | MH | Call rate | DoC (×) | % Allele | % Noise | ACR |
| --- | --- | --- | --- | --- | --- | --- |
| 1 | MH01CP007 | 100.00% | 6401.48 | 99.76% | 0.24% | 95.94% |
| 2 | MH01CP008 | 100.00% | 471.91 | 99.57% | 0.43% | 67.92% |
| 3 | MH01CP012 | 100.00% | 361.73 | 99.78% | 0.22% | 91.43% |
| 4 | MH01CP016 | 100.00% | 821.12 | 99.71% | 0.29% | 92.91% |
| 5 | MH01KK001 | 100.00% | 6313.22 | 99.60% | 0.40% | 94.45% |
| 6 | MH01KK070 | 100.00% | 101.74 | 99.72% | 0.28% | 84.38% |
| 7 | MH01KK072 | 100.00% | 577.7 | 99.89% | 0.11% | 93.02% |
| 8 | MH01KK106 | 96.74% | 190.65 | 99.51% | 0.49% | 77.10% |
| 9 | MH01KK117 | 100.00% | 5841.37 | 99.50% | 0.50% | 96.63% |
| 10 | MH01KK172 | 100.00% | 6262.25 | 99.58% | 0.42% | 95.70% |
| 11 | MH01KK205 | 100.00% | 1489.74 | 99.55% | 0.45% | 90.92% |
| 12 | MH01KK210 | 100.00% | 4387.18 | 99.75% | 0.25% | 95.43% |
| 13 | MH01KK211 | 100.00% | 1940.76 | 99.51% | 0.49% | 94.79% |
| 14 | MH02CP004 | 100.00% | 953.76 | 98.25% | 1.75% | 93.37% |
| 15 | MH02KK003 | 100.00% | 1239.13 | 99.73% | 0.27% | 92.07% |
| 16 | MH02KK004 | 100.00% | 5672.85 | 99.50% | 0.50% | 84.11% |
| 17 | MH02KK073 | 100.00% | 581.62 | 99.69% | 0.31% | 91.01% |
| 18 | MH02KK102 | 100.00% | 198.29 | 99.69% | 0.31% | NA |
| 19 | MH02KK105 | 100.00% | 7351.42 | 99.76% | 0.24% | 94.23% |
| 20 | MH02KK131 | 100.00% | 6262.05 | 99.89% | 0.11% | 93.42% |
| 21 | MH02KK134 | 100.00% | 252.62 | 99.64% | 0.36% | 88.46% |
| 22 | MH02KK136 | 100.00% | 5170.7 | 99.56% | 0.44% | 95.51% |
| 23 | MH02KK138 | 100.00% | 525.34 | 99.39% | 0.61% | 92.38% |
| 24 | MH02KK139 | 100.00% | 390.61 | 99.73% | 0.27% | 92.21% |
| 25 | MH02KK201 | 100.00% | 5994.35 | 99.79% | 0.21% | 97.23% |
| 26 | MH02KK202 | 100.00% | 237.57 | 99.82% | 0.18% | 88.15% |
| 27 | MH02KK213 | 100.00% | 81.39 | 99.80% | 0.20% | 80.62% |
| 28 | MH02KK215 | 100.00% | 6304.1 | 99.78% | 0.22% | 96.01% |
| 29 | MH03KK006 | 100.00% | 287.33 | 99.81% | 0.19% | 88.79% |
| 30 | MH03KK007 | 100.00% | 421.91 | 99.81% | 0.19% | 92.16% |
| 31 | MH03KK008 | 100.00% | 359.59 | 99.61% | 0.39% | 90.54% |
| 32 | MH03KK009 | 100.00% | 2497.39 | 99.75% | 0.25% | 96.10% |
| 33 | MH03KK216 | 100.00% | 231.29 | 99.89% | 0.11% | 86.52% |
| 34 | MH04CP002 | 98.91% | 111.74 | 99.35% | 0.65% | 59.04% |
| 35 | MH04CP003 | 100.00% | 7002.67 | 99.72% | 0.28% | 95.99% |
| 36 | MH04CP007 | 100.00% | 942.91 | 99.67% | 0.33% | 93.02% |
| 37 | MH04KK010 | 100.00% | 7422.63 | 99.79% | 0.21% | 97.09% |
| 38 | MH04KK011 | 100.00% | 132.36 | 99.78% | 0.22% | 71.63% |
| 39 | MH04KK013 | 100.00% | 3516.83 | 99.56% | 0.44% | 95.14% |
| 40 | MH04KK015 | 100.00% | 470.96 | 99.77% | 0.23% | 92.75% |
| 41 | MH04KK016 | 100.00% | 5253.4 | 99.76% | 0.24% | 94.88% |
| 42 | MH04KK017 | 98.91% | 74.89 | 99.72% | 0.28% | 81.73% |
| 43 | MH04KK019 | 100.00% | 2721.78 | 98.40% | 1.60% | 60.10% |
| 44 | MH04KK028 | 100.00% | 1040.9 | 99.88% | 0.12% | 93.93% |
| 45 | MH04KK029 | 100.00% | 139.42 | 99.88% | 0.12% | 84.61% |
| 46 | MH04KK030 | 100.00% | 1811.4 | 99.51% | 0.49% | 93.79% |
| 47 | MH04KK074 | 100.00% | 1711.16 | 99.81% | 0.19% | 95.99% |
| 48 | MH05CP004 | 100.00% | 2882.18 | 99.70% | 0.30% | 93.73% |
| 49 | MH05CP006 | 100.00% | 435.98 | 99.64% | 0.36% | 88.91% |
| 50 | MH05CP010 | 100.00% | 263.93 | 97.19% | 2.81% | 65.76% |
| 51 | MH05KK020 | 100.00% | 553.01 | 99.52% | 0.48% | 90.84% |
| 52 | MH05KK022 | 100.00% | 7538.5 | 99.78% | 0.22% | 95.76% |
| 53 | MH05KK062 | 100.00% | 3002.96 | 99.76% | 0.24% | 93.44% |
| 54 | MH05KK078 | 100.00% | 1432.05 | 99.82% | 0.18% | 94.90% |
| 55 | MH05KK079 | 100.00% | 4005.82 | 99.86% | 0.14% | 96.81% |
| 56 | MH05KK122 | 100.00% | 6736.11 | 99.77% | 0.23% | 94.30% |
| 57 | MH05KK123 | 100.00% | 5541.84 | 99.81% | 0.19% | 95.06% |
| 58 | MH05KK124 | 100.00% | 283.25 | 99.69% | 0.31% | 90.84% |
| 59 | MH05KK170 | 100.00% | 1411.64 | 99.47% | 0.53% | 93.71% |
| 60 | MH06CP003 | 100.00% | 1788.75 | 99.73% | 0.27% | 94.97% |
| 61 | MH06CP007 | 100.00% | 184.8 | 81.78% | 18.22% | 82.90% |
| 62 | MH06KK026 | 100.00% | 6996.26 | 99.77% | 0.23% | 97.18% |
| 63 | MH06KK030 | 100.00% | 1251.92 | 99.79% | 0.21% | 92.39% |
| 64 | MH06KK031 | 100.00% | 4724.02 | 98.68% | 1.32% | 81.34% |
| 65 | MH06KK080 | 100.00% | 1546.83 | 99.76% | 0.24% | NA |
| 66 | MH06KK101 | 100.00% | 5890.01 | 99.62% | 0.38% | 91.17% |
| 67 | MH07KK030 | 88.04% | 39.22 | 99.41% | 0.59% | 77.87% |
| 68 | MH07KK031 | 100.00% | 2455.49 | 99.77% | 0.23% | 91.88% |
| 69 | MH07KK081 | 100.00% | 1156.98 | 99.70% | 0.30% | 91.49% |
| 70 | MH07KK082 | 100.00% | 1234.6 | 99.78% | 0.22% | 93.24% |
| 71 | MH08KK032 | 98.91% | 84.37 | 99.90% | 0.10% | 83.69% |
| 72 | MH09KK020 | 100.00% | 1411.97 | 99.55% | 0.45% | 94.09% |
| 73 | MH09KK033 | 100.00% | 4287.91 | 99.79% | 0.21% | 95.58% |
| 74 | MH09KK034 | 100.00% | 334.14 | 99.80% | 0.20% | 88.79% |
| 75 | MH09KK152 | 98.91% | 55.49 | 99.49% | 0.51% | 73.01% |
| 76 | MH09KK153 | 98.91% | 83.55 | 99.58% | 0.42% | 82.30% |
| 77 | MH09KK157 | 100.00% | 295.77 | 99.29% | 0.71% | 84.57% |
| 78 | MH09KK161 | 100.00% | 953.2 | 99.79% | 0.21% | 94.40% |
| 79 | MH10CP003 | 100.00% | 487.43 | 98.48% | 1.52% | 81.67% |
| 80 | MH10KK083 | 100.00% | 6499.41 | 99.85% | 0.15% | 90.85% |
| 81 | MH10KK084 | 100.00% | 7210.82 | 99.79% | 0.21% | NA |
| 82 | MH10KK085 | 100.00% | 702.92 | 99.87% | 0.13% | 93.45% |
| 83 | MH10KK086 | 100.00% | 6376.07 | 99.85% | 0.15% | 95.63% |
| 84 | MH10KK087 | 100.00% | 6428.77 | 99.75% | 0.25% | 96.95% |
| 85 | MH10KK088 | 100.00% | 279.64 | 99.87% | 0.13% | 87.31% |
| 86 | MH10KK101 | 100.00% | 4077.83 | 99.75% | 0.25% | 92.23% |
| 87 | MH10KK163 | 100.00% | 1637.45 | 99.16% | 0.84% | 87.61% |
| 88 | MH10KK170 | 100.00% | 121.99 | 99.74% | 0.26% | 81.04% |
| 89 | MH11CP003 | 100.00% | 1954.57 | 99.67% | 0.33% | 94.27% |
| 90 | MH11CP004 | 100.00% | 698.6 | 99.73% | 0.27% | 90.85% |
| 91 | MH11CP005 | 100.00% | 4457.11 | 99.69% | 0.31% | 95.01% |
| 92 | MH11KK036 | 100.00% | 1032.32 | 99.78% | 0.22% | 93.36% |
| 93 | MH11KK037 | 100.00% | 734.29 | 99.81% | 0.19% | 86.19% |
| 94 | MH11KK038 | 100.00% | 3367.39 | 99.84% | 0.16% | 95.28% |
| 95 | MH11KK039 | 100.00% | 957.21 | 99.73% | 0.27% | 93.55% |
| 96 | MH11KK040 | 100.00% | 214.86 | 99.84% | 0.16% | 87.13% |
| 97 | MH11KK041 | 100.00% | 139.07 | 99.83% | 0.17% | 87.16% |
| 98 | MH11KK089 | 100.00% | 366.91 | 99.86% | 0.14% | 89.03% |
| 99 | MH11KK090 | 100.00% | 2616.2 | 99.81% | 0.19% | 96.19% |
| 100 | MH11KK091 | 100.00% | 517.58 | 99.53% | 0.47% | 88.05% |
| 101 | MH11KK180 | 100.00% | 5866.57 | 99.53% | 0.47% | 93.69% |
| 102 | MH11KK187 | 100.00% | 4856.66 | 99.60% | 0.40% | 95.02% |
| 103 | MH11KK191 | 93.48% | 52.35 | 98.98% | 1.02% | 74.03% |
| 104 | MH12KK042 | 100.00% | 5175.68 | 99.86% | 0.14% | 96.90% |
| 105 | MH12KK043 | 98.91% | 147.47 | 99.79% | 0.21% | 81.19% |
| 106 | MH12KK045 | 100.00% | 960.54 | 99.86% | 0.14% | 93.45% |
| 107 | MH12KK046 | 100.00% | 427.8 | 99.59% | 0.41% | 83.82% |
| 108 | MH12KK092 | 100.00% | 7495.8 | 99.74% | 0.26% | 95.79% |
| 109 | MH12KK093 | 100.00% | 2776 | 99.73% | 0.27% | 96.78% |
| 110 | MH12KK202 | 100.00% | 775.8 | 99.34% | 0.66% | 90.88% |
| 111 | MH13CP008 | 100.00% | 7080.91 | 99.66% | 0.34% | 92.88% |
| 112 | MH13KK047 | 100.00% | 1752.85 | 99.75% | 0.25% | 91.51% |
| 113 | MH13KK213 | 100.00% | 106.96 | 99.61% | 0.39% | 81.83% |
| 114 | MH13KK217 | 100.00% | 5219.24 | 99.43% | 0.57% | 91.21% |
| 115 | MH13KK218 | 100.00% | 3550.71 | 99.62% | 0.38% | 94.04% |
| 116 | MH13KK225 | 100.00% | 66.48 | 99.66% | 0.34% | 79.33% |
| 117 | MH13KK226 | 100.00% | 2521.41 | 99.67% | 0.33% | 89.01% |
| 118 | MH14CP003 | 100.00% | 5274.16 | 99.68% | 0.32% | 95.13% |
| 119 | MH14CP004 | 100.00% | 3343.14 | 99.76% | 0.24% | 96.24% |
| 120 | MH14KK048 | 100.00% | 126.67 | 99.81% | 0.19% | 86.93% |
| 121 | MH14KK101 | 100.00% | 1418.84 | 99.86% | 0.14% | 92.42% |
| 122 | MH15CP001 | 71.74% | 2804.74 | 92.94% | 7.06% | 88.20% |
| 123 | MH15CP003 | 100.00% | 3561.14 | 99.45% | 0.55% | 93.59% |
| 124 | MH15CP004 | 100.00% | 5506.09 | 99.33% | 0.67% | 95.17% |
| 125 | MH15KK066 | 100.00% | 188.72 | 99.61% | 0.39% | 88.06% |
| 126 | MH15KK067 | 100.00% | 1759.09 | 99.71% | 0.29% | 93.05% |
| 127 | MH15KK069 | 100.00% | 3074.74 | 99.80% | 0.20% | 95.89% |
| 128 | MH15KK095 | 100.00% | 426.96 | 99.85% | 0.15% | 91.31% |
| 129 | MH16KK053 | 100.00% | 5454.52 | 99.75% | 0.25% | 95.84% |
| 130 | MH16KK062 | 100.00% | 2144.63 | 99.75% | 0.25% | 94.46% |
| 131 | MH16KK096 | 100.00% | 5123.82 | 99.80% | 0.20% | 90.26% |
| 132 | MH16KK255 | 100.00% | 1152.77 | 99.54% | 0.46% | 93.19% |
| 133 | MH16KK302 | 100.00% | 437.14 | 99.53% | 0.47% | 91.61% |
| 134 | MH17CP001 | 100.00% | 6812.98 | 99.59% | 0.41% | 92.61% |
| 135 | MH17CP006 | 100.00% | 784.52 | 99.79% | 0.21% | 92.89% |
| 136 | MH17KK014 | 100.00% | 1188.2 | 99.86% | 0.14% | 91.61% |
| 137 | MH17KK052 | 100.00% | 2319.05 | 99.73% | 0.27% | 94.74% |
| 138 | MH17KK053 | 92.39% | 35.62 | 99.74% | 0.26% | 77.10% |
| 139 | MH17KK054 | 100.00% | 760.22 | 99.82% | 0.18% | 92.53% |
| 140 | MH17KK055 | 100.00% | 2551.34 | 99.72% | 0.28% | 87.66% |
| 141 | MH17KK077 | 100.00% | 5404.09 | 99.86% | 0.14% | 47.79% |
| 142 | MH17KK105 | 95.65% | 30.25 | 97.53% | 2.47% | 88.11% |
| 143 | MH17KK110 | 100.00% | 3310.77 | 99.92% | 0.08% | 95.67% |
| 144 | MH17KK272 | 98.91% | 92.69 | 99.37% | 0.63% | 79.76% |
| 145 | MH18CP003 | 100.00% | 2845.91 | 99.74% | 0.26% | 94.62% |
| 146 | MH18CP005 | 100.00% | 1249.08 | 99.59% | 0.41% | 93.78% |
| 147 | MH18KK285 | 100.00% | 94.04 | 99.58% | 0.42% | 83.21% |
| 148 | MH18KK293 | 100.00% | 1595.4 | 99.37% | 0.63% | 93.61% |
| 149 | MH19CP007 | 100.00% | 2815.92 | 99.56% | 0.44% | 91.93% |
| 150 | MH19KK056 | 100.00% | 5393 | 99.47% | 0.53% | 95.77% |
| 151 | MH19KK057 | 100.00% | 2989.26 | 99.82% | 0.18% | 94.01% |
| 152 | MH19KK299 | 100.00% | 546.29 | 99.32% | 0.68% | 86.82% |
| 153 | MH19KK301 | 100.00% | 184.03 | 99.55% | 0.45% | 87.40% |
| 154 | MH20KK058 | 100.00% | 169.79 | 99.75% | 0.25% | 85.93% |
| 155 | MH20KK059 | 100.00% | 2320.93 | 99.77% | 0.23% | 88.81% |
| 156 | MH20KK307 | 100.00% | 83.54 | 99.58% | 0.42% | 81.68% |
| 157 | MH21KK313 | 100.00% | 189.04 | 99.81% | 0.19% | 87.40% |
| 158 | MH21KK315 | 89.13% | 31.99 | 98.80% | 1.20% | 74.30% |
| 159 | MH21KK316 | 100.00% | 158.38 | 99.65% | 0.35% | 84.57% |
| 160 | MH21KK324 | 100.00% | 2667.03 | 99.41% | 0.59% | 92.37% |
| 161 | MH22KK060 | 100.00% | 3444.84 | 99.86% | 0.14% | 94.18% |
| 162 | MH22KK064 | 100.00% | 296.5 | 99.46% | 0.54% | 89.61% |
| 163 | MH22KK303 | 100.00% | 1871.85 | 99.70% | 0.30% | 93.28% |

NA: Heterozygous alleles were not detected at MH02KK102, MH06KK080 and MH10KK084.

**Supplementary Table 6.** Alleles and frequencies of 163 MH loci in the CHE population (N=92).

| No. | MH | Alleles | Frequencies | No. | MH | Alleles | Frequencies |
| --- | --- | --- | --- | --- | --- | --- | --- |
| 1 | MH01CP007 | ACG | 0.255 | 306 | MH10KK163 | AAACT | 0.011 |
| 2 | MH01CP007 | GAG | 0.005 | 307 | MH10KK163 | AAATT | 0.359 |
| 3 | MH01CP007 | GCG | 0.739 | 308 | MH10KK163 | AAGCC | 0.163 |
| 4 | MH01CP008 | AGA | 0.163 | 309 | MH10KK163 | AAGCT | 0.060 |
| 5 | MH01CP008 | GCA | 0.185 | 310 | MH10KK163 | TAGCC | 0.016 |
| 6 | MH01CP008 | GCT | 0.451 | 311 | MH10KK163 | TAGCT | 0.255 |
| 7 | MH01CP008 | GGA | 0.201 | 312 | MH10KK163 | TGGCC | 0.033 |
| 8 | MH01CP012 | CAC | 0.022 | 313 | MH10KK163 | TGGTC | 0.103 |
| 9 | MH01CP012 | CGC | 0.174 | 314 | MH10KK170 | GAA | 0.087 |
| 10 | MH01CP012 | TAC | 0.299 | 315 | MH10KK170 | GAG | 0.016 |
| 11 | MH01CP012 | TAT | 0.054 | 316 | MH10KK170 | TGA | 0.897 |
| 12 | MH01CP012 | TGT | 0.451 | 317 | MH11CP003 | GTT | 0.310 |
| 13 | MH01CP016 | AGA | 0.043 | 318 | MH11CP003 | TCT | 0.359 |
| 14 | MH01CP016 | TAA | 0.424 | 319 | MH11CP003 | TTG | 0.168 |
| 15 | MH01CP016 | TGA | 0.239 | 320 | MH11CP003 | TTT | 0.163 |
| 16 | MH01CP016 | TGG | 0.293 | 321 | MH11CP004 | CGA | 0.217 |
| 17 | MH01KK001 | CAGC | 0.022 | 322 | MH11CP004 | CGG | 0.500 |
| 18 | MH01KK001 | CGGT | 0.011 | 323 | MH11CP004 | CTA | 0.125 |
| 19 | MH01KK001 | TAGT | 0.457 | 324 | MH11CP004 | TGG | 0.152 |
| 20 | MH01KK001 | TGCC | 0.152 | 325 | MH11CP005 | CGT | 0.179 |
| 21 | MH01KK001 | TGGC | 0.359 | 326 | MH11CP005 | TAC | 0.359 |
| 22 | MH01KK070 | AG | 0.168 | 327 | MH11CP005 | TAT | 0.120 |
| 23 | MH01KK070 | AT | 0.832 | 328 | MH11CP005 | TGT | 0.342 |
| 24 | MH01KK072 | CG | 0.641 | 329 | MH11KK036 | AA | 0.364 |
| 25 | MH01KK072 | TC | 0.359 | 330 | MH11KK036 | AG | 0.261 |
| 26 | MH01KK106 | CAAG | 0.011 | 331 | MH11KK036 | CG | 0.375 |
| 27 | MH01KK106 | CAGA | 0.511 | 332 | MH11KK037 | ACG | 0.533 |
| 28 | MH01KK106 | CAGG | 0.112 | 333 | MH11KK037 | GCG | 0.326 |
| 29 | MH01KK106 | CGAG | 0.112 | 334 | MH11KK037 | GTG | 0.141 |
| 30 | MH01KK106 | TAGG | 0.253 | 335 | MH11KK038 | CG | 0.554 |
| 31 | MH01KK117 | AACC | 0.413 | 336 | MH11KK038 | TA | 0.065 |
| 32 | MH01KK117 | AACT | 0.136 | 337 | MH11KK038 | TG | 0.380 |
| 33 | MH01KK117 | AGCC | 0.103 | 338 | MH11KK039 | GG | 0.054 |
| 34 | MH01KK117 | AGCT | 0.027 | 339 | MH11KK039 | GT | 0.446 |
| 35 | MH01KK117 | CACC | 0.179 | 340 | MH11KK039 | TT | 0.500 |
| 36 | MH01KK117 | CACT | 0.022 | 341 | MH11KK040 | AC | 0.429 |
| 37 | MH01KK117 | CAGC | 0.027 | 342 | MH11KK040 | CC | 0.505 |
| 38 | MH01KK117 | CGCC | 0.027 | 343 | MH11KK040 | CG | 0.065 |
| 39 | MH01KK172 | ACA | 0.038 | 344 | MH11KK041 | AG | 0.065 |
| 40 | MH01KK172 | ACG | 0.473 | 345 | MH11KK041 | GA | 0.516 |
| 41 | MH01KK172 | ATA | 0.255 | 346 | MH11KK041 | GG | 0.418 |
| 42 | MH01KK172 | ATG | 0.234 | 347 | MH11KK089 | AT | 0.516 |
| 43 | MH01KK205 | CCAG | 0.348 | 348 | MH11KK089 | CG | 0.473 |
| 44 | MH01KK205 | TCAG | 0.174 | 349 | MH11KK089 | CT | 0.011 |
| 45 | MH01KK205 | TTAA | 0.158 | 350 | MH11KK090 | AC | 0.565 |
| 46 | MH01KK205 | TTAG | 0.082 | 351 | MH11KK090 | GT | 0.435 |
| 47 | MH01KK205 | TTGG | 0.239 | 352 | MH11KK091 | DT | 0.071 |
| 48 | MH01KK210 | CC | 0.179 | 353 | MH11KK091 | IC | 0.228 |
| 49 | MH01KK210 | TC | 0.272 | 354 | MH11KK091 | IT | 0.701 |
| 50 | MH01KK210 | TT | 0.549 | 355 | MH11KK180 | AATC | 0.054 |
| 51 | MH01KK211 | ACT | 0.092 | 356 | MH11KK180 | AACC | 0.016 |
| 52 | MH01KK211 | ATC | 0.484 | 357 | MH11KK180 | AATG | 0.033 |
| 53 | MH01KK211 | ATT | 0.223 | 358 | MH11KK180 | ACCC | 0.435 |
| 54 | MH01KK211 | GTC | 0.201 | 359 | MH11KK180 | ACCG | 0.005 |
| 55 | MH02CP004 | CGA | 0.005 | 360 | MH11KK180 | ACTC | 0.185 |
| 56 | MH02CP004 | CGG | 0.332 | 361 | MH11KK180 | ACTG | 0.038 |
| 57 | MH02CP004 | CTA | 0.451 | 362 | MH11KK180 | GCCG | 0.217 |
| 58 | MH02CP004 | CTG | 0.103 | 363 | MH11KK180 | GCTC | 0.011 |
| 59 | MH02CP004 | TTG | 0.109 | 364 | MH11KK187 | CCCA | 0.413 |
| 60 | MH02KK003 | GTC | 0.043 | 365 | MH11KK187 | GCGG | 0.364 |
| 61 | MH02KK003 | TCC | 0.853 | 366 | MH11KK187 | GTGG | 0.212 |
| 62 | MH02KK003 | TTC | 0.033 | 367 | MH11KK191 | CAGT | 0.221 |
| 63 | MH02KK003 | TTT | 0.071 | 368 | MH11KK191 | CGAT | 0.093 |
| 64 | MH02KK073 | GC | 0.793 | 369 | MH11KK191 | TAAC | 0.192 |
| 65 | MH02KK073 | GT | 0.071 | 370 | MH11KK191 | TAAT | 0.494 |
| 66 | MH02KK073 | TT | 0.136 | 371 | MH12KK042 | CA | 0.022 |
| 67 | MH02KK004 | GAA | 0.201 | 372 | MH12KK042 | CG | 0.424 |
| 68 | MH02KK004 | GAG | 0.141 | 373 | MH12KK042 | TG | 0.554 |
| 69 | MH02KK004 | GGA | 0.158 | 374 | MH12KK043 | CCG | 0.137 |
| 70 | MH02KK004 | TAG | 0.500 | 375 | MH12KK043 | CTA | 0.462 |
| 71 | MH02KK102 | GGT | 1.000 | 376 | MH12KK043 | CTG | 0.401 |
| 72 | MH02KK105 | AA | 0.495 | 377 | MH12KK045 | CT | 0.054 |
| 73 | MH02KK105 | GA | 0.370 | 378 | MH12KK045 | TC | 0.734 |
| 74 | MH02KK105 | GG | 0.136 | 379 | MH12KK045 | TT | 0.212 |
| 75 | MH02KK131 | CA | 0.005 | 380 | MH12KK046 | GA | 0.223 |
| 76 | MH02KK131 | GA | 0.288 | 381 | MH12KK046 | GG | 0.293 |
| 77 | MH02KK131 | GG | 0.707 | 382 | MH12KK046 | TA | 0.288 |
| 78 | MH02KK134 | ACCG | 0.060 | 383 | MH12KK046 | TG | 0.190 |
| 79 | MH02KK134 | ACTG | 0.016 | 384 | MH12KK092 | CT | 0.326 |
| 80 | MH02KK134 | ATCA | 0.033 | 385 | MH12KK092 | TC | 0.674 |
| 81 | MH02KK134 | ATCG | 0.489 | 386 | MH12KK093 | AT | 0.750 |
| 82 | MH02KK134 | ATTA | 0.103 | 387 | MH12KK093 | TA | 0.250 |
| 83 | MH02KK134 | ATTG | 0.076 | 388 | MH12KK202 | AACT | 0.179 |
| 84 | MH02KK134 | TCTG | 0.016 | 389 | MH12KK202 | AATC | 0.391 |
| 85 | MH02KK134 | TTCG | 0.027 | 390 | MH12KK202 | AGTT | 0.185 |
| 86 | MH02KK134 | TTTG | 0.163 | 391 | MH12KK202 | CATT | 0.245 |
| 87 | MH02KK136 | GTA | 0.082 | 392 | MH13CP008 | CAC | 0.435 |
| 88 | MH02KK136 | GTC | 0.071 | 393 | MH13CP008 | CAT | 0.054 |
| 89 | MH02KK136 | TCA | 0.332 | 394 | MH13CP008 | CGT | 0.266 |
| 90 | MH02KK136 | TCC | 0.158 | 395 | MH13CP008 | GAT | 0.245 |
| 91 | MH02KK136 | TTC | 0.359 | 396 | MH13KK047 | CC | 0.255 |
| 92 | MH02KK138 | ATTAGA | 0.457 | 397 | MH13KK047 | CT | 0.109 |
| 93 | MH02KK138 | GGTAAA | 0.353 | 398 | MH13KK047 | TC | 0.016 |
| 94 | MH02KK138 | GGTAGA | 0.033 | 399 | MH13KK047 | TT | 0.620 |
| 95 | MH02KK138 | GTTAGA | 0.158 | 400 | MH13KK213 | CCA | 0.337 |
| 96 | MH02KK139 | AA | 0.033 | 401 | MH13KK213 | CCG | 0.147 |
| 97 | MH02KK139 | AG | 0.027 | 402 | MH13KK213 | TAG | 0.250 |
| 98 | MH02KK139 | GA | 0.940 | 403 | MH13KK213 | TCA | 0.245 |
| 99 | MH02KK201 | GA | 0.011 | 404 | MH13KK213 | TCG | 0.022 |
| 100 | MH02KK201 | GG | 0.049 | 405 | MH13KK217 | AACG | 0.109 |
| 101 | MH02KK201 | TA | 0.940 | 406 | MH13KK217 | AATG | 0.375 |
| 102 | MH02KK202 | CA | 0.418 | 407 | MH13KK217 | AGCA | 0.152 |
| 103 | MH02KK202 | GA | 0.582 | 408 | MH13KK217 | AGCG | 0.092 |
| 104 | MH02KK213 | CAT | 0.071 | 409 | MH13KK217 | AGTG | 0.152 |
| 105 | MH02KK213 | CGT | 0.337 | 410 | MH13KK217 | GGCG | 0.109 |
| 106 | MH02KK213 | TGT | 0.592 | 411 | MH13KK217 | GGTG | 0.005 |
| 107 | MH02KK215 | AT | 0.103 | 412 | MH13KK218 | CCCC | 0.027 |
| 108 | MH02KK215 | CC | 0.304 | 413 | MH13KK218 | CCCT | 0.016 |
| 109 | MH02KK215 | CT | 0.592 | 414 | MH13KK218 | CTCC | 0.114 |
| 110 | MH03KK006 | AA | 0.636 | 415 | MH13KK218 | CTCT | 0.125 |
| 111 | MH03KK006 | AG | 0.315 | 416 | MH13KK218 | CTTC | 0.179 |
| 112 | MH03KK006 | TA | 0.049 | 417 | MH13KK218 | CTTT | 0.071 |
| 113 | MH03KK007 | CC | 0.424 | 418 | MH13KK218 | TTCC | 0.060 |
| 114 | MH03KK007 | TC | 0.283 | 419 | MH13KK218 | TTCT | 0.212 |
| 115 | MH03KK007 | TT | 0.293 | 420 | MH13KK218 | TTTC | 0.033 |
| 116 | MH03KK008 | CT | 0.027 | 421 | MH13KK218 | TTTT | 0.158 |
| 117 | MH03KK008 | TG | 0.495 | 422 | MH13KK225 | AAG | 0.109 |
| 118 | MH03KK008 | TT | 0.478 | 423 | MH13KK225 | ACG | 0.446 |
| 119 | MH03KK009 | CC | 0.016 | 424 | MH13KK225 | GAA | 0.179 |
| 120 | MH03KK009 | TC | 0.217 | 425 | MH13KK225 | GAG | 0.266 |
| 121 | MH03KK009 | TT | 0.766 | 426 | MH13KK226 | CA | 0.016 |
| 122 | MH03KK216 | CC | 0.147 | 427 | MH13KK226 | CG | 0.332 |
| 123 | MH03KK216 | CT | 0.190 | 428 | MH13KK226 | TA | 0.652 |
| 124 | MH03KK216 | TC | 0.663 | 429 | MH14CP003 | AGG | 0.082 |
| 125 | MH04CP002 | CAA | 0.187 | 430 | MH14CP003 | GAA | 0.076 |
| 126 | MH04CP002 | CGA | 0.302 | 431 | MH14CP003 | GAG | 0.326 |
| 127 | MH04CP002 | CGT | 0.346 | 432 | MH14CP003 | GGA | 0.446 |
| 128 | MH04CP002 | GAA | 0.165 | 433 | MH14CP003 | GGG | 0.071 |
| 129 | MH04CP003 | ACC | 0.234 | 434 | MH14CP004 | CCG | 0.011 |
| 130 | MH04CP003 | ACT | 0.402 | 435 | MH14CP004 | CTA | 0.212 |
| 131 | MH04CP003 | GCC | 0.217 | 436 | MH14CP004 | CTG | 0.250 |
| 132 | MH04CP003 | GTC | 0.147 | 437 | MH14CP004 | GCG | 0.364 |
| 133 | MH04CP007 | AGC | 0.158 | 438 | MH14CP004 | GTG | 0.163 |
| 134 | MH04CP007 | ATC | 0.293 | 439 | MH14KK048 | AC | 0.011 |
| 135 | MH04CP007 | GGC | 0.418 | 440 | MH14KK048 | AT | 0.685 |
| 136 | MH04CP007 | GGT | 0.130 | 441 | MH14KK048 | GC | 0.098 |
| 137 | MH04KK010 | AA | 0.696 | 442 | MH14KK048 | GT | 0.207 |
| 138 | MH04KK010 | AG | 0.228 | 443 | MH14KK101 | AT | 0.130 |
| 139 | MH04KK010 | GA | 0.076 | 444 | MH14KK101 | GT | 0.870 |
| 140 | MH04KK011 | AC | 0.418 | 445 | MH15CP001 | AAA | 0.144 |
| 141 | MH04KK011 | AT | 0.261 | 446 | MH15CP001 | AAT | 0.333 |
| 142 | MH04KK011 | GT | 0.321 | 447 | MH15CP001 | ATT | 0.174 |
| 143 | MH04KK013 | AAGAT | 0.082 | 448 | MH15CP001 | TTT | 0.348 |
| 144 | MH04KK013 | CAGAT | 0.120 | 449 | MH15CP003 | AAC | 0.266 |
| 145 | MH04KK013 | CAGGT | 0.005 | 450 | MH15CP003 | AGA | 0.592 |
| 146 | MH04KK013 | CGAAT | 0.005 | 451 | MH15CP003 | AGC | 0.022 |
| 147 | MH04KK013 | CGGAC | 0.065 | 452 | MH15CP003 | CGC | 0.120 |
| 148 | MH04KK013 | CGGAT | 0.125 | 453 | MH15CP004 | CAA | 0.533 |
| 149 | MH04KK013 | CGGGT | 0.598 | 454 | MH15CP004 | TAA | 0.125 |
| 150 | MH04KK015 | AC | 0.712 | 455 | MH15CP004 | TAC | 0.092 |
| 151 | MH04KK015 | AT | 0.141 | 456 | MH15CP004 | TCA | 0.250 |
| 152 | MH04KK015 | TT | 0.147 | 457 | MH15KK066 | AG | 0.446 |
| 153 | MH04KK016 | CC | 0.147 | 458 | MH15KK066 | AT | 0.152 |
| 154 | MH04KK016 | TC | 0.141 | 459 | MH15KK066 | CG | 0.223 |
| 155 | MH04KK016 | TT | 0.712 | 460 | MH15KK066 | CT | 0.179 |
| 156 | MH04KK017 | ACA | 0.088 | 461 | MH15KK067 | GC | 0.533 |
| 157 | MH04KK017 | GCA | 0.055 | 462 | MH15KK067 | GT | 0.109 |
| 158 | MH04KK017 | GCG | 0.654 | 463 | MH15KK067 | TC | 0.359 |
| 159 | MH04KK017 | GTA | 0.203 | 464 | MH15KK069 | CC | 0.783 |
| 160 | MH04KK019 | AA | 0.413 | 465 | MH15KK069 | CT | 0.141 |
| 161 | MH04KK019 | AG | 0.582 | 466 | MH15KK069 | TT | 0.076 |
| 162 | MH04KK019 | GA | 0.005 | 467 | MH15KK095 | CA | 0.533 |
| 163 | MH04KK028 | CC | 0.261 | 468 | MH15KK095 | TA | 0.402 |
| 164 | MH04KK028 | TC | 0.739 | 469 | MH15KK095 | TG | 0.065 |
| 165 | MH04KK029 | TC | 0.880 | 470 | MH16KK053 | CC | 0.793 |
| 166 | MH04KK029 | TT | 0.120 | 471 | MH16KK053 | CT | 0.207 |
| 167 | MH04KK030 | TCAC | 0.380 | 472 | MH16KK062 | ATC | 0.440 |
| 168 | MH04KK030 | TCAG | 0.158 | 473 | MH16KK062 | GCT | 0.342 |
| 169 | MH04KK030 | TCGC | 0.033 | 474 | MH16KK062 | GTT | 0.217 |
| 170 | MH04KK030 | TCGG | 0.125 | 475 | MH16KK096 | CA | 0.668 |
| 171 | MH04KK030 | TTAC | 0.033 | 476 | MH16KK096 | CG | 0.332 |
| 172 | MH04KK030 | TTAG | 0.250 | 477 | MH16KK255 | ACCG | 0.087 |
| 173 | MH04KK030 | TTGG | 0.011 | 478 | MH16KK255 | ACTG | 0.272 |
| 174 | MH04KK074 | AT | 0.918 | 479 | MH16KK255 | GACA | 0.337 |
| 175 | MH04KK074 | GT | 0.082 | 480 | MH16KK255 | GACG | 0.011 |
| 176 | MH05CP004 | ATT | 0.245 | 481 | MH16KK255 | GATA | 0.033 |
| 177 | MH05CP004 | CCC | 0.043 | 482 | MH16KK255 | GCCG | 0.114 |
| 178 | MH05CP004 | CCT | 0.364 | 483 | MH16KK255 | GCTG | 0.147 |
| 179 | MH05CP004 | CTC | 0.212 | 484 | MH16KK302 | ACTT | 0.038 |
| 180 | MH05CP004 | CTT | 0.136 | 485 | MH16KK302 | GCTC | 0.272 |
| 181 | MH05CP006 | CCG | 0.071 | 486 | MH16KK302 | GCTT | 0.239 |
| 182 | MH05CP006 | CTA | 0.005 | 487 | MH16KK302 | GTAT | 0.342 |
| 183 | MH05CP006 | CTG | 0.440 | 488 | MH16KK302 | GTTT | 0.109 |
| 184 | MH05CP006 | TCG | 0.234 | 489 | MH17CP001 | CAG | 0.413 |
| 185 | MH05CP006 | TTA | 0.250 | 490 | MH17CP001 | GAG | 0.120 |
| 186 | MH05CP010 | CACT | 0.033 | 491 | MH17CP001 | GGA | 0.163 |
| 187 | MH05CP010 | CCCT | 0.005 | 492 | MH17CP001 | GGG | 0.304 |
| 188 | MH05CP010 | TAAG | 0.245 | 493 | MH17CP006 | CCT | 0.076 |
| 189 | MH05CP010 | TACT | 0.717 | 494 | MH17CP006 | CTC | 0.266 |
| 190 | MH05KK020 | GCGG | 0.038 | 495 | MH17CP006 | CTT | 0.212 |
| 191 | MH05KK020 | TCGG | 0.223 | 496 | MH17CP006 | GTC | 0.446 |
| 192 | MH05KK020 | TCGT | 0.402 | 497 | MH17KK014 | CCC | 0.962 |
| 193 | MH05KK020 | TTGG | 0.304 | 498 | MH17KK014 | CCT | 0.005 |
| 194 | MH05KK020 | TTTG | 0.033 | 499 | MH17KK014 | GCC | 0.022 |
| 195 | MH05KK022 | CA | 0.516 | 500 | MH17KK014 | GCT | 0.011 |
| 196 | MH05KK022 | CC | 0.272 | 501 | MH17KK052 | AA | 0.196 |
| 197 | MH05KK022 | TC | 0.212 | 502 | MH17KK052 | AG | 0.326 |
| 198 | MH05KK062 | AA | 0.293 | 503 | MH17KK052 | GA | 0.467 |
| 199 | MH05KK062 | AC | 0.489 | 504 | MH17KK052 | GG | 0.011 |
| 200 | MH05KK062 | TA | 0.217 | 505 | MH17KK053 | CT | 0.418 |
| 201 | MH05KK078 | GA | 0.130 | 506 | MH17KK053 | TC | 0.418 |
| 202 | MH05KK078 | GG | 0.870 | 507 | MH17KK053 | TT | 0.165 |
| 203 | MH05KK079 | CC | 0.565 | 508 | MH17KK054 | AA | 0.478 |
| 204 | MH05KK079 | CT | 0.435 | 509 | MH17KK054 | AG | 0.223 |
| 205 | MH05KK122 | AC | 0.174 | 510 | MH17KK054 | GG | 0.299 |
| 206 | MH05KK122 | CA | 0.109 | 511 | MH17KK055 | AC | 0.543 |
| 207 | MH05KK122 | CC | 0.717 | 512 | MH17KK055 | CC | 0.082 |
| 208 | MH05KK123 | AC | 0.386 | 513 | MH17KK055 | CT | 0.375 |
| 209 | MH05KK123 | GC | 0.391 | 514 | MH17KK077 | GG | 0.891 |
| 210 | MH05KK123 | GT | 0.223 | 515 | MH17KK077 | TG | 0.109 |
| 211 | MH05KK124 | CA | 0.190 | 516 | MH17KK105 | ATA | 0.028 |
| 212 | MH05KK124 | TA | 0.658 | 517 | MH17KK105 | ATG | 0.972 |
| 213 | MH05KK124 | TG | 0.152 | 518 | MH17KK110 | CG | 0.902 |
| 214 | MH05KK170 | CAAA | 0.054 | 519 | MH17KK110 | TG | 0.098 |
| 215 | MH05KK170 | CAAG | 0.098 | 520 | MH17KK272 | CCCT | 0.462 |
| 216 | MH05KK170 | CAGG | 0.054 | 521 | MH17KK272 | TCAT | 0.110 |
| 217 | MH05KK170 | CGAA | 0.120 | 522 | MH17KK272 | TCCC | 0.033 |
| 218 | MH05KK170 | CGAG | 0.147 | 523 | MH17KK272 | TCCT | 0.231 |
| 219 | MH05KK170 | CGGA | 0.033 | 524 | MH17KK272 | TTCC | 0.165 |
| 220 | MH05KK170 | CGGG | 0.027 | 525 | MH18CP003 | ACT | 0.266 |
| 221 | MH05KK170 | TAAA | 0.179 | 526 | MH18CP003 | ATC | 0.370 |
| 222 | MH05KK170 | TAAG | 0.283 | 527 | MH18CP003 | ATT | 0.261 |
| 223 | MH06CP003 | AAA | 0.332 | 528 | MH18CP003 | GTC | 0.103 |
| 224 | MH06CP003 | AAC | 0.141 | 529 | MH18CP005 | ACAT | 0.283 |
| 225 | MH06CP003 | AGC | 0.348 | 530 | MH18CP005 | ACGC | 0.266 |
| 226 | MH06CP003 | GGC | 0.179 | 531 | MH18CP005 | ATAC | 0.321 |
| 227 | MH06CP007 | AAG | 0.190 | 532 | MH18CP005 | GCAC | 0.130 |
| 228 | MH06CP007 | AAT | 0.179 | 533 | MH18KK285 | AGCG | 0.114 |
| 229 | MH06CP007 | AGG | 0.201 | 534 | MH18KK285 | CACG | 0.533 |
| 230 | MH06CP007 | AGT | 0.005 | 535 | MH18KK285 | CGCG | 0.005 |
| 231 | MH06CP007 | TAT | 0.424 | 536 | MH18KK285 | CGCT | 0.147 |
| 232 | MH06KK026 | ACG | 0.005 | 537 | MH18KK285 | CGTG | 0.201 |
| 233 | MH06KK026 | ATG | 0.033 | 538 | MH18KK293 | AGAA | 0.315 |
| 234 | MH06KK026 | GCG | 0.940 | 539 | MH18KK293 | ATAA | 0.016 |
| 235 | MH06KK026 | GTG | 0.022 | 540 | MH18KK293 | ATGA | 0.114 |
| 236 | MH06KK030 | CAT | 0.391 | 541 | MH18KK293 | GGAA | 0.467 |
| 237 | MH06KK030 | TAT | 0.054 | 542 | MH18KK293 | GGAG | 0.071 |
| 238 | MH06KK030 | TCC | 0.554 | 543 | MH18KK293 | GGGA | 0.011 |
| 239 | MH06KK031 | AG | 0.179 | 544 | MH18KK293 | GTAA | 0.005 |
| 240 | MH06KK031 | GC | 0.810 | 545 | MH19CP007 | CAG | 0.223 |
| 241 | MH06KK080 | CG | 1.000 | 546 | MH19CP007 | CGA | 0.120 |
| 242 | MH06KK101 | AA | 0.853 | 547 | MH19CP007 | CGG | 0.326 |
| 243 | MH06KK101 | AG | 0.005 | 548 | MH19CP007 | GGG | 0.332 |
| 244 | MH06KK101 | GA | 0.011 | 549 | MH19KK056 | CA | 0.560 |
| 245 | MH06KK101 | GG | 0.130 | 550 | MH19KK056 | TA | 0.016 |
| 246 | MH07KK030 | ACC | 0.623 | 551 | MH19KK056 | TC | 0.424 |
| 247 | MH07KK030 | GAC | 0.228 | 552 | MH19KK057 | CCG | 0.663 |
| 248 | MH07KK030 | GCC | 0.148 | 553 | MH19KK057 | CTG | 0.283 |
| 249 | MH07KK031 | CA | 0.527 | 554 | MH19KK057 | CTT | 0.054 |
| 250 | MH07KK031 | CG | 0.201 | 555 | MH19KK299 | ATGAA | 0.103 |
| 251 | MH07KK031 | TG | 0.272 | 556 | MH19KK299 | GCAAA | 0.087 |
| 252 | MH07KK081 | DC | 0.005 | 557 | MH19KK299 | GCAAG | 0.446 |
| 253 | MH07KK081 | DT | 0.995 | 558 | MH19KK299 | GCATG | 0.250 |
| 254 | MH07KK082 | TC | 0.288 | 559 | MH19KK299 | GCGTA | 0.098 |
| 255 | MH07KK082 | TG | 0.712 | 560 | MH19KK299 | GCGTG | 0.016 |
| 256 | MH08KK032 | CG | 0.104 | 561 | MH19KK301 | GAAC | 0.842 |
| 257 | MH08KK032 | TA | 0.126 | 562 | MH19KK301 | GAAT | 0.005 |
| 258 | MH08KK032 | TG | 0.769 | 563 | MH19KK301 | GGAC | 0.005 |
| 259 | MH09KK020 | ACCC | 0.207 | 564 | MH19KK301 | GGAT | 0.147 |
| 260 | MH09KK020 | ACTC | 0.277 | 565 | MH20KK058 | CAC | 0.337 |
| 261 | MH09KK020 | GCTC | 0.516 | 566 | MH20KK058 | TAC | 0.277 |
| 262 | MH09KK033 | ACG | 0.315 | 567 | MH20KK058 | TAT | 0.266 |
| 263 | MH09KK033 | GCG | 0.348 | 568 | MH20KK058 | TGC | 0.120 |
| 264 | MH09KK033 | GTG | 0.337 | 569 | MH20KK059 | AA | 0.321 |
| 265 | MH09KK034 | AA | 0.005 | 570 | MH20KK059 | AG | 0.141 |
| 266 | MH09KK034 | GA | 0.207 | 571 | MH20KK059 | GG | 0.538 |
| 267 | MH09KK034 | GG | 0.788 | 572 | MH20KK307 | CTGA | 0.386 |
| 268 | MH09KK152 | AGCA | 0.198 | 573 | MH20KK307 | TCGA | 0.098 |
| 269 | MH09KK152 | ATTA | 0.088 | 574 | MH20KK307 | TTAA | 0.201 |
| 270 | MH09KK152 | ATTG | 0.527 | 575 | MH20KK307 | TTGA | 0.255 |
| 271 | MH09KK152 | GTCG | 0.187 | 576 | MH20KK307 | TTGC | 0.060 |
| 272 | MH09KK153 | CAA | 0.044 | 577 | MH21KK313 | CGT | 0.793 |
| 273 | MH09KK153 | CAC | 0.044 | 578 | MH21KK313 | CTC | 0.011 |
| 274 | MH09KK153 | CGA | 0.016 | 579 | MH21KK313 | TGT | 0.033 |
| 275 | MH09KK153 | CGC | 0.011 | 580 | MH21KK313 | TTT | 0.163 |
| 276 | MH09KK153 | TAA | 0.335 | 581 | MH21KK315 | ACC | 0.055 |
| 277 | MH09KK153 | TAC | 0.225 | 582 | MH21KK315 | ATC | 0.220 |
| 278 | MH09KK153 | TGA | 0.203 | 583 | MH21KK315 | ATT | 0.012 |
| 279 | MH09KK153 | TGC | 0.121 | 584 | MH21KK315 | GCC | 0.146 |
| 280 | MH09KK157 | ACCAT | 0.005 | 585 | MH21KK315 | GCT | 0.091 |
| 281 | MH09KK157 | ACTAT | 0.054 | 586 | MH21KK315 | GTC | 0.128 |
| 282 | MH09KK157 | GCCAC | 0.603 | 587 | MH21KK315 | GTT | 0.348 |
| 283 | MH09KK157 | GCCCT | 0.228 | 588 | MH21KK316 | ACAC | 0.462 |
| 284 | MH09KK157 | GTCAC | 0.109 | 589 | MH21KK316 | ACGC | 0.005 |
| 285 | MH09KK161 | CT | 0.223 | 590 | MH21KK316 | ACGT | 0.288 |
| 286 | MH09KK161 | TT | 0.777 | 591 | MH21KK316 | ATGC | 0.060 |
| 287 | MH10CP003 | CCA | 0.408 | 592 | MH21KK316 | GCGC | 0.185 |
| 288 | MH10CP003 | CCC | 0.293 | 593 | MH21KK324 | CCAA | 0.011 |
| 289 | MH10CP003 | CTA | 0.092 | 594 | MH21KK324 | CCAG | 0.011 |
| 290 | MH10CP003 | TCA | 0.207 | 595 | MH21KK324 | CCTA | 0.011 |
| 291 | MH10KK083 | GC | 0.087 | 596 | MH21KK324 | CTAA | 0.315 |
| 292 | MH10KK083 | TC | 0.913 | 597 | MH21KK324 | CTAG | 0.005 |
| 293 | MH10KK084 | TG | 1.000 | 598 | MH21KK324 | CTTA | 0.147 |
| 294 | MH10KK085 | CC | 0.473 | 599 | MH21KK324 | TCAA | 0.005 |
| 295 | MH10KK085 | CT | 0.527 | 600 | MH21KK324 | TCAG | 0.391 |
| 296 | MH10KK086 | GA | 0.549 | 601 | MH21KK324 | TCTG | 0.098 |
| 297 | MH10KK086 | GC | 0.370 | 602 | MH21KK324 | TTAA | 0.005 |
| 298 | MH10KK086 | TA | 0.082 | 603 | MH22KK060 | CA | 0.272 |
| 299 | MH10KK087 | AG | 0.739 | 604 | MH22KK060 | CG | 0.397 |
| 300 | MH10KK087 | GA | 0.261 | 605 | MH22KK060 | GG | 0.332 |
| 301 | MH10KK088 | GC | 0.832 | 606 | MH22KK064 | AATI | 0.837 |
| 302 | MH10KK088 | GT | 0.168 | 607 | MH22KK064 | GATI | 0.163 |
| 303 | MH10KK101 | AG | 0.462 | 608 | MH22KK303 | CGGG | 0.685 |
| 304 | MH10KK101 | CA | 0.152 | 609 | MH22KK303 | CTGG | 0.049 |
| 305 | MH10KK101 | CG | 0.386 | 610 | MH22KK303 | TGGG | 0.266 |

**Supplemental Table 7.** Forensic parameters of 163 MH loci of CHE samples (N=92).

| No. | MH | A_e_ | He | MP | DP | PE | TPI | HWE-*p* |
| --- | --- | --- | --- | --- | --- | --- | --- | --- |
| 1 | MH01CP007 | 1.6351 | 0.3043 | 0.4426 | 0.5574 | 0.0653 | 0.7188 | 0.0902 |
| 2 | MH01CP008 | 3.2825 | 0.6957 | 0.1489 | 0.8511 | 0.4216 | 1.6429 | 0.9416 |
| 3 | MH01CP012 | 3.0628 | 0.6413 | 0.1718 | 0.8282 | 0.3434 | 1.3939 | 0.4617 |
| 4 | MH01CP016 | 3.0778 | 0.7174 | 0.1928 | 0.8072 | 0.4557 | 1.7692 | 0.4277 |
| 5 | MH01KK001 | 2.7714 | 0.7065 | 0.2176 | 0.7824 | 0.4384 | 1.7037 | 0.2012 |
| 6 | MH01KK070 | 1.3892 | 0.2935 | 0.5555 | 0.4445 | 0.0609 | 0.7077 | 0.8020 |
| 7 | MH01KK072 | 1.8521 | 0.5000 | 0.4149 | 0.5851 | 0.1875 | 1.0000 | 0.4716 |
| 8 | MH01KK106 | 2.8518 | 0.6517 | 0.1713 | 0.8287 | 0.3575 | 1.4355 | 0.9789 |
| 9 | MH01KK117 | 4.2575 | 0.7174 | 0.0874 | 0.9126 | 0.4557 | 1.7692 | 0.2372 |
| 10 | MH01KK172 | 2.8996 | 0.6848 | 0.1907 | 0.8093 | 0.4051 | 1.5862 | 0.5979 |
| 11 | MH01KK205 | 4.1684 | 0.6739 | 0.0936 | 0.9064 | 0.3891 | 1.5333 | 0.0412 |
| 12 | MH01KK210 | 2.4551 | 0.6196 | 0.2389 | 0.7611 | 0.3150 | 1.3143 | 0.6440 |
| 13 | MH01KK211 | 3.0067 | 0.6087 | 0.1661 | 0.8339 | 0.3014 | 1.2778 | 0.2029 |
| 14 | MH02CP004 | 2.9771 | 0.6413 | 0.1741 | 0.8259 | 0.3434 | 1.3939 | 0.5904 |
| 15 | MH02KK003 | 1.3587 | 0.2717 | 0.5536 | 0.4464 | 0.0526 | 0.6866 | 0.8912 |
| 16 | MH02KK004 | 2.9829 | 0.7065 | 0.1812 | 0.8188 | 0.4384 | 1.7037 | 0.4372 |
| 17 | MH02KK073 | 1.5313 | 0.3696 | 0.4565 | 0.5435 | 0.0965 | 0.7931 | 0.6765 |
| 18 | MH02KK102 | 1.0000 | 0.0000 | 1.0000 | 0.0000 | 0.0000 | 0.5000 | NA |
| 19 | MH02KK105 | 2.5023 | 0.5870 | 0.2346 | 0.7654 | 0.2755 | 1.2105 | 0.7435 |
| 20 | MH02KK131 | 1.7177 | 0.4348 | 0.4249 | 0.5751 | 0.1365 | 0.8846 | 0.7756 |
| 21 | MH02KK134 | 3.4696 | 0.6739 | 0.1174 | 0.8826 | 0.3891 | 1.5333 | 0.3746 |
| 22 | MH02KK136 | 3.6357 | 0.6739 | 0.1274 | 0.8726 | 0.3891 | 1.5333 | 0.2353 |
| 23 | MH02KK138 | 2.7847 | 0.6522 | 0.2051 | 0.7949 | 0.3582 | 1.4375 | 0.8761 |
| 24 | MH02KK139 | 1.1289 | 0.1196 | 0.7824 | 0.2176 | 0.0116 | 0.5679 | 0.8863 |
| 25 | MH02KK201 | 1.1280 | 0.1196 | 0.7852 | 0.2148 | 0.0116 | 0.5679 | 0.8691 |
| 26 | MH02KK202 | 1.9482 | 0.4891 | 0.3830 | 0.6170 | 0.1782 | 0.9787 | 0.9964 |
| 27 | MH02KK213 | 2.1301 | 0.5000 | 0.3029 | 0.6971 | 0.1875 | 1.0000 | 0.5203 |
| 28 | MH02KK215 | 2.2016 | 0.5000 | 0.2724 | 0.7276 | 0.1875 | 1.0000 | 0.3472 |
| 29 | MH03KK006 | 1.9760 | 0.5652 | 0.3819 | 0.6181 | 0.2512 | 1.1500 | 0.1882 |
| 30 | MH03KK007 | 2.8927 | 0.7065 | 0.2068 | 0.7932 | 0.4384 | 1.7037 | 0.3254 |
| 31 | MH03KK008 | 2.1094 | 0.5109 | 0.3306 | 0.6694 | 0.1972 | 1.0222 | 0.7303 |
| 32 | MH03KK009 | 1.5754 | 0.3152 | 0.4686 | 0.5314 | 0.0700 | 0.7302 | 0.3005 |
| 33 | MH03KK216 | 2.0107 | 0.4457 | 0.3124 | 0.6876 | 0.1442 | 0.9020 | 0.2517 |
| 34 | MH04CP002 | 3.6601 | 0.7253 | 0.1298 | 0.8702 | 0.4684 | 1.8200 | 0.9054 |
| 35 | MH04CP003 | 3.5069 | 0.7065 | 0.1295 | 0.8705 | 0.4384 | 1.7037 | 0.7941 |
| 36 | MH04CP007 | 3.2992 | 0.7500 | 0.1640 | 0.8360 | 0.5098 | 2.0000 | 0.3018 |
| 37 | MH04KK010 | 1.8456 | 0.4565 | 0.3504 | 0.6496 | 0.1522 | 0.9200 | 0.9362 |
| 38 | MH04KK011 | 2.8902 | 0.6413 | 0.1935 | 0.8065 | 0.3434 | 1.3939 | 0.7422 |
| 39 | MH04KK013 | 2.5108 | 0.5217 | 0.2079 | 0.7921 | 0.2072 | 1.0455 | 0.1023 |
| 40 | MH04KK015 | 1.8235 | 0.4457 | 0.3419 | 0.6581 | 0.1442 | 0.9020 | 0.8709 |
| 41 | MH04KK016 | 1.8235 | 0.4457 | 0.3419 | 0.6581 | 0.1442 | 0.9020 | 0.8709 |
| 42 | MH04KK017 | 2.0851 | 0.4835 | 0.2699 | 0.7301 | 0.1735 | 0.9681 | 0.4475 |
| 43 | MH04KK019 | 1.9654 | 0.4022 | 0.3452 | 0.6548 | 0.1152 | 0.8364 | 0.0785 |
| 44 | MH04KK028 | 1.6277 | 0.3478 | 0.4480 | 0.5520 | 0.0852 | 0.7667 | 0.4320 |
| 45 | MH04KK029 | 1.2667 | 0.1957 | 0.6512 | 0.3488 | 0.0286 | 0.6216 | 0.7065 |
| 46 | MH04KK030 | 3.9991 | 0.7500 | 0.1243 | 0.8757 | 0.5098 | 2.0000 | 0.9283 |
| 47 | MH04KK074 | 1.1761 | 0.1630 | 0.7271 | 0.2729 | 0.0205 | 0.5974 | 0.7380 |
| 48 | MH05CP004 | 3.8808 | 0.7717 | 0.1137 | 0.8863 | 0.5477 | 2.1905 | 0.5761 |
| 49 | MH05CP006 | 3.1653 | 0.6413 | 0.1567 | 0.8433 | 0.3434 | 1.3939 | 0.3357 |
| 50 | MH05CP010 | 1.7375 | 0.3696 | 0.3977 | 0.6023 | 0.0965 | 0.7931 | 0.2673 |
| 51 | MH05KK020 | 3.2623 | 0.7065 | 0.1647 | 0.8353 | 0.4384 | 1.7037 | 0.8466 |
| 52 | MH05KK022 | 2.5951 | 0.6087 | 0.2169 | 0.7831 | 0.3014 | 1.2778 | 0.8539 |
| 53 | MH05KK062 | 2.6836 | 0.5652 | 0.2023 | 0.7977 | 0.2512 | 1.1500 | 0.1925 |
| 54 | MH05KK078 | 1.2934 | 0.2609 | 0.6144 | 0.3856 | 0.0487 | 0.6765 | 0.4536 |
| 55 | MH05KK079 | 1.9665 | 0.4348 | 0.3573 | 0.6427 | 0.1365 | 0.8846 | 0.2545 |
| 56 | MH05KK122 | 1.7963 | 0.4783 | 0.3507 | 0.6493 | 0.1692 | 0.9583 | 0.5299 |
| 57 | MH05KK123 | 2.8436 | 0.6413 | 0.1954 | 0.8046 | 0.3434 | 1.3939 | 0.8314 |
| 58 | MH05KK124 | 2.0334 | 0.4783 | 0.2980 | 0.7020 | 0.1692 | 0.9583 | 0.5300 |
| 59 | MH05KK170 | 6.0544 | 0.8696 | 0.0598 | 0.9402 | 0.7338 | 3.8333 | 0.4306 |
| 60 | MH06CP003 | 3.5333 | 0.6739 | 0.1252 | 0.8748 | 0.3891 | 1.5333 | 0.3151 |
| 61 | MH06CP007 | 3.4660 | 0.7500 | 0.1493 | 0.8507 | 0.5098 | 2.0000 | 0.4617 |
| 62 | MH06KK026 | 1.1292 | 0.1196 | 0.7814 | 0.2186 | 0.0116 | 0.5679 | 0.8921 |
| 63 | MH06KK030 | 2.1581 | 0.5109 | 0.3098 | 0.6902 | 0.1972 | 1.0222 | 0.5809 |
| 64 | MH06KK031 | 1.4537 | 0.2935 | 0.5447 | 0.4553 | 0.0609 | 0.7077 | 0.6746 |
| 65 | MH06KK080 | 1.0000 | 0.0000 | 1.0000 | 0.0000 | 0.0000 | 0.5000 | NA |
| 66 | MH06KK101 | 1.3419 | 0.2717 | 0.5725 | 0.4275 | 0.0526 | 0.6866 | 0.7324 |
| 67 | MH07KK030 | 2.1607 | 0.4815 | 0.2815 | 0.7185 | 0.1718 | 0.9643 | 0.2863 |
| 68 | MH07KK031 | 2.5498 | 0.6304 | 0.2254 | 0.7746 | 0.3290 | 1.3529 | 0.7041 |
| 69 | MH07KK081 | 1.0109 | 0.0109 | 0.9785 | 0.0215 | 0.0001 | 0.5055 | 1.0000 |
| 70 | MH07KK082 | 1.6953 | 0.4674 | 0.4501 | 0.5499 | 0.1605 | 0.9388 | 0.2839 |
| 71 | MH08KK032 | 1.6166 | 0.3407 | 0.4249 | 0.5751 | 0.0817 | 0.7583 | 0.4004 |
| 72 | MH09KK020 | 2.5904 | 0.6304 | 0.2212 | 0.7788 | 0.3290 | 1.3529 | 0.7956 |
| 73 | MH09KK033 | 2.9950 | 0.6304 | 0.1793 | 0.8207 | 0.3290 | 1.3529 | 0.4226 |
| 74 | MH09KK034 | 1.5067 | 0.3043 | 0.5005 | 0.4995 | 0.0653 | 0.7188 | 0.4932 |
| 75 | MH09KK152 | 2.7779 | 0.6044 | 0.1781 | 0.8219 | 0.2962 | 1.2639 | 0.4354 |
| 76 | MH09KK153 | 4.4786 | 0.7363 | 0.0929 | 0.9071 | 0.4866 | 1.8958 | 0.3020 |
| 77 | MH09KK157 | 2.3211 | 0.5652 | 0.2500 | 0.7500 | 0.2512 | 1.1500 | 0.8910 |
| 78 | MH09KK161 | 1.5299 | 0.3804 | 0.4903 | 0.5097 | 0.1025 | 0.8070 | 0.5169 |
| 79 | MH10CP003 | 3.2953 | 0.7500 | 0.1560 | 0.8440 | 0.5098 | 2.0000 | 0.2985 |
| 80 | MH10KK083 | 1.1888 | 0.1739 | 0.7127 | 0.2873 | 0.0231 | 0.6053 | 0.7089 |
| 81 | MH10KK084 | 1.0000 | 0.0000 | 1.0000 | 0.0000 | 0.0000 | 0.5000 | NA |
| 82 | MH10KK085 | 1.9941 | 0.5543 | 0.4081 | 0.5919 | 0.2396 | 1.1220 | 0.3084 |
| 83 | MH10KK086 | 2.2496 | 0.6087 | 0.3036 | 0.6964 | 0.3014 | 1.2778 | 0.3323 |
| 84 | MH10KK087 | 1.6277 | 0.4348 | 0.4631 | 0.5369 | 0.1365 | 0.8846 | 0.3544 |
| 85 | MH10KK088 | 1.3892 | 0.2717 | 0.5588 | 0.4412 | 0.0526 | 0.6866 | 0.8315 |
| 86 | MH10KK101 | 2.5943 | 0.5652 | 0.2122 | 0.7878 | 0.2512 | 1.1500 | 0.2983 |
| 87 | MH10KK163 | 4.2341 | 0.7391 | 0.0950 | 0.9050 | 0.4914 | 1.9167 | 0.5119 |
| 88 | MH10KK170 | 1.2316 | 0.1848 | 0.6713 | 0.3287 | 0.0258 | 0.6133 | 0.9166 |
| 89 | MH11CP003 | 3.5766 | 0.7283 | 0.1444 | 0.8556 | 0.4733 | 1.8400 | 0.9329 |
| 90 | MH11CP004 | 2.9756 | 0.6413 | 0.1571 | 0.8429 | 0.3434 | 1.3939 | 0.5930 |
| 91 | MH11CP005 | 3.4205 | 0.6739 | 0.1415 | 0.8585 | 0.3891 | 1.5333 | 0.4260 |
| 92 | MH11KK036 | 2.9302 | 0.6522 | 0.1919 | 0.8081 | 0.3582 | 1.4375 | 0.8368 |
| 93 | MH11KK037 | 2.4392 | 0.6087 | 0.2488 | 0.7512 | 0.3014 | 1.2778 | 0.7630 |
| 94 | MH11KK038 | 2.1916 | 0.5000 | 0.2826 | 0.7174 | 0.1875 | 1.0000 | 0.3684 |
| 95 | MH11KK039 | 2.2145 | 0.5870 | 0.3259 | 0.6741 | 0.2755 | 1.2105 | 0.4933 |
| 96 | MH11KK040 | 2.2520 | 0.5217 | 0.2840 | 0.7160 | 0.2072 | 1.0455 | 0.4719 |
| 97 | MH11KK041 | 2.2424 | 0.5326 | 0.2876 | 0.7124 | 0.2177 | 1.0698 | 0.6365 |
| 98 | MH11KK089 | 2.0398 | 0.4674 | 0.3424 | 0.6576 | 0.1605 | 0.9388 | 0.3864 |
| 99 | MH11KK090 | 1.9665 | 0.5217 | 0.3951 | 0.6049 | 0.2072 | 1.0455 | 0.5970 |
| 100 | MH11KK091 | 1.8228 | 0.4348 | 0.3533 | 0.6467 | 0.1365 | 0.8846 | 0.7134 |
| 101 | MH11KK180 | 3.6186 | 0.7500 | 0.1172 | 0.8828 | 0.5098 | 2.0000 | 0.6295 |
| 102 | MH11KK187 | 2.8716 | 0.6630 | 0.1942 | 0.8058 | 0.3734 | 1.4839 | 0.8762 |
| 103 | MH11KK191 | 2.9543 | 0.6512 | 0.1682 | 0.8318 | 0.3568 | 1.4333 | 0.7800 |
| 104 | MH12KK042 | 2.0514 | 0.4891 | 0.3403 | 0.6597 | 0.1782 | 0.9787 | 0.6152 |
| 105 | MH12KK043 | 2.5460 | 0.6374 | 0.2520 | 0.7480 | 0.3381 | 1.3788 | 0.6004 |
| 106 | MH12KK045 | 1.7059 | 0.4565 | 0.4005 | 0.5995 | 0.1522 | 0.9200 | 0.4312 |
| 107 | MH12KK046 | 3.9226 | 0.7609 | 0.1219 | 0.8781 | 0.5285 | 2.0909 | 0.7952 |
| 108 | MH12KK092 | 1.7841 | 0.3913 | 0.3989 | 0.6011 | 0.1087 | 0.8214 | 0.3284 |
| 109 | MH12KK093 | 1.6000 | 0.4130 | 0.4679 | 0.5321 | 0.1221 | 0.8519 | 0.4762 |
| 110 | MH12KK202 | 3.5811 | 0.7826 | 0.1451 | 0.8549 | 0.5672 | 2.3000 | 0.2137 |
| 111 | MH13CP008 | 3.0987 | 0.6630 | 0.1616 | 0.8384 | 0.3734 | 1.4839 | 0.7120 |
| 112 | MH13KK047 | 2.1683 | 0.5326 | 0.2760 | 0.7240 | 0.2177 | 1.0698 | 0.8602 |
| 113 | MH13KK213 | 3.8781 | 0.7935 | 0.1302 | 0.8698 | 0.5870 | 2.4211 | 0.2974 |
| 114 | MH13KK217 | 4.5628 | 0.7609 | 0.0832 | 0.9168 | 0.5285 | 2.0909 | 0.5715 |
| 115 | MH13KK218 | 7.0814 | 0.7935 | 0.0435 | 0.9565 | 0.5870 | 2.4211 | 0.0505 |
| 116 | MH13KK225 | 3.1897 | 0.7283 | 0.1664 | 0.8336 | 0.4733 | 1.8400 | 0.4304 |
| 117 | MH13KK226 | 1.8674 | 0.4783 | 0.3821 | 0.6179 | 0.1692 | 0.9583 | 0.8291 |
| 118 | MH14CP003 | 3.1021 | 0.6522 | 0.1524 | 0.8476 | 0.3582 | 1.4375 | 0.5483 |
| 119 | MH14CP004 | 3.7493 | 0.7935 | 0.1259 | 0.8741 | 0.5870 | 2.4211 | 0.2207 |
| 120 | MH14KK048 | 1.9184 | 0.4348 | 0.3346 | 0.6654 | 0.1365 | 0.8846 | 0.3714 |
| 121 | MH14KK101 | 1.2934 | 0.2391 | 0.6198 | 0.3802 | 0.0414 | 0.6571 | 0.8006 |
| 122 | MH15CP001 | 3.5257 | 0.6515 | 0.1299 | 0.8701 | 0.3573 | 1.4348 | 0.2023 |
| 123 | MH15CP003 | 2.2904 | 0.5761 | 0.2436 | 0.7564 | 0.2632 | 1.1795 | 0.8523 |
| 124 | MH15CP004 | 2.7003 | 0.6739 | 0.1990 | 0.8010 | 0.3891 | 1.5333 | 0.4167 |
| 125 | MH15KK066 | 3.2940 | 0.6630 | 0.1394 | 0.8606 | 0.3734 | 1.4839 | 0.4363 |
| 126 | MH15KK067 | 2.3577 | 0.5326 | 0.2493 | 0.7507 | 0.2177 | 1.0698 | 0.3675 |
| 127 | MH15KK069 | 1.5668 | 0.3478 | 0.4440 | 0.5560 | 0.0852 | 0.7667 | 0.7510 |
| 128 | MH15KK095 | 2.2239 | 0.5326 | 0.2897 | 0.7103 | 0.2177 | 1.0698 | 0.6892 |
| 129 | MH16KK053 | 1.4875 | 0.2826 | 0.5095 | 0.4905 | 0.0566 | 0.6970 | 0.3383 |
| 130 | MH16KK062 | 2.7911 | 0.6630 | 0.2209 | 0.7791 | 0.3734 | 1.4839 | 0.7209 |
| 131 | MH16KK096 | 1.7961 | 0.3804 | 0.3934 | 0.6066 | 0.1025 | 0.8070 | 0.2082 |
| 132 | MH16KK255 | 4.3350 | 0.7935 | 0.0943 | 0.9057 | 0.5870 | 2.4211 | 0.6474 |
| 133 | MH16KK302 | 3.8238 | 0.7717 | 0.1222 | 0.8778 | 0.5477 | 2.1905 | 0.5215 |
| 134 | MH17CP001 | 3.2883 | 0.6957 | 0.1510 | 0.8490 | 0.4216 | 1.6429 | 0.9326 |
| 135 | MH17CP006 | 3.1227 | 0.6630 | 0.1633 | 0.8367 | 0.3734 | 1.4839 | 0.6735 |
| 136 | MH17KK014 | 1.0799 | 0.0761 | 0.8561 | 0.1439 | 0.0050 | 0.5412 | 0.9515 |
| 137 | MH17KK052 | 2.7534 | 0.6630 | 0.2075 | 0.7925 | 0.3734 | 1.4839 | 0.6494 |
| 138 | MH17KK053 | 2.6597 | 0.6706 | 0.2307 | 0.7693 | 0.3842 | 1.5179 | 0.4135 |
| 139 | MH17KK054 | 2.7194 | 0.6739 | 0.2186 | 0.7814 | 0.3891 | 1.5333 | 0.4465 |
| 140 | MH17KK055 | 2.2592 | 0.6413 | 0.3240 | 0.6760 | 0.3434 | 1.3939 | 0.1180 |
| 141 | MH17KK077 | 1.2403 | 0.2174 | 0.6597 | 0.3403 | 0.0347 | 0.6389 | 0.5846 |
| 142 | MH17KK105 | 1.0584 | 0.0568 | 0.8928 | 0.1072 | 0.0029 | 0.5301 | 0.9576 |
| 143 | MH17KK110 | 1.2143 | 0.1739 | 0.6949 | 0.3051 | 0.0231 | 0.6053 | 0.9287 |
| 144 | MH17KK272 | 3.2615 | 0.7473 | 0.1532 | 0.8468 | 0.5051 | 1.9783 | 0.2990 |
| 145 | MH18CP003 | 3.4939 | 0.7283 | 0.1385 | 0.8615 | 0.4733 | 1.8400 | 0.8218 |
| 146 | MH18CP005 | 3.6953 | 0.7174 | 0.1281 | 0.8719 | 0.4557 | 1.7692 | 0.7289 |
| 147 | MH18KK285 | 2.7879 | 0.6630 | 0.1784 | 0.8216 | 0.3734 | 1.4839 | 0.7148 |
| 148 | MH18KK293 | 2.9740 | 0.6196 | 0.1690 | 0.8310 | 0.3150 | 1.3143 | 0.3304 |
| 149 | MH19CP007 | 3.5690 | 0.6848 | 0.1307 | 0.8693 | 0.4051 | 1.5862 | 0.4033 |
| 150 | MH19KK056 | 2.0271 | 0.4783 | 0.3438 | 0.6562 | 0.1692 | 0.9583 | 0.5496 |
| 151 | MH19KK057 | 1.9141 | 0.5000 | 0.3466 | 0.6534 | 0.1875 | 1.0000 | 0.7033 |
| 152 | MH19KK299 | 3.4582 | 0.7717 | 0.1406 | 0.8594 | 0.5477 | 2.1905 | 0.2258 |
| 153 | MH19KK301 | 1.3676 | 0.2283 | 0.5751 | 0.4249 | 0.0379 | 0.6479 | 0.3644 |
| 154 | MH20KK058 | 3.6287 | 0.7065 | 0.1352 | 0.8648 | 0.4384 | 1.7037 | 0.6374 |
| 155 | MH20KK059 | 2.4256 | 0.4891 | 0.2304 | 0.7696 | 0.1782 | 0.9787 | 0.0470 |
| 156 | MH20KK307 | 3.7352 | 0.7174 | 0.1170 | 0.8830 | 0.4557 | 1.7692 | 0.6810 |
| 157 | MH21KK313 | 1.5212 | 0.3261 | 0.4690 | 0.5310 | 0.0748 | 0.7419 | 0.7102 |
| 158 | MH21KK315 | 4.5804 | 0.7439 | 0.0836 | 0.9164 | 0.4994 | 1.9524 | 0.3469 |
| 159 | MH21KK316 | 2.9929 | 0.7065 | 0.1876 | 0.8124 | 0.4384 | 1.7037 | 0.4505 |
| 160 | MH21KK324 | 3.5208 | 0.7826 | 0.1522 | 0.8478 | 0.5672 | 2.3000 | 0.1803 |
| 161 | MH22KK060 | 2.9313 | 0.6087 | 0.1829 | 0.8171 | 0.3014 | 1.2778 | 0.2756 |
| 162 | MH22KK064 | 1.3754 | 0.3043 | 0.5617 | 0.4383 | 0.0653 | 0.7188 | 0.5199 |
| 163 | MH22KK303 | 1.8442 | 0.4457 | 0.3547 | 0.6453 | 0.1442 | 0.9020 | 0.7786 |

NA: Heterozygous alleles were not detected at MH02KK102, MH06KK080 and MH10KK084.

**Supplementary Table 8.** I_n_ values of 163 MH loci.

| No. | MH | I_n_ | No. | MH | I_n_ |
| --- | --- | --- | --- | --- | --- |
| 1 | MH01CP007 | 0.0666 | 83 | MH10KK086 | 0.1402 |
| 2 | MH01CP008 | 0.1268 | 84 | MH10KK087 | 0.0220 |
| 3 | MH01CP012 | 0.1780 | 85 | MH10KK088 | 0.1137 |
| 4 | MH01CP016 | 0.0800 | 86 | MH10KK101 | 0.1041 |
| 5 | MH01KK001 | 0.2959 | 87 | MH10KK163 | 0.2381 |
| 6 | MH01KK070 | 0.1406 | 88 | MH10KK170 | 0.2628 |
| 7 | MH01KK072 | 0.0242 | 89 | MH11CP003 | 0.0449 |
| 8 | MH01KK106 | 0.1991 | 90 | MH11CP004 | 0.0701 |
| 9 | MH01KK117 | 0.1948 | 91 | MH11CP005 | 0.1484 |
| 10 | MH01KK172 | 0.2181 | 92 | MH11KK036 | 0.0568 |
| 11 | MH01KK205 | 0.0732 | 93 | MH11KK037 | 0.1496 |
| 12 | MH01KK210 | 0.1588 | 94 | MH11KK038 | 0.0975 |
| 13 | MH01KK211 | 0.1666 | 95 | MH11KK039 | 0.0992 |
| 14 | MH02CP004 | 0.0842 | 96 | MH11KK040 | 0.1779 |
| 15 | MH02KK003 | 0.3154 | 97 | MH11KK041 | 0.1210 |
| 16 | MH02KK004 | 0.1645 | 98 | MH11KK089 | 0.0815 |
| 17 | MH02KK073 | 0.2310 | 99 | MH11KK090 | 0.0766 |
| 18 | MH02KK102 | 0.1559 | 100 | MH11KK091 | 0.1437 |
| 19 | MH02KK105 | 0.1984 | 101 | MH11KK180 | 0.2674 |
| 20 | MH02KK131 | 0.2660 | 102 | MH11KK187 | 0.2475 |
| 21 | MH02KK134 | 0.2784 | 103 | MH11KK191 | 0.1803 |
| 22 | MH02KK136 | 0.1205 | 104 | MH12KK042 | 0.2421 |
| 23 | MH02KK138 | 0.2639 | 105 | MH12KK043 | 0.1116 |
| 24 | MH02KK139 | 0.4637 | 106 | MH12KK045 | 0.2185 |
| 25 | MH02KK201 | 0.2163 | 107 | MH12KK046 | 0.1315 |
| 26 | MH02KK202 | 0.0598 | 108 | MH12KK092 | 0.0497 |
| 27 | MH02KK213 | 0.1447 | 109 | MH12KK093 | 0.1895 |
| 28 | MH02KK215 | 0.1239 | 110 | MH12KK202 | 0.0954 |
| 29 | MH03KK006 | 0.0972 | 111 | MH13CP008 | 0.1688 |
| 30 | MH03KK007 | 0.1027 | 112 | MH13KK047 | 0.2080 |
| 31 | MH03KK008 | 0.1721 | 113 | MH13KK213 | 0.1556 |
| 32 | MH03KK009 | 0.1118 | 114 | MH13KK217 | 0.1647 |
| 33 | MH03KK216 | 0.2671 | 115 | MH13KK218 | 0.2664 |
| 34 | MH04CP002 | 0.0609 | 116 | MH13KK225 | 0.1235 |
| 35 | MH04CP003 | 0.1685 | 117 | MH13KK226 | 0.2203 |
| 36 | MH04CP007 | 0.0560 | 118 | MH14CP003 | 0.1512 |
| 37 | MH04KK010 | 0.1365 | 119 | MH14CP004 | 0.1051 |
| 38 | MH04KK011 | 0.1187 | 120 | MH14KK048 | 0.1770 |
| 39 | MH04KK013 | 0.1721 | 121 | MH14KK101 | 0.3653 |
| 40 | MH04KK015 | 0.1119 | 122 | MH15CP001 | 0.0665 |
| 41 | MH04KK016 | 0.1131 | 123 | MH15CP003 | 0.1875 |
| 42 | MH04KK017 | 0.1480 | 124 | MH15CP004 | 0.2322 |
| 43 | MH04KK019 | 0.1352 | 125 | MH15KK066 | 0.1360 |
| 44 | MH04KK028 | 0.1030 | 126 | MH15KK067 | 0.2162 |
| 45 | MH04KK029 | 0.0564 | 127 | MH15KK069 | 0.2347 |
| 46 | MH04KK030 | 0.2402 | 128 | MH15KK095 | 0.1903 |
| 47 | MH04KK074 | 0.1925 | 129 | MH16KK053 | 0.4525 |
| 48 | MH05CP004 | 0.1112 | 130 | MH16KK062 | 0.3610 |
| 49 | MH05CP006 | 0.1398 | 131 | MH16KK096 | 0.2263 |
| 50 | MH05CP010 | 0.1204 | 132 | MH16KK255 | 0.1713 |
| 51 | MH05KK020 | 0.1032 | 133 | MH16KK302 | 0.2508 |
| 52 | MH05KK022 | 0.0930 | 134 | MH17CP001 | 0.0670 |
| 53 | MH05KK062 | 0.1300 | 135 | MH17CP006 | 0.0581 |
| 54 | MH05KK078 | 0.0278 | 136 | MH17KK014 | 0.6504 |
| 55 | MH05KK079 | 0.1164 | 137 | MH17KK052 | 0.0853 |
| 56 | MH05KK122 | 0.2695 | 138 | MH17KK053 | 0.0601 |
| 57 | MH05KK123 | 0.1560 | 139 | MH17KK054 | 0.0523 |
| 58 | MH05KK124 | 0.2900 | 140 | MH17KK055 | 0.0322 |
| 59 | MH05KK170 | 0.3566 | 141 | MH17KK077 | 0.1024 |
| 60 | MH06CP003 | 0.1288 | 142 | MH17KK105 | 0.0709 |
| 61 | MH06CP007 | 0.0932 | 143 | MH17KK110 | 0.0660 |
| 62 | MH06KK026 | 0.1434 | 144 | MH17KK272 | 0.1095 |
| 63 | MH06KK030 | 0.1585 | 145 | MH18CP003 | 0.1033 |
| 64 | MH06KK031 | 0.2332 | 146 | MH18CP005 | 0.0931 |
| 65 | MH06KK080 | 0.0803 | 147 | MH18KK285 | 0.1762 |
| 66 | MH06KK101 | 0.2423 | 148 | MH18KK293 | 0.2449 |
| 67 | MH07KK030 | 0.0519 | 149 | MH19CP007 | 0.0460 |
| 68 | MH07KK031 | 0.0643 | 150 | MH19KK056 | 0.0811 |
| 69 | MH07KK081 | 0.0652 | 151 | MH19KK057 | 0.0683 |
| 70 | MH07KK082 | 0.0459 | 152 | MH19KK299 | 0.2332 |
| 71 | MH08KK032 | 0.1422 | 153 | MH19KK301 | 0.2790 |
| 72 | MH09KK020 | 0.3278 | 154 | MH20KK058 | 0.1226 |
| 73 | MH09KK033 | 0.1018 | 155 | MH20KK059 | 0.0290 |
| 74 | MH09KK034 | 0.1736 | 156 | MH20KK307 | 0.1504 |
| 75 | MH09KK152 | 0.1656 | 157 | MH21KK313 | 0.2698 |
| 76 | MH09KK153 | 0.2831 | 158 | MH21KK315 | 0.1283 |
| 77 | MH09KK157 | 0.2167 | 159 | MH21KK316 | 0.2538 |
| 78 | MH09KK161 | 0.4349 | 160 | MH21KK324 | 0.3064 |
| 79 | MH10CP003 | 0.1984 | 161 | MH22KK060 | 0.0758 |
| 80 | MH10KK083 | 0.0480 | 162 | MH22KK064 | 0.1750 |
| 81 | MH10KK084 | 0.0738 | 163 | MH22KK303 | 0.0424 |
| 82 | MH10KK085 | 0.1094 |  |  |  |

**Supplementary Table 9.** Paired *F_st_* values among the 27 populations.

|  | ACB | ESN | GWD | YRI | LWK | ASW | MSL | GBR | CEU | FIN | IBS | TSI | BEB | GIH | PJL | STU | ITU | CDX | CHB | CHS | CHE | JPT | KHV | MXL | PUR | CLM | PEL |
| --- | --- | --- | --- | --- | --- | --- | --- | --- | --- | --- | --- | --- | --- | --- | --- | --- | --- | --- | --- | --- | --- | --- | --- | --- | --- | --- | --- |
| ACB | 0.000 |  |  |  |  |  |  |  |  |  |  |  |  |  |  |  |  |  |  |  |  |  |  |  |  |  |  |
| ESN | 0.010 | 0.000 |  |  |  |  |  |  |  |  |  |  |  |  |  |  |  |  |  |  |  |  |  |  |  |  |  |
| GWD | 0.014 | 0.014 | 0.000 |  |  |  |  |  |  |  |  |  |  |  |  |  |  |  |  |  |  |  |  |  |  |  |  |
| YRI | 0.009 | 0.007 | 0.013 | 0.000 |  |  |  |  |  |  |  |  |  |  |  |  |  |  |  |  |  |  |  |  |  |  |  |
| LWK | 0.012 | 0.017 | 0.019 | 0.015 | 0.000 |  |  |  |  |  |  |  |  |  |  |  |  |  |  |  |  |  |  |  |  |  |  |
| ASW | 0.009 | 0.019 | 0.019 | 0.017 | 0.017 | 0.000 |  |  |  |  |  |  |  |  |  |  |  |  |  |  |  |  |  |  |  |  |  |
| MSL | 0.011 | 0.014 | 0.012 | 0.011 | 0.017 | 0.018 | 0.000 |  |  |  |  |  |  |  |  |  |  |  |  |  |  |  |  |  |  |  |  |
| GBR | 0.146 | 0.181 | 0.178 | 0.179 | 0.164 | 0.115 | 0.177 | 0.000 |  |  |  |  |  |  |  |  |  |  |  |  |  |  |  |  |  |  |  |
| CEU | 0.148 | 0.183 | 0.179 | 0.181 | 0.165 | 0.116 | 0.179 | 0.006 | 0.000 |  |  |  |  |  |  |  |  |  |  |  |  |  |  |  |  |  |  |
| FIN | 0.147 | 0.180 | 0.178 | 0.178 | 0.164 | 0.116 | 0.178 | 0.013 | 0.013 | 0.000 |  |  |  |  |  |  |  |  |  |  |  |  |  |  |  |  |  |
| IBS | 0.140 | 0.174 | 0.170 | 0.171 | 0.155 | 0.108 | 0.169 | 0.009 | 0.009 | 0.017 | 0.000 |  |  |  |  |  |  |  |  |  |  |  |  |  |  |  |  |
| TSI | 0.146 | 0.181 | 0.177 | 0.178 | 0.162 | 0.114 | 0.176 | 0.012 | 0.011 | 0.021 | 0.007 | 0.000 |  |  |  |  |  |  |  |  |  |  |  |  |  |  |  |
| BEB | 0.124 | 0.148 | 0.149 | 0.144 | 0.134 | 0.099 | 0.146 | 0.083 | 0.084 | 0.077 | 0.082 | 0.084 | 0.000 |  |  |  |  |  |  |  |  |  |  |  |  |  |  |
| GIH | 0.122 | 0.148 | 0.149 | 0.145 | 0.133 | 0.096 | 0.146 | 0.066 | 0.066 | 0.061 | 0.063 | 0.065 | 0.011 | 0.000 |  |  |  |  |  |  |  |  |  |  |  |  |  |
| PJL | 0.118 | 0.143 | 0.143 | 0.140 | 0.128 | 0.093 | 0.142 | 0.062 | 0.063 | 0.059 | 0.060 | 0.062 | 0.010 | 0.009 | 0.000 |  |  |  |  |  |  |  |  |  |  |  |  |
| STU | 0.123 | 0.147 | 0.147 | 0.144 | 0.132 | 0.099 | 0.145 | 0.084 | 0.085 | 0.078 | 0.081 | 0.082 | 0.008 | 0.011 | 0.011 | 0.000 |  |  |  |  |  |  |  |  |  |  |  |
| ITU | 0.128 | 0.152 | 0.152 | 0.149 | 0.137 | 0.103 | 0.150 | 0.081 | 0.082 | 0.075 | 0.078 | 0.079 | 0.008 | 0.010 | 0.010 | 0.007 | 0.000 |  |  |  |  |  |  |  |  |  |  |
| CDX | 0.191 | 0.209 | 0.212 | 0.206 | 0.198 | 0.170 | 0.206 | 0.205 | 0.205 | 0.188 | 0.204 | 0.208 | 0.098 | 0.125 | 0.122 | 0.118 | 0.120 | 0.000 |  |  |  |  |  |  |  |  |  |
| CHB | 0.191 | 0.208 | 0.212 | 0.205 | 0.198 | 0.169 | 0.207 | 0.207 | 0.206 | 0.189 | 0.205 | 0.210 | 0.098 | 0.125 | 0.122 | 0.118 | 0.119 | 0.013 | 0.000 |  |  |  |  |  |  |  |  |
| CHS | 0.187 | 0.204 | 0.207 | 0.201 | 0.194 | 0.166 | 0.203 | 0.201 | 0.201 | 0.183 | 0.199 | 0.204 | 0.094 | 0.119 | 0.118 | 0.113 | 0.115 | 0.009 | 0.006 | 0.000 |  |  |  |  |  |  |  |
| CHL | 0.193 | 0.211 | 0.215 | 0.208 | 0.201 | 0.173 | 0.209 | 0.208 | 0.207 | 0.190 | 0.206 | 0.211 | 0.099 | 0.126 | 0.123 | 0.119 | 0.120 | 0.017 | 0.009 | 0.009 | 0.000 |  |  |  |  |  |  |
| JPT | 0.189 | 0.207 | 0.211 | 0.204 | 0.197 | 0.168 | 0.206 | 0.203 | 0.201 | 0.185 | 0.200 | 0.205 | 0.093 | 0.118 | 0.117 | 0.113 | 0.114 | 0.024 | 0.013 | 0.015 | 0.017 | 0.000 |  |  |  |  |  |
| KHV | 0.182 | 0.199 | 0.203 | 0.197 | 0.190 | 0.161 | 0.198 | 0.195 | 0.195 | 0.179 | 0.194 | 0.199 | 0.091 | 0.117 | 0.115 | 0.111 | 0.112 | 0.006 | 0.011 | 0.008 | 0.015 | 0.021 | 0.000 |  |  |  |  |
| MXL | 0.131 | 0.158 | 0.157 | 0.155 | 0.143 | 0.100 | 0.154 | 0.065 | 0.064 | 0.061 | 0.061 | 0.067 | 0.059 | 0.058 | 0.052 | 0.064 | 0.065 | 0.124 | 0.122 | 0.120 | 0.125 | 0.120 | 0.114 | 0.000 |  |  |  |
| PUR | 0.099 | 0.128 | 0.125 | 0.125 | 0.112 | 0.070 | 0.124 | 0.023 | 0.023 | 0.026 | 0.017 | 0.021 | 0.057 | 0.046 | 0.042 | 0.058 | 0.057 | 0.158 | 0.159 | 0.154 | 0.160 | 0.154 | 0.149 | 0.032 | 0.000 |  |  |
| CLM | 0.106 | 0.135 | 0.133 | 0.132 | 0.120 | 0.077 | 0.132 | 0.030 | 0.029 | 0.031 | 0.026 | 0.031 | 0.054 | 0.046 | 0.042 | 0.056 | 0.057 | 0.144 | 0.143 | 0.139 | 0.145 | 0.138 | 0.135 | 0.020 | 0.011 | 0.000 |  |
| PEL | 0.178 | 0.200 | 0.200 | 0.197 | 0.187 | 0.146 | 0.199 | 0.144 | 0.145 | 0.134 | 0.142 | 0.150 | 0.104 | 0.110 | 0.105 | 0.113 | 0.116 | 0.134 | 0.132 | 0.131 | 0.135 | 0.129 | 0.125 | 0.033 | 0.092 | 0.069 | 0.000 |

ACB: African Caribbeans in Barbados, ESN: Esan in Nigeria, GWD: Gambian in Western Division in Gambia, YRI: Yoruba in ibadan, Nigeria, LWK: luhya in webuye, Kenya, ASW: Americans of African ancestry in south-western USA, MSL: Mende in Sierra Leone, GBR: British in England and Scotland, CEU: Residents of Utah, USA with Northern and Western European Ancestry, FIN: Finnish in Finland, IBS: Iberian population in Spain, TSI: Toscani in Italy, BEB: Bengali from Bangladesh, GIH: Gujarati Indian from Houston, Texas, USA, PJL: Punjabi from Lahore, Pakistan, STU: Sri Lankan Tamil from the UK, ITU: Indian Telugu from the UK, CDX: Chinese Dai in Xishuangbanna, CHB: Han Chinese in Beijing, CHS: Han Chinese in Southern China, CHE: Han Chinese in Eastern China, JPT: Japanese in Tokyo, Japan, KHV: Kinh in Ho Chi Minh City Vietnam, MXL: Mexican ancestry from Los Angeles, USA, PUR: Puerto ricans from Puerto Rico, CLM: Colombians from Medellin, Colombia, PEL: Peruvians from Lima, Peru.
